# The evolving doublecortin (DCX) superfamily

**DOI:** 10.1186/1471-2164-7-188

**Published:** 2006-07-26

**Authors:** Orly Reiner, Frédéric M Coquelle, Bastian Peter, Talia Levy, Anna Kaplan, Tamar Sapir, Irit Orr, Naama Barkai, Gregor Eichele, Sven Bergmann

**Affiliations:** 1 Department of Molecular Genetics, Weizmann Institute of Science, Rehovot, Israel; 2 Department of Medical Genetics, University of Lausanne, Switzerland; 3 Department of Biological Services, Weizmann Institute of Science, Rehovot, Israel; 4 Max-Planck Institute, Hannover, Germany; 5 CNRS – UMR 6026, Université de Rennes 1, Equipe SDM, Campus de Beaulieu – Bât. 13, 35042 Rennes cedex, France

## Abstract

**Background:**

Doublecortin (DCX) domains serve as protein-interaction platforms. Mutations in members of this protein superfamily are linked to several genetic diseases. Mutations in the human *DCX *gene result in abnormal neuronal migration, epilepsy, and mental retardation; mutations in *RP1 *are associated with a form of inherited blindness, and *DCDC2 *has been associated with dyslectic reading disabilities.

**Results:**

The DCX-repeat gene family is composed of eleven paralogs in human and in mouse. Its evolution was followed across vertebrates, invertebrates, and was traced to unicellular organisms, thus enabling following evolutionary additions and losses of genes or domains. The N-terminal and C-terminal DCX domains have undergone sub-specialization and divergence. Developmental *in situ *hybridization data for nine genes was generated. In addition, a novel co-expression analysis for most human and mouse DCX superfamily-genes was performed using high-throughput expression data extracted from Unigene. We performed an in-depth study of a complete gene superfamily using several complimentary methods.

**Conclusion:**

This study reveals the existence and conservation of multiple members of the DCX superfamily in different species. Sequence analysis combined with expression analysis is likely to be a useful tool to predict correlations between human disease and mouse models. The sub-specialization of some members due to restricted expression patterns and sequence divergence may explain the successful addition of genes to this family throughout evolution.

## Background

Mutations in the X-linked gene *doublecortin (DCX) *result in lissencephaly or subcortical band heterotopia (SBH) [1,2] (see review in [3]). Lissencephaly is a severe brain malformation characterized by absent (agyria) or decreased (pachygyria) convolutions accompanied by thickening of the cortex [4]. SBH is a related disorder in which there are bilateral bands of gray matter interposed in the white matter between the cortex and the lateral ventricles [5]. In general, lissencephaly and SBH are neuronal migration disorders. SBH is very common among females with mutations in *DCX *[1,2]. DCX [6-9] and its closely related gene product DCLK (doublecortin-like kinase) [10,11] are classical microtubule (MT) associated proteins (MAPs), and contain two evolutionary conserved tubulin binding repeat sequences. We have initially defined the evolutionary conservation based on a limited number of proteins [12], following the expansion in available sequences, but the recent explosion of available sequence data, strongly warrants revisiting of this issue of the evolution of the DCX protein family. Interestingly, most missense mutations that are found in lissencephaly patients cluster in the defined DCX (doublecortin) domain [12,13]. These tandem repeat protein motifs are 11 kDa and are located in the N-terminal part of the 30 kDa DCX protein. The structure of the DCX domain markedly differs from that of classical MAPs adopting a globular structure with a ubiquitin-like fold [14]. Cryo-electron microscopy studies revealed that DCX binds to MTs at a novel site, located in between the protofilaments [15]. This binding site can explain why MTs are stabilized by DCX. Two additional genes of this superfamily are implicated in human diseases. RP1 is the protein product of retinitis pigmentosa -1 (*RP1) *[16,17]. Mutations in this gene result in progressive blindness, not only in humans, but also in a mouse model [18]. *DCDC2 *has been implicated in dyslexia [19,20]. Reduction of *Dcdc2 *in the developing cortex using *in utero *electroporation inhibited neuronal migration [19].

The notion that DCX domain proteins may function in a redundant way during cortical development was raised when *Dcx *null mice exhibited a very mild cortical phenotype [21], whereas acute knockdown of *Dcx *using RNAi resulted in a doublecortex phenotype [22]. Recent experimental evidence indicated that DCLK was redundant with DCX. *Dclk *-/- mice also exhibited a rather mild cortical phenotype, but *Dcx/Dclk *double mutant mice displayed severe cortical defects [23,24]. Recently, an additional member of this protein family, doublecortin kinase 2 (or DCLK2), has been described, and found to posses MT binding activities [25]. Nevertheless, *Dcx *and *Dclk *are but two genes among eleven DCX domain proteins in the mouse genome [26]. Thus, the quest for how DCX domain-containing proteins exert their multiple functions during cortical development is still open [27]. Our previous study has demonstrated common and unique activities in regard to protein interactions, MAP activity, and the subcellular localization of nine of the mouse DCX domain superfamily members [26]. Two unifying functional aspects were detected; all proteins affected MT polymerization, and all interacted with the JNK scaffold proteins, JIP1 and JIP2. Thus, the DCX superfamily of proteins is likely to mediate signal transduction pathways. Proteins with a tandem DCX domain stabilized MTs in transfected cells. Some of the transfected proteins exhibited localization to actin-rich subcellular structures, and in addition most of the DCX domain proteins exhibited a nuclear localization. A distinct set of proteins interacted with the scaffold protein neurabin 2, which binds to PP1 [28] as well as to actin [29]. Positive interactions were observed with DCX, DCLK, and DCLK2, closely related family members, but not with other DCX-domain containing gene products.

In the current study, we studied the evolution of the DCX superfamily from unicellular organisms to humans. It was possible to demonstrate emergence and disappearance of specific genes or individual domains. The N-terminal and C-terminal DCX domains have undergone sub-specialization and divergence. In addition, the expression pattern of gene members was studied using database mining and *in situ *hybridization tools. The sub-specialization of some members due to restricted expression patterns and sequence divergence may explain the successful addition of genes to this family throughout evolution.

## Results

### Identification of proteins with a DCX domain

Human and mouse proteomes were searched for sequences similar to that of the human DCX domain yielding a total of 22 proteins containing one or two DCX repeats (Table 1, the complete sequences used in the present study are found in supplementary Fig. 1).

**Figure 1:**
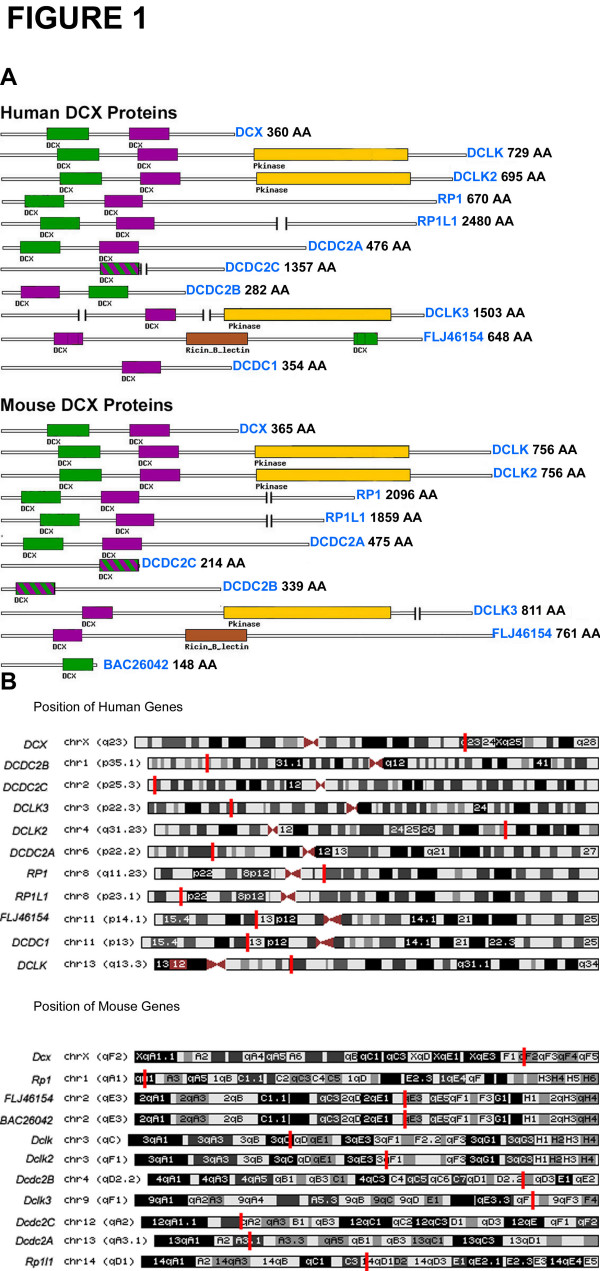
A. Schematic representation of human and mouse proteins containing DCX domains. DCX domains more similar to the N-terminal repeat of DCX were labeled in green, whereas those more similar to the C-terminal repeat were labeled in purple. Protein kinase domains are marked in yellow. The ricin domain is marked in brown. Human proteins are positioned on the top, the mouse proteins are shown below. B. Schematic presentation of the chromosomal location of the human and mouse DCX domain genes using UCSC site [51].

Serine/threonine protein kinase domains were found in three human/mouse proteins (DCLK, DCLK2, and DCLK3), and a ricin domain predicted to bind carbohydrates was found in a human/mouse protein referred to as FLJ46154 [30]. The structure of the human FLJ46154 and DCDC2B proteins differed from other proteins with tandem repeats; they contained a repeat more similar to DCX C-terminal repeat, which appeared in the N-terminal part of this protein, and a second repeat more similar to DCX N-terminal repeat. In the mouse orthologs of these two proteins, only one DCX domain was present. All the mouse genes reside in chromosomal regions (Fig. 1b), which are synthenic to the human orthologs (supplementary Fig. 2). This includes also the location of DCDC1 and BAC26042, however they are not true orthologs since the sequence similarity is very low (52%, among 46 out of 86 amino acids) only in the DCX domain, and the phylogenetic and evolutionary analysis, described below, indicate that they are different. BAC26042 is also unique in its close physical proximity with FLJ46154, the distance between these two genes being only 2 kb, suggesting they may share common regulatory elements.

**Figure 2:**
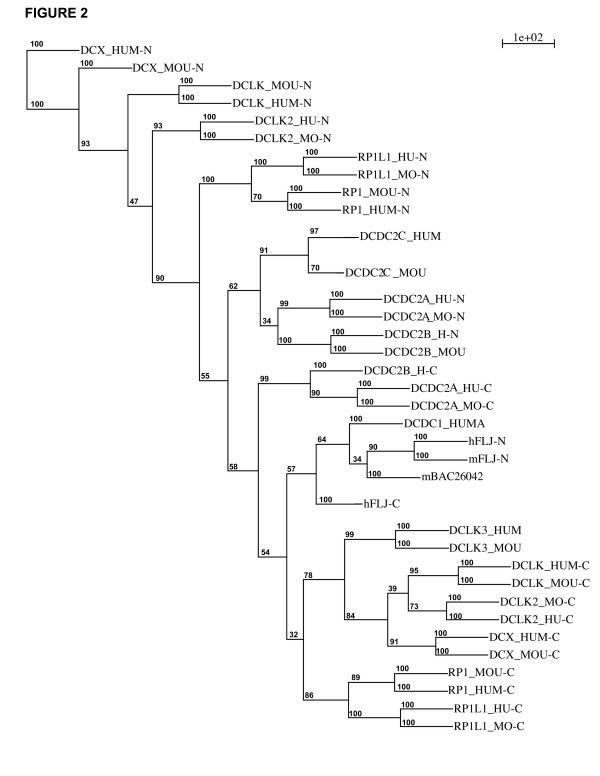
Maximum Likelihood (ML) phylogenetic tree including DCX domain proteins from human and mouse, bootstrap values are indicated.

This study is focused on the DCX domains, and does not cover the full-length proteins. Phylogenetic analysis was conducted for the individual DCX domains, separating the N- and C-terminal parts (Fig. 2). Several interesting features emerged from the human and mouse DCX domain phylogenetic analysis. The majority of human genes had a mouse ortholog. Two genes do not obey this rule as they do not have unambiguous orthologs (human DCDC1 and mouse BAC26042). Furthermore, in most instances the N-terminally located DCX domains were more similar to other N-terminal domains than to the C-terminal domains of the same protein. The two exceptions were already mentioned; human DCDC2B and FLJ46154. Sequence analysis combining BLAT [31] and phylogenetic analysis identified the orthologous relationships listed in Table 1.

**Figure 3:**
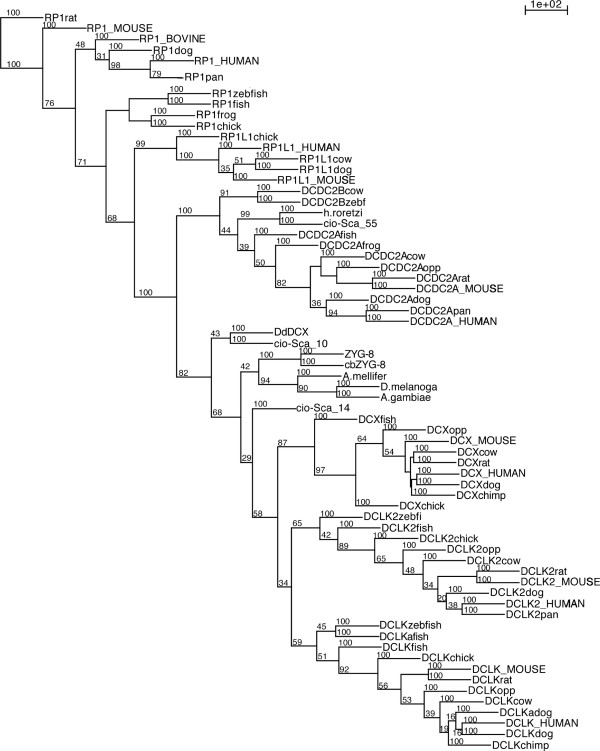
ML tree of the tandem DCX domain proteins from different species. Bootstrap values are indicated.

Next, we extended the sequence analysis by including several additional non-mammalian genomes. Initially, the analysis encompassed proteins found in the conserved domain database CDD [32]. Subsequently, these searches were broadened with extensive BLAST, TBLASTN, and BLAT searches. Using BLAT search [31] sequences from opossum, rat, and rhesus monkey were added. *Ciona *sequences were added using TBLATN analysis against the genomic data, and only those sequences corresponding to ESTs were included. Hence, the present phylogenetic analysis included DCX-motif-containing proteins from human, chimpanzee, mouse, cow, dog, chicken, fish, worms, insects, frogs, fungi, and sea squirts (multiple alignments are provided in supplementary Fig. 3). The analysis of the tandem DCX domain proteins (67 proteins) resulted in an unrooted tree with bootstrap values shown in Fig. 3.

Four groups of proteins are easily categorized within the tandem DCX domain tree, which contains 67 proteins. From top to bottom, the group of RP1 and RP1L1 include orthologs from the frog *Xenopus laevis*, fish (zebrafish *Danio rerio*, and pufferfish *Tetraodon nigrovidis*), chicken, cow, dog, mouse, rat, chimpanzee, and human. The second group includes proteins similar to DCDC2A (previously known as DCDC2, name approved by the HUGO Gene Nomenclature Committee) from mammals, including opossum (a marsupial), as well as chicken, fish, frog, and simpler organisms such as the ascidian *Halocynthia roretzi*, and the sea squirt *Ciona intestinalis*. The third group of proteins is devoid of mammalian proteins, but contains proteins from the social amoebae *Dictyostelium discoideum*, and one protein from *Ciona intestinalis*. Similar proteins were identified in the fruit fly, *Drosophila melanogaster*, the malaria mosquito, *Anopheles gambiae*, and the honey bee, *Apis mellifera*. Furthermore, two similar proteins from the worms *Caenorhabditis elegans *(ZYG-8), and *Caenorhabditis briggsae *are detected in this group. The fourth group of proteins includes those most similar to DCX, DCLK, and DCLK2. This group included mammalian, chicken, fish proteins, and one protein from and *Ciona intestinalis*. This analysis of proteins with two domains, was followed by an analysis for the N- and C- terminal domain proteins (supplementary Figs. 4-5). One hundred and seven proteins were analyzed in the N-group, and one hundred and one proteins in the C-group, suggesting that there are slightly more proteins similar to the N-terminal part of DCX. The general subdivision into the four groups was preserved. Inspection of the proteins composing the N-terminal phylogenetic tree detected that additional proteins were added mainly to the third group containing the *Dictyostelium discoideum *protein (including 8 members). Proteins from flies and worms were also added to this group. The fruit fly genome contains five DCX proteins, four of which are single repeats. Furthermore, several mammalian proteins were added to this group as well. This group was increased to contain 26 members in the N-group and 19 members in the C-group. This group included a protein from the unicellular organism *Plasmodium falciparum*, the malaria parasite.

Inspection of the proteins composing the C-terminal phylogenetic tree detected a group containing all the DCLK3 proteins. It should be noted that this group as a whole is quite distinct from DCX, DCLK, and DCLK2. Proteins in this group contain a single DCX domain from mammals (human, chimpanzee, cow, rat, and opossum), but also from fruit flies, honeybees, and malaria mosquitoes. An exception is the ciona protein demarking this group (Sca_10), which has a tandem repeat. One of the groups contain both DCDC2A and DCDC2B proteins, and yet an additional group contains several more DCDC2B proteins, suggesting probably less evolutionary conserved sequences in the C-terminal domains of this subset of proteins.

During the analysis of the DCX domain proteins, the presence of tandem or single DCX-domains was noted in corresponding orthologs. The simplest way to explain these differences may be through loss of intergenic sequences. The analysis of exon-intron boundaries included all the mammalian species and chicken since it is a non-mammal vertebrate, close enough to mammal to make comparison possible (Table 2). In general, the location of the intron-exon boundaries is highly conserved. In some cases the presence of an additional exon, does not change the length of amino acids that are part of the DCX domains. Such is the case with DCDC2C; most species contain one exon, whereas the cow ortholog the corresponding amino acid sequence is divided into two exons. However, in most cases, the lack of an exon implies a reduction in the amino acid information. For example, FLJ46154 contains in most species three exons, whereas in mouse and in the corresponding sequence in rat only two. Consequently, in mouse and rat only a single DCX domain was identified in the region corresponding to the human FLJ46154 DCX domains. This analysis also allows identifying key time points in the evolution of the DCX-domain proteins. The common vertebrate ancestor of mammals and birds is now believed to reach back 310 million years, marsupials split from the main (placental) group about 180 million years ago, and humans and rodents split off from their evolutionary family tree about 87 million years ago. The above analysis revealed that it is likely that BAC26042 was lost during evolution (in mouse two exons exist, while rat and rhesus monkey harbour only one exon). This analysis has been complicated due to a predicted sequence in rat (XM_230359) that is a fused sequence containing both FLJ46154 and BAC26042. However, we have experimental evidence that do not support the existence of this fused sequence. Antibodies we generated against the mouse FLJ46154 protein recognize a protein of the predicted size for FLJ46154 in mouse brain extract (supplementary figure 6). Thus, we have conducted our analysis based on the human data, which is derived from mRNA and EST data, and the mouse data that is based on EST data, supported by our experimental data. DCLK3 was generated after the mammals and birds split. BAC26042, FLJ46154, and DCDC2C were generated after the marsupials split from the main placental group. DCDC1 was generated after the humans and rodent split. According to this analysis the most conserved genes in this superfamily are DCX, DCLK, and DCDC2A.

**Figure 4:**
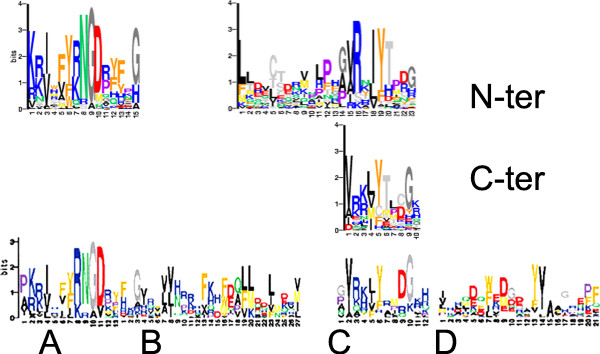
Sequence logos of the N-terminal and C-terminal DCX motifs. Multiple alignments of the motifs from the DCX motifs are shown as sequence logos. The height of each amino acid represents bits of information and is proportional to its conservation at that position (y-axis), after the sequences have been weighted and frequencies adjusted by the expected amino acid frequency. Below the logos is the numbering of amino acids within the internal A-D subdomains. This SeqLogo represents the Lawrence Gibbs sampler motif-finding algorithm.

Following analysis of the two groups including N- and C- terminal domains, analysis for all the DCX proteins was conducted (data not shown). As previously observed for the human and mouse proteins (Fig. 2), the N- and C- terminal domains were more similar to each other than to the corresponding repeat within the same protein. This result suggested that the DCX-domain duplications were ancient, and probably these two repeats have differed in their functions. Subspecialization of the N-terminal and C-terminal DCX motifs can be visualized at the level of logo sequences. Previously, four conserved blocks (A-D) within the DCX motif were identified [12], these conserved blocks are shown in the bottom of Fig. 4. When the N-terminal region was analyzed separately from the C-terminal region, it was obvious that the A and portions of B- and C- subdomains specify the N-terminus, while a portion of the C- subdomain specifies the C-terminus (Fig. 4). This result was obtained using the Lawrence Gibbs sampler motif-finding algorithm. Similar results were obtained with the Smith's MOTIF motif-finding algorithm (data not shown). This analysis indicates that although the tandem domains share a short sequence of similar amino acids, the N-terminal domain has a unique very conserved block of amino acids.

### Expression analysis by *in situ *hybridization

Taken into consideration the similarities among the different DCX-domain paralogs, and their common functions in relation to signal transduction and microtubule regulation [26], it is important to establish when and where these genes are expressed. This will help in delineating their potential function. For example, the distinction whether a specific gene is expressed in proliferating, migrating, or differentiating cells is critical when trying to figure out gene function. Additionally, coexpression in a particular tissue may indicate that paralogs could cooperate or be redundant.

**Figure 5:**
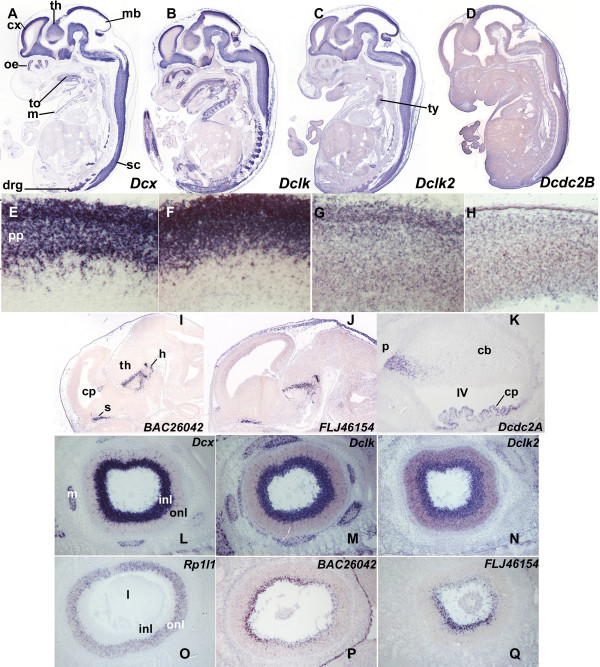
*In situ *hybridization patterns of genes containing the DCX protein domain. Blue stain denotes expression of the gene whose name is indicated in each panel. For details see Text. (A-D) Sagittal sections of whole E14.5 embryos. (E-H) Expression in frontal cortex. Note a few cells expressing in the ventricular zone. (I-K) Striking localized expression of *BAC26042 *(I), *FLJ46154 *(J), and *Dcdc2A *(K) in the E14.5 brain. In (I) "t" becomes "th" for thalamus. (L-Q) Expression patterns in the E14.5 eye. Abbreviations: cerebellum (cb), choroids plexus (cp), cortex (cx), dorsal root ganglia (drg), hypothalamus (h), inner neuroblastic layer (inl), midbrain (mb), muscles (m), olfactory epithelium (oe), outer neuroblastic layer (onl), pons (p), preplate (pp), septum (s), spinal cord (sc), thalamus (th), thymus (ty). tongue (t), ventricular zone (VZ).

Our analysis was carried out by *in situ *hybridization at E14.5, a stage at which many differentiated cell types characteristic of an adult organism have formed yet at same time such mid-gestation embryonic tissues still contain progenitor cells. This analysis was performed with the goal to generate an expression profile "snapshot". With the exception of the ubiquitously expressed *Dcdc2B *(Fig. 5D), expression patterns of genes encoding DCX-repeat-containing proteins are to a greater or lesser extent regional. *Dcx*, *Dclk *and *Dclk2 *are expressed in the central and peripheral nervous system including the brain, spinal cord, cranial and dorsal root ganglia and in the parasympathetic ganglia (Fig. 5A–C). A high power view (Fig. 5E–H) shows that in the developing neocortex *Dcx *and *Dclk *transcripts are much more abundant in the preplate, but individual cells expressing the *Dcx *and *Dclk *genes can be detected in the ventricular zone. Both *Dclk2 *and *Dcdc2B *are expressed in the developing neocortex, largely uniform and at low levels, but more pronounced in the ventricular zone than *Dcx *and *Dclk*. Outside the nervous system, prominent sites of *Dcx *and *Dclk *expression are the skeletal muscles, tongue muscles and individual cells of the olfactory epithelium (Fig. 5A,B). The latter tissue also expresses *Dclk2 *(Fig. 5C).

*BAC26042*, *FLJ46154 *and *Dcdc2A *exhibit highly regional expression patterns, which in the brain appear to be similar for *BAC26042 *and *FLJ46154 *(Fig. 5I–K). Fig. 5I and 5J show sagittal sections through the forebrain with *BAC26042 *and *FLJ46154 *transcripts present in the septum, various cell groups of the ventral thalamus, and in the posterior hypothalamus. Other sites of expression are a group of neurons at the base of the olfactory bulb (Fig. 5I,J), the pretectal area, the facial nucleus, and scattered neurons in the ventral and dorsal parts of the spinal cord (data not shown). *Dcdc2A *expression in the CNS is restricted to a group of scattered neurons in the lateral most part of the developing cerebellum (Fig. 5K). *BAC26042 *and *Dcdc2A *are expressed in the choroid plexi (Fig. 5I,K).

The majority of DCX-repeat encoding genes are expressed in the developing retina. Three types of patterns emerge: *Dcx*, *Dclk*, *Dclk2 *transcripts are strongly expressed in the postmitotic inner neuroblastic layer (Fig. 5L–N), whereas *BAC26042 *and *FLJ46154 *are also expressed in this layer, but in a more restricted fashion near and at its surface (Fig. 5P,Q). Finally *Rp1l1 *transcripts are found in the outer neuroblastic layer that contains proliferating cells (Fig. 5O). Radially arranged *Dcx, Dclk or Dclk2*-expressing cells are detected in the outer neuroblastic layer which is reminiscent of the situation seen in the ventricular zone of the neocortex (Fig. 5E–G).

In addition, lung and kidney express *Dcx*, *Dclk *and *Dcdc2A*. *Dclk2 *transcripts are also found in the developing ovary and weak expression is also seen throughout the kidney (data not shown).

Our analysis included most of the 11 genes listed in Table 1, the exceptions being *Dclk3*, and *Dcdc2C *for which we could not yet identify suitable templates. *Rp1 *was also examined but it is not expressed at E14.5, except expression noted in some midline cells of the spinal cord (data not shown). To summarize our studies, we found that tissues destined to respond to electrical stimuli – central and peripheral nervous systems and skeletal muscles – represent the most striking sites of expression of DCX-repeat encoding genes. Outside these tissues, expression is mostly low and usually not regional, the exceptions being kidney and lung.

### Expression analysis in human and mouse

**Figure 6:**
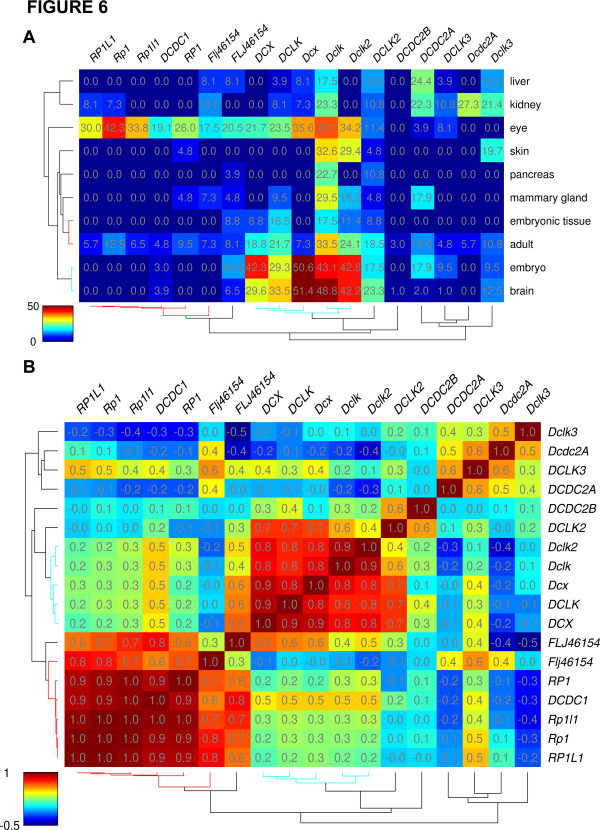
A) Clustered Unigene [33] gene-tissue expression data. B) Gene-gene correlations based on Unigene expression data.

The relevance of functional genomics approaches using mouse models for studying human diseases obviously depends on the similarity of gene expression in the two species. Thus, we compared the expression of the human members of the DCX gene superfamily investigated in this study with their mouse orthologs. For this purpose, we used the Unigene database of expression data website. Tissue-dependent expression profiles for both human and murine DCX repeat-containing proteins were generated from the EST count provided by UNIGENE [33]. Since the mouse-human comparison was a key feature, the analysis was limited to tissues with a high total number of EST counts that were common to both organisms. We analyzed data for ten different human genes, and eight mouse genes. For two human genes there were no corresponding expression data in mouse: *DCDC2B*, which has a mouse ortholog that is not listed in UNIGENE, and *DCDC1*, which does not have a mouse ortholog. The clustered expression data resulting from this analysis is shown Fig. 6A and a gene-gene correlation based on this information is shown in Fig. 6B.

We tested the significance of the correlation by random permuation analysis. The correlations were re-calculated 1000 times after rescambling for each gene independently all tissues at random. We found that all high correlation (>0.5) were significant (p < 0.01). Two clusters revealing very high correlation were observed. The largest group included human *RP1 *and *RP1L1*, and their murine orthologs. In addition, *DCDC1*, which so far had been reported to be expressed mainly in testis, and embryonic brain [34], was included in this group. This group is characterized by high levels of expression in the eye, which is common amongst most DCX proteins, and has been noted in our *in situ *analysis. In addition to expression in the eye, these genes are expressed at lower levels only in a few other tissues. In this group there is no clear distinction in the gene-gene correlation in the expression in mouse and human. The correlation between the different members of this group is >0.9 in all cases. Both the human and the mouse *FLJ46154 *are related to this group, however the correlation between the human and mouse *FLJ46154 *is low (0.3). The protein-products of these two genes have also diverged, with a loss of a DCX domain in the mouse protein. Thus, it may be possible that there has been less conservation in the regulatory regions of these genes as well.

The second group exhibiting high gene-gene correlations includes the murine genes *Dcx, Dclk*, and *Dclk2*, and their human orthologs. Human *DCLK2 *exhibited somewhat lower correlations with its mouse ortholog (0.4) than the other genes in this group. This may stem from its general overall lower expression levels (Fig. 6A). Our *in situ *data also indicated a high similarity in the co-expression of *Dcx, Dclk*, and *Dclk2*. Furthermore, our functional analysis [26] indicated that this group shares more properties and only they interact with the scaffold protein neurabin 2. A third group of genes with lower levels of correlation include *DCDC2A, DCLK3*, *Dcdc2A*, and *Dclk3*. In this group the correlation between the corresponding orthologs does not exceed 0.5. It should be noted that there are some additional high correlations between different genes, for example; *DCLK3 *and *Flj46154*, or *FLJ46154 *with *DCX, DCLK*, and *Dcx*.

## Discussion

### Protein architecture

#### Number of doublecortin repeats

The DCX domain family of proteins consists of multiple members in the animal kingdom from the unicellular organisms *Plasmodium *and *Dictyostelium *(in the non-aggregated part of its life cycle) to human. Although, *Plasmodium *and *Dictyostelium *do not have a nervous system, motility is one of their important characteristics. The shift from a single to a tandem DCX repeat (or *vice versa*) is a rather simple mode to modifying protein activity from binding to tubulin and assisting MT polymerization (one repeat) to enabling it to promote MT bundling (two repeats). We have demonstrated experimental evidence for this view for DCX [12], and for eight additional members of the mouse DCX domain proteins [26]. The duplication of a single DCX motif was an ancient event because of the existence of a tandem repeat already in *Dictyostelium*, and since the similarity within the N-terminal domains is generally higher than to the C-terminal domains of the same protein. We were able to document the loss of a domain, by the loss of an exon ("domain death"). Interestingly, the group of proteins encoding a single repeat is expanded in nematodes. These single repeat proteins are usually similar to the same extent to the groups of the N- and C- terminal repeats. Our previous analysis [12], structural analysis of DCX [14,15,35], and our current analysis reveals differences between the N-terminal and the C-terminal domains. Our analysis detected a short stretch of conserved amino acids common to both N-terminal and C-terminal repeats, however the sequences diverged and the N-terminal domain has a unique block of conserved amino acids. Close inspection of the phylogenetic trees revealed higher evolutionary conservation among proteins in the N-terminal group than those in the C-terminal group. Our analysis detected that both the N-terminal and C-terminal domains of DCX promote assembly of MTs [12], however the N-terminal portion has higher activity than the C-terminal one. In addition, other proteins containing a single or tandem DCX domain were capable of promoting MT assembly [26]. Among the studied mouse proteins FLJ46154 exhibited the highest activity in promoting MT assembly [26]. This protein contains a single DCX domain, which is more similar to the C-terminal domain of DCX. Future studies directed at the structural analysis of the different DCX domains combined with the sequence analysis conducted here may provide the basis for elucidating these functional variations. Another study indicated that the N-terminal domain of DCX binds only to assembled MTs, whereas the C-terminal domain binds both to assembled MTs and to unpolymerized tubulin [14]. Therefore, the conserved amino acid block common to both repeats may be important in directing the binding to assembled MTs.

#### Additional domains

A significant portion of the DCX-domain containing proteins has a kinase domain attached to them. This group includes DCLK, and DCLK2, in which a tandem DCX repeat is detected, and DCLK3, which contains a single repeat more similar to the C-terminal one. Orthologs of these proteins are also found also in invertebrates including nematodes. The *C. elegans *ortholog ZYG-8 has been studied extensively [36]. In nematodes, ZYG-8 was found to be important for assembly of astral microtubules. The mutant ZYG-8 phenotype was observed with several different alleles including mutations in the DCX domain, and the kinase domain, therefore suggesting a role for kinase activity in regulating MT assembly. So far, the endogenous substrates of these kinases, besides DCLK which undergoes autophosphorylation [37], are unknown.

Addition (or reduction) of the number of domains is not always conserved in evolution. In close relationship with DCLK3, which contains a kinase domain, a branch including proteins with a different domain is found. This branch includes the *Drosophila melanogaster *protein (EMAL_DROME), which contains an additional HELP domain, and WD repeats, which can be detected in the closely related *Anopheles gambiae, and Apis mellifera *proteins. The HELP (Hydrophobic ELP) motif is found in EMAP and EMAP-like proteins (ELPs), and has been found to mediate binding to MTs *in vitro *[38,39]. Therefore, the combinatorial possibilities of modifying or changing functional activities will depend upon specific amino acid substitutions within the DCX domains, and addition of other functional domains.

#### Additional ways to add on functions

On the level of the whole organism, one of the best ways to modify protein activity is to vary the expression pattern. Indeed, even in the case of proteins with similar sequence and similar expression pattern such as in the case of DCX, DCLK, and DCLK2 some notable differences in expression pattern were noted. For example, the expression of DCLK2 in the ventricular zone of the cerebral cortex is much more obvious than that of DCLK, suggesting that the former may have a pronounced role in regulation of proliferation of neurogenic progenitors. In fact, the involvement of DCLK in regulation of mitosis has been demonstrated recently [40]. DCLK regulated the formation of bipolar mitotic spindles and the proper transition from prometaphase to metaphase during mitosis in HEK293 cells and neuronal progenitors. In cultured cortical neural progenitors, DCLK RNAi disrupted the structure of mitotic spindles and the progression of M phase, causing an increase of cell-cycle exit index and an ectopic commitment to a neuronal fate [40]. As mentioned above, the *C. elegans *ortholog of DCLK, ZYG-8 regulates assembly of astral microtubules [36]. Taken together, these findings suggest that regulation of the MT-based mitotic spindle may represent a conserved function among DCX-domain proteins.

Two genes FLJ46154, and BAC26042, exhibited very similar expression patterns in the mouse. In the mouse genome, the distance between these two genes is only 2 kb, suggesting that they may share common regulatory sequences. Perhaps due to such a highly similar expression pattern, the loss of BAC26042 in other mammalian species is not surprising. Partial copies of this gene (containing only one exon instead of two) exist in rat and in the rhesus monkey. A proposed unified nomenclature for the doublecortin superfamily based on correspondence with the mouse gene nomenclature committee is presented in supplementary figure 7.

#### Expression patterns and phylogenetic correlations

One of the most exciting outcomes of the present analysis is that in some cases we observed a high correlation between orthologs and paralogs, and their expression profiles. This was most striking in case of the human and mouse *RP1*, and *RP1L1*. A possible hypothesis, based on these results is that a high correlation in the expression of the human and mouse genes may be indicative of a high probability that a mouse mutant would model the human disease (or *vice versa*). If we review this hypothesis within the limited genetic data available, we see that this hypothesis is supported. *Rp1*-/- mice closely reflect very well the progressive blindness observed in human patients [16-18,41,42]. Furthermore, in these mice the JNK pathway was affected [43], and our functional analysis describe a possible connection between RP1 and the JNK pathway [26]. An unexpected member to this group is DCDC1. The gene-to-gene function correlations are also very high in case of the human and mouse *DCX, DCLK*, and *DCLK2 *genes. They are found on adjacent branches in the phylogenetic analysis. These genes are highly expressed in the central nervous system not only in human and mouse, but also in additional animals as chicken (DCX, DCLK [44], and DCLK2 (unigene data for Dcx Gga.2608, Dclk2 Gga.16742), cow (dclk and dclk2; Bt.34533, Bt.55548), rat (dcx Rn.121471, dclk Rn.155540, dclk2 Rn.23327), and dog (dcx Cfa.19843, dclk Cfa.9988). Our analysis detected expression of some of these genes in several additional tissues such as muscle, heart, and kidney, sites which have not been previously described. Although *Dcx-/- *mice have minor neuronal abnormalities [21,45], they do not adequately recapitulate the human disease. This is due to gene redundancy because the double mutant mice *Dcx-/- *and *Dclk-/- *exhibited perinatal lethality accompanied with multiple brain abnormalities [23,24]. However, using *in utero *electroporation in the rat and mouse embryo, reduction in the expression of *Dcx *resulted in apparent inhibition of migration of neurons in the cerebral cortex [22,46]. In the rat, it was possible to observe heterotropic positioning of neurons in postnatal rat brains similar to SBH in human [22]. In contrast to the cases discussed, there are several genes for which the orthologs exhibit low gene-gene correlation; the lowest are *DCLK3 *and *FLJ46154 *(0.3), *DCLK2 *(0.4), and *DCDC2A *(0.5). Among these genes variability in sequence analysis was noted as well. With future expansion of available information in databases, this analysis will be extended to include many more tissues, addition developmental stages, and additional species. We suggest that this analysis may be useful in predicting possible functional redundancy. Furthermore, it may provide useful clues in which tissues the mouse gene expression is most similar to its human ortholog, thus suggesting the possible relevance of a corresponding mouse model for a human disease.

## Conclusion

In summary, the DCX-domain family of proteins has proven to be a very successful and prolific protein family involved in signal transduction and cytoskeletal regulation. Modifications in regulatory sequences allowing unique expression patterns, addition of new domains, and modifying the amino acid sequence of the DCX domains themselves explains – at least in part- why this superfamily is thriving.

## Methods

### Database homology search and phylogenetic analysis

Our database similarity search initiated with an NCBI blast search using the sequence of mouse doublecortin protein (NP_034155.2). In addition, all proteins with the DCX domain were retrieved from the CDD, (Conserved Domain DB). In a similar fashion, DCX proteins from the EBI InterPro db, which were not detected in CDD were extracted and added. In order not to miss related orthologs, BLAT analysis using UCSC BLAT site [31] was conducted initiating with the human and mouse genes. Definition of orthologs was based on positioning in syntenic chromosomal localizations. In addition, an exhaustive TBLASTN search was conducted to detect DCX-related transcripts in the EST database of *Ciona intestinalis *(sea squirt) [47]. The corresponding cDNAs were mapped to their corresponding genomic localizations.

For our analysis, three datasets were generated, the first dataset for the proteins with 2 DCX motifs (including both the N-term and C-term repeats) composed of 57 proteins from human, mouse, chicken, bovine, ciona, frog, fish, nematodes, chimp and flies.

The second dataset included the proteins with a single DCX motif, which exhibited higher similarity to the mouse doublecortin C-terminal DCX motif, and the C-terminal ones of those with two DCX motifs. The single repeats exhibited which exhibited identical similarity to both the N- and C-terminal repeats of mouse DCX were included in both N-term and C-term groups. The comparison was based on two-way BLAST analysis using blast2seq. This dataset integrated 77 proteins from human, mouse, chicken, ciona, dog, bovine, frog, fish, sea squirt, nematode, ascidian, fruit fly and fungus.

The third dataset was composed of the proteins with a single DCX motif, which was more similar to the mouse doublecortin N-terminal DCX motif, and the N-terminal ones of those with two DCX motifs. This dataset included 85 proteins from the species mentioned above.

Multiple alignment program, Clustalw, was run on each of the datasets and the regions corresponding to the DCX motifs were extracted, resulting in three multiple alignments (data sets are deposited in supplementary data 1).

The Phylip package ver. 3.6 was used to build the ML trees with boot strapping values.

One hundred datasets were generated using the program SEQBOOT from the original data (multiple alignment done by clustalw) for each of the 3 groups of motifs (2-repeats, C-terminal repeats and N-terminal repeats). This was followed by running the program ProML (protein Maximum Likelihood) on each of the datasets in the group, using the JTT model. A consensus tree (from all the 100 trees) was generated using the program CONSENSE.

The trees were drawn using NJPLOT program or TreeView [48]. All sequences of the three original datasets are available in supplementary Fig. 1.

Separate datasets were generated for the human and mouse proteins, the first dataset for the proteins that have two DCX motifs (N-terminal and C-terminal) composed of 13 proteins. The second dataset for the proteins with a single DCX motif more similar to the mouse doublecortin C-term DCX motif, and the C-terminal ones of those with two DCX motifs. This dataset included 20 proteins. A third dataset included the proteins with a single DCX motif more similar to the mouse doublecortin N-term DCX motif, and the N-terminal ones of those with two DCX motifs. This dataset has 21 proteins. These datasets were subject to all the programs as mentioned above, clustalw, seqboot, proml, consense and njplot. CONSENSE and NJPLOT were used to draw the human-mouse phylogenetic trees.

Multiple alignment was done by Block Maker web server at the Fred Hutchinson Cancer Research Center in Seattle, Washington, USA [49]. The Logoshown represents the Lawrence Gibbs sampler motif-finding algorithm, similar (but not identical) results were obtained using Smith's MOTIF motif-finding algorithm.

### Extraction of public large-scale expression data and cluster analysis

EST counts from different human and murine tissue samples were extracted for 10 human and 8 mouse genes from the UNIGENE website [33]. We discarded all human samples with less than 150,000 total EST counts and retained only the murine samples that could be matched to these samples. The murine 'late gestation' sample was matched to the human 'embryo'. Expression value are given as "counts per million of total ESTs". We applied a scaling transformation '*x *→ *t*·arcsinh(*x*/*t*)' to the raw expression values *x *to scale down (logarithmically) values above the threshold *t *= 10.

In order to identify similarly expressed genes the combined set of expression data was clustered using Pearson correlations over all tissues as similarity measures and single linkage for the generation of the dendrograms. Similarly, the tissues were clustered based on similar expression across the genes. Alternative clustering methods were tested and yielded similar results. We used the standard Matlab clustering algorithm.

### *In situ *hybridization

The antisense RNA templates for *in situ *hybridization were generated by *in vitro *transcription using PCR products (using the appropriate combinations of T7, T3, and SP6 primers) from the corresponding genes, which were cloned in BSIIKS+ or pGEMT. The sizes of the probes ranged between 0.6 – 3 kb. The sequences of the templates can be requested from the author. In several cases more than one probe per gene was tested. Hybridizations and data collection were done as described [50].

## Authors' contributions

OR, SB, GE planned the experiments, collected and analyzed the data, and wrote the manuscript. FMC, PB, TL, AK, TS, NB, conducted experiments, analyzed the data, and participated in manuscript writing.

## Supplementary Material

Additional File 1 Supplementary Fig. 1: Detailed list and sequences of all the DCX proteins used in this study. The list contains the set of proteins with two domains. In addition, the N-terminal domains including proteins with single domains more similar to the N-terminal domain of DCX, and the C-terminal domains including proteins with single domains more similar to the C-terminal domain of DCX are presented in this set. Click here for file

Additional File 2 Supplementary Fig. 2: Synthenic positions of mouse and human DCX domain proteins. Click here for file

Additional File 3 Supplementary Fig. 3: CLUSTAL W (1.83) multiple sequence alignment of the DCX proteins with tandem domains, N-terminal domains and C-terminal domains. Click here for file

Additional File 4 Supplementary Fig. 4: ML tree of the N-terminal DCX domain proteins from different species. Bootstrap values are indicated. Click here for file

Additional File 5 Supplementary Fig. 5: ML tree of the C-terminal DCX domain proteins from different species. Bootstrap values are indicated. Click here for file

Additional File 6 Supplementary Fig. 6: Western blot analysis using anti-FLJ46154 antibodies. The specificity of the antibodies was verified using cells transfected with Flag-FLJ46154 (A) compared with the preimmune serum. (B) Anti-FLJ46154 antibodies recognize a protein of approximate size 78 kDa in mouse brain extract. Click here for file

Additional File 7 Supplementary Fig. 7: Proposed nomenclature for the doublecortin superfamily. Click here for file

## Figures and Tables

**Table 1:** Sequence relationships between human and mouse proteins containing one or two DCX domains.

Symbol of the *Homo sapiens *proteins	Accession	Chromosome	# of DCX repeats	Symbol of the *Mus musculus *proteins	Accession	Chromosome	# of DCX	% similarity of orthologs
DCX	O43602	X	2	DCX	NP_034155	X	2	358/360 99%
DCLK	NP_004725	13	2	DCLK	NP_064362	3	2	681/702 97%
DCLK2	AAH32726	4	2	DCLK2	NP_081815	3	2	679/691 98%
RP1	AAD44198	8	2	RP1	NP_035413	1	2	544/675 80%
RP1L1	NP_849188	8	2	RP1L1	Q8CGM2	14	2	812/1440 56%
DCDC2A	AAF23612	6	2	DCDC2A	AAH45136	13	2	290/307 94%
DCDC2B	XP_497666	1	1C+1N	DCDC2B	XP_357395	4	1NC	207/303 68%
DCDC2C	XP_371476	2	1NC	DCDC2C	XP_917846	12	1NC	107/145 73%
DCLK3	BAB21856	3	1C	DCLK3	NP_766516	9	1C	411/633 64%
FLJ46154	NP_940864	11	1C+1N	FLJ46154	XP_489892	2	1C	494/649 76%
DCDC1	P59894	11	1C	None				
None	-	-	-	BAC26042	BAC26042	2	1N	

**Table 2:** Summary of the number of exons in DCX domains using BLAT [31]. The species analyzed are human (hum), chimpanzee (pan), rhesus monkey (rhe), dog, cow, mouse (mou), rat, opossum (opp), chicken (chi).

Gene	hum	pan	rhe	Dog	cow	mou	Rat	Opp	chi
*Dcx*	3e	3e	3e	3e	3e	3e	3e	3e	3e
*Dclk*	3e	3e	3e	3e	2e	3e	3e	3e	4e
*Dclk2*	4e	4e	4e	3e	4e	3e	3e	3e	4e
*Dclk3*	1e	1e	1e	1e	1e	1e	1e	1e	na
*Rp1*	2e	2e	2e	3e	3e	3e	2e	2e	1e
*Rp1l1*	2e	1e	2e	1e	2e	2e	Na	1e	1e
*Dcdc2a*	5e	5e	5e	4e	5e	5e	5e	4e	5e
*Dcdc2b*	3e	3e	3e	3e	3e	2e	2e	1e	1e
*Dcdc2c*	1e	1e	1e	1e	2e	1e	1e	na	na
*Flj4615*	3e	3e	3e	3e	3e	2e	2e	na	na
*Dcdc1*	1e	1e	1e	1e	1e	na	Na	na	na
*Bac26042*	na	na	1e	Na	na	2e	1e	na	na
