# Supplementary material for: The evolving doublecortin (DCX) superfamily
Source: BMC Genomics. 2006 Jul 26;7:188. doi: 10.1186/1471-2164-7-188 (PMC1550402; doi:10.1186/1471-2164-7-188)
Supplement: Additional File 1 — Supplementary Fig. 1: Detailed list and sequences of all the DCX proteins used in this study. The list contains the set of proteins with two domains. In addition, the N-terminal domains including proteins with single domains more similar to the N-terminal domain of DCX, and the C-terminal domains including proteins with single domains more similar to the C-terminal domain of DCX are presented in this set. [file 1471-2164-7-188-S1.doc]

All DCX Domain Proteins; sequence information

2 domains

| >DCX_MOUSE NP_034155 |
| --- |
| NEKKAKKVRFYRNGDRYFKGIVYAVSSDRFRSFDALLADLTRSLSDNINLPQGVRYIYTIDGSRKIGSMD |
| ELEEGESYVCSSDNFFKKVEYTKNVNPNWSVNVKTSANMKAPQSLASSNSAQARENKDFVRPKLVTIIRS |
| GVKPRKAVRVLLNKKTAHSFEQVLTDITEAIKLETGVVKKLYTLDGKQVTCLHDFFGDDDVFIACGPEKF |
| RYAQDD |
|  |
| >DCLK_MOUSE NP_064362 |
| SEKKAKKVRFYRNGDRYFKGIVYAISPDRFRSFEALLADLTRTLSDNVNLPQGVRTIYTIDGLKKISSLD |
| QLVEGESYVCGSIEPFKKLEYTKNVNPNWSVNVKTTSASRAVSSLATAKGGPSEVRENKDFIRPKLVTII |
| RSGVKPRKAVRILLNKKTAHSFEQVLTDITDAIKLDSGVVKRLYTLDGKQVMCLQDFFGDDDIFIACGPE |
| KFRYQDD |
|  |
| >RP1_MOUSE NP_035413.1 |
| HPVVAKRISFYKSGDPQFGGVRVVVNPRSFKTFDALLDSLS |
| RKVPLPFGVRNISTPRGRHSITRLEELEDGKSYVCSHNKKVLPVDLDKARRRPRPWLSSRSISTHVQLCP |
| ATANMSTMAPGMLRAPRRLVVFRNGDPKNKHVVLLSRRITQSFEAFLQYLTQVMQCPVAKLYATDGRKVP |
| SLQAVILSSGAVVAAGREPFKPGNYD |
|  |
| >DCDC2_MOUS AAH45136.1 |
| SQPVVKSVLVYRNGDPFFAGRRVVIHEKKVSSFDVFLKEVTGGVQAPFGAVRNIYTPRT |
| GHRIRKLDQIESGGNYVAGGPEAFKKLNYLDIGEIKKRPMEAVNTEVKPVIHSRINVSARFRKSLHEPCT |
| IFLIANGDLISPASRLLIPKKALNQWDHVLQMVTEKITLRSGAVHRLYTLEGKLVESGAELENGQFYVAV |
| GRDKFKRLPYSE |
|  |
| >RP1L1_MOUS Q8CGM2 |
| AKKITFLKRGDPQFAGVRLAVHQRTFKTFSSLMDELSQRMPLSFGVRSVTTPRGLHGLSALEQLQDGGCY |
| LCSDRKPPKTSREPGRLQRKSPSAGQAQVFQGGHEAPETSYSWKGPVAPRRLTLVKNGDPRRQQTVVLSH |
| KNTRSLAAFLGKASELLRFPVKQVYTTRGKKVDSLQTLLDGPSVLVCAGNEAFR |
|  |
| >DCLK2_MOUS NP_081815 |
| SEKKAKKARFYRNGDRYFKGLVFAISNDRFRSFDALLIELTRSLSDNVNLPQGVRTIYTIDGSRKVTSLDELLE |
| GESYVCASNEPFRKVDYTKNVNPNWSVNIKGGTTRTLAVASAKSEVKESKDFIKPKLVTVIRSGVKPRKA |
| VRILLNKKTAHSFEQVLTDITEAIKLDSGVVKRLCTLDGKQVTCLQDFFGDDDVFIACGPEKYRYAQDD |
|  |
| >DCX_HUMAN O43602 |
| NEKKAKKVRFYRNGDRYFKGIVYAVSSDRFRSFDALLADLTRSLSDNINLPQGVRYIYTIDGSRKIGSMD |
| ELEEGESYVCSSDNFFKKVEYTKNVNPNWSVNVKTSANMKAPQSLASSNSAQARENKDFVRPKLVTIIRS |
| GVKPRKAVRVLLNKKTAHSFEQVLTDITEAIKLETGVVKKLYTLDGKQVTCLHDFFGDDDVFIACGPEKF |
| RYAQDD |
|  |
| >DCLK_HUMAN NP_004725 |
| SEKKAKKVRFYRNGDRYFKGIVYAISPDRFRSFEALLADLTRTLSDNVNLPQGVRTIYTIDGLKKISSLD |
| QLVEGESYVCGSIEPFKKLEYTKNVNPNWSVNVKTTSASRAVSSLATAKGSPSEVRENKDFIRPKLVTII |
| RSGVKPRKAVRILLNKKTAHSFEQVLTDITDAIKLDSGVVKRLYTLDGKQVMCLQDFFGDDDIFIACGPE |
| KFRYQDD |
|  |
| >DCLK2_HUMA AAH32726 |
| SEKKAKKARFYRNGDRYFKGLVFAISSDRFRSFDALLIELTRSLSDNVNLPQGVRTIYTIDGSRKVTSLDELLE |
| GESYVCASNEPFRKVDYTKNINPNWSVNIKGGTSRALAAASSVKSEVKESKDFIKPKLVTVIRSGVKPRK |
| AVRILLNKKTAHSFEQVLTDITEAIKLDSGVVKRLCTLDGKQVTCLQDFFGDDDVFIACGPEKFRYAQDD |
|  |
| >RP1_HUMAN AAD44198 |
| AKRISFYKSGDPQFGGVRVVVNPRSFKSFDALLDNL |
| SRKVPLPFGVRNISTPRGRHSITRLEELEDGESYLCSHGRKVQPVDLDKARRRPRPWLSSRAISAHSPPH |
| PVAVAAPGMPRPPRSLVVFRNGDPKTRRAVLLSRRVTQSFEAFLQHLTEVMQRPVVKLYATDGRRVPSLQ |
| AVILSSGAVVAAGREPFK |
|  |
| >DCDC2_HUMA AAF23612 |
| KSVLVYRNGDPFYAGRRVVIHEKKVSSFEVFLKEVTGGVQAPFGAVRNIYTPRT |
| GHRIRKLDQIQSGGNYVAGGQEAFKKLNYLDIGEIKKRPMEVVNTEVKPVIHSRINVSARFRKPLQEPCT |
| IFLIANGDLINPASRLLIPRKTLNQWDHVLQMVTEKITLRSGAVHRLYTLEGKLVESGAELENGQFYVAV |
| GRDKFK |
|  |
| >RP1L1_HUMA NP_849188 |
| AKKITFLKRGDPRFAGVRLAVHQRAFKTFSALMDELSQ |
| RVPLSFGVRSVTTPRGLHSLSALEQLEDGGCYLCSDKKPPKTPSGPGRPQERNPTAQQLRDVEGQREAPG |
| TSSSRKSLKTPRRILLIKNMDPRLQQTVVLSHRNTRNLAAFLGKASDLLRFPVKQLYTTSGKKVDSLQAL |
| LHSPSVLVCAGHEAFR |
|  |
| >DCLK2chick XP_420439 |
| SEKKARKARFYRNGDKYFKGLVYAISSDRFRSFDALLAELTRSLSDNVNLPQGVRTIYTIDGSKKLSSLDE |
| LLEGESYVCASNEPYRKVDYTKNVNPNWCVNIRTGSTRSLTSLTSTKSEVKESKDFIKPKLVTVIRSGVKP |
| RKAVRILLNKKTAHSFEQVLTDITEAIKLDSGVVKRLCTLDRKQVTCLQDFFGDDDVFIACGPEKYRYAQDD |
|  |
| >RP1chick XP_426089 |
| AKRICFYKSGDPQFNGIKMVINNRSYKTFDALLDSLSKRVPLPFGVRNISTPKGRHSITCLDDLEDGK |
| SYICSHQRKMKPINLERASRKPLPWQISRPISARRRAVQLAKENEDGFGHRESKITTPKKMLVFKNGDVRLR |
| RTIVLGKKNTQTFEAFLDYMSELMQYPVAKLYTTDGRKVPNLQALILCSGAIVAAGREPFK |
|  |
| >RP1L1chick XP_426222 |
| AKKITFFKSGDPQFGGVKMAINQRSFKSFNALMDDLSHRVPLPFGVRTITTPRGIHCISELDQLEDGG |
| CYLCSDKKYVKPINITTAGHRPGPPRNGRPSSTLRRAAQEGRLDDYSTPFTQHGPRIPKKITLVKNGETSFR |
| RSIILNRRNARSFKTLLDEISEILQFPVKKLFTVDGKKIDSMQALLHCPNVLVCVGREPFK |
|  |
| >DCXchick AAK15319 NP_989666 |
| NEKKAKKVRFYRNGDRYFKGIVYAVSSDRFRSFDALLADLTRSLSDNINLPQGVRYIYTIDGS |
| RKIGSMDELEEGESYVCSSDNFFKKVEYTKNVNPNWSVNVKTSANQKAPQSLASSNSAQAKEN |
| KDFVRPKLVTIIRSGVKPRKAVRVLLNKKTAHSFEQV |
| LTDITEAIKLETGVVKKLYTLDGKQVTCLHDFFGDDDVFIACGPEKFRYAQDD |
|  |
| >DCLKchick XP_417099 |
| SEKKAKKVRFYRNGDRYFKGIVYAISPDRFRSFEALLADLTRTLSDNVNLPQGVRTIYTIDGSKKISSL |
| DQLVEGESYVCGSIEPFKKLEYTKNVNPNWSVNVKTTSTSRSVPSLATAKGGTPDTKENKDFIRPKLVT |
| IIRSGVKPRKAVRILLNKKTAHSFEQVLTDITDAIKLDSGVVKRLYTLDGKQIFLAFRLIAAVDARAFW |
| HTSGKHDMNFIAPPLPVAAGWEVVMVMCLQDFFGDDDIFIACGPEKFRYQDD |
|  |
| >A.gambiae XP_319785 EAA14780 |
| PAKKAKRIRFFRNGDKFYPGSTIPVSVERYRSFDSLTEDLTRLLEDSVTLTGAIRAIYTL |
| EGKKIEKVDDLEDGKCYVCSCNNEGFKRIDYNVSNTNTKNPNRLSRTERNLMLFAACFFFCGRLIRPLSP |
| VKNGGSNGSTPLKEIDSVVHPRIVTLIRNGVKPRKILRLLLNKRNSPTYEHVLTAITQCVKLDTGCVRKV |
| FTVAGVPVQRLAQFFEEDDVFFAYGNERVGNNDFELEAEE |
|  |
| >A.mellifer XP_394386 |
| PTKKAKRVRFFRNGDKFYTGIVMAVTPERYRSFDSLATDLTRALISSVTLPNGVRAIYTMDGKKVQSIND |
| LEDGKCYVVSGQGEIFKKVEYSSTKVRRGSSLSGLPQSPAGTGRQISAIPLCVKAKIITLIRHGTKPRKV |
| VRLLLNKRNAPSLEHALEAITEAVKLDSGAVRKVYTLSGQQVTSLEQFFENDDIFVAYGPEK |
|  |
| >D.melanoga AAM11416 |
| PTRKALRIKFYRNGDRFYPGITIPVSNERYRSFERLFEDLTRLLEENVKIPGAVRTIY |
| NLCGKKITSLDELEDGQSYVCSCNNENFKKVEYNTGSQPLSNLPLSNSRSNSHRLAKCRPSSPLKNGLLA |
| GSSPFPACGGGTGNGSPLIASRLSDRVTVVHPRIVTLIRSGTKSRRIMRLLLNKRNSPSFDHVLTAITQV |
| VRLDTGYVRKVFTLSGIPVVRLSDFFGSDDVFFAYGTERINTAED |
|  |
| >DCDC2frog AAH46665 |
| QQPAVKTVHVFRNGDPFYRGRRMLIHERRVGTFDVFLKDVTGGVQAPFGAVR |
| NIYTPRNGHRVTSLDDLQPGEFYVAGGRENFKKLDYLHIGEIKRKTVDPLSQVKPVSHSRINVSARFRKN |
| VQEPCTVFLVANGDTLNPFIRLLIPRKTLEQWELVLALVTEKVKLRNGAVHRLYTLEGTPIQNGLELENG |
| QFYVAVGREKFKKLPYS |
|  |
| >RP1frog AAH78105 |
| EPASTKRVCFYKSGDPQFNGIKMVVSNRSFKTFDALLDTLSKKVPLPFGVRNISTPRGIHHVTSINELED |
| GKSYICSHRKKIKPINLDRARKKPLLWQSSRPISARRLAVQLAQQNEVVPLQRKNTIVLGSSKKIIIFKN |
| GDLGFKHHFNLNKKTKQSFDSFLDQVAEALQYPVFKLYSSDGRRILSIRALLLSSGTVVAAGRESFIYAN |
| YE |
|  |
| >h.roretzi BAB40784 |
| QPETAKNVHVYVNGDQYYPGRKFVVNRRYICDFDGFLNLVTSGLKPSFGAVRNIYT |
| PNAGHRVNELHEINNNMVIVAGGAERFRKLHYQNITPLPRKPMPPKHSYSTIRPVYHNSKMNVPARWKQL |
| VQGPCSIYVYGNRDINGVAIKMLLSARIMKNWDMVLEEITEKISIRTGQAVRRLYTLDGVLIQGSENLEN |
| GRYYVAVGYERFKRAPYG |
|  |
| >DCDC2fish CAF96719 |
| SQPVVKNIFMFRNGDPYYEARRIVINQKRVSNFETLLREVTGGIQAPFGAVRTIYTPRGGHK |
| VNSMENLKSGEQYVAAGREKFKKLDYLEIGSRRKRMLHPAQVKPPPQNRFIVSARFLKPIKEPCAVFVVA |
| NGDVLNSAVRLLIHQRMLGQFDKILEMITEKMGLRVLGGVRSLYTYDGTQVNDGNQLESGQLYVAVGRER |
| FKKLPYI |
|  |
| >DCLKafish CAG00429 |
| EEKRAKKVRFYRNGDRYFNGIVYAISTDRFRTFDALLADLTRSLSDNVNLPQGVRTIYTLDGTKKIGAID |
| QLVEGDSYVCSSNEAYKKLDYTKNVNPNWSVNVKASPTSSRGPPSLGNTNAVVPENRETKDFIRPKLVTV |
| VRSGVKPRKAVRILLNKKTAHSYEQVLTDITDAIKLDSGVVKKIYTLEGKLVSCLQDFFGDEDVFVACGP |
| EKFRYQDDL |
|  |
| >DCLK2fish CAG02200 |
| SEKKAKKVRFYRNGDRYFKGLVYAVSSDRFRSYDALLMELTRSLSDNLYLPQGVRTIYSVDGSKKIGSMDE |
| LVEGESYVCASNEPYKKLEYTKISIPSWKPGAAPGAAAASRPPTTSTGVSAGATVAPAAVSRDRPESRES |
| RENKDFIKPKLVTVIRSGVKPRKAVRILLNKKTAHSFEQVLADITEAIKLDSGAVKRLYTLDGKQLTCLQ |
| DFFGDDDVFMACGPEKFRYAQDD |
|  |
| >RP1fish CAG10227 |
| RSKRVCFYKSGDAQFSGLRMVINNRTFNTFDALLDSLSRKVPLPFGVRNITTPHGVHAVHTLDELEDG |
| KSYICSDMRKVKPINLALVRRKLPPWYHARPVSSRRRTVHQSRSFPGQKIHRKERAVLRTPKRLVVFCNGV |
| PAVHHTLVLDKRITPTFETILEYISEVVQFHVVKLHMLDGRRVDGLPGLILCSGVVVAAGREPFRAA |
|  |
| >DCXfish CAG00594 |
| SEKKAKKVRFYRNGDRYFKGIVYAVANDRFRTFDSLLADLTRSLSDHINLPQGVRFIFTIDGMNKITSLDEL |
| EEGESYVCASENFYKKVDYTKNVNPNWSVNVKASASQKNMQSLAAKAASEAREGKDFVRPKLVTVMRSGVKP |
| RKAVRVLLNKKTAHSFEQVLTDITEAIKLESGVVKRIYTLDGKQVTCLQDFFGDDDVFIACGPEKFRYAQDD |
|  |
| >DCLKfish CAG09017 |
| SEKKAKKVRFYRNGDRYFKGIVYAISQERFGSLEALLADLTRSLSDNVNLPQGVRTIYSVDGQTKITSIEQL |
| VEGESYVCASIEPYKKVDYTKNVNPNWSVSARTAVPTRDPSSLGSAKSACGEGRDNRDFIKPKLVTIIRSGV |
| KPRKAVRVLLNKKTAHSFDQVLTDITDAIKLDSGVVRRLYTVDGKMVTCLQDFFAEDDIFFACGPEKFRYQDD |
|  |
| >RP1zebfish XP_686596 |
| KRVCFYKSGDPQFTGHRMVINSRTFKTFDALLDALSKKVPLPFGVRTITTPRGTHAVCSLDDVQDGGSYLCS |
| DQKKVKPFNLDEVHKRQVPWNTTRPVSAGRQARRELVRQLAKRNQVSTRTIKMSENTVVVRTPKRLTVYKNR |
| DPSMKRVIVLHRRIAPTFEALLDYLSQMMQFPVVKLYTEDGRRIEGLSALILCTGIVVAAGNEPFR |
|  |
| >DCXzebfish XP_688131 |
| SEKKAKKVRFYRNGDRYFNGIVYAISSDRIRTFDALLADLTRTLSDNVNLPQGVRIIYSIDGNKKITNIDQ |
| LVEGESYVCGSTEAFKRVDYSNNVNPNWSVNVRALGSSKCPTSLASSKSGPQFRESKDFIRPKLVTVIRSG |
| VKPRKAVRILLNKKTAHSFEQVLTDITDAIKLDSGIVKRIY |
|  |
| >DCLK2zebfi XP_690913 |
| SEKKAKKVRFYRNGDKYFKGLVYAVSGDRFRSFDALLMELTRSLSDNVNLPQGVRSIYTADGGKKITSLD |
| DLVEGESYVCASNEPFRKVDYTKNVNPNWSVNVKTGASRSMPSLTATKNELRERESKDYIKPKLVTVIRS |
| GVKPRKAVRILLNKKTAHSFEQVLTDITDAIKLDSGAVKRLYTLEGKQ |
|  |
| >DCDC2Bzebf AAI08063 |
| KSVMVYRNGDPFFSGRRFVVNQRQIATMDALLNDITLNIGAPLAVRTLYTPRYGHRV |
| ADLGDLQQGAQYVAAGSERFKKLDY |
|  |
| >DCLKpan XP_509627 |
| SEKKAKKVRFYRNGDRYFKGIVYAISPDRFRSFEALLADLTRTLSDNVNLPQGVRTIYTIDGLKKISSLD |
| QLVEGESYVCGSIEPFKKLEYTKNVNPNWSVNVKTTSASRAVSSLATAKGSPSEVRENKDFIRPKLVTII |
| RSGVKPRKAVRILLNKKTAHSFEQVLTDITDAIKLDSGVVKRLYTLDGKQ |
|  |
| >DCXpan XP_529107 |
| NEKKAKKVRFYRNGDRYFKGIVYAVSSDRFRSFDALLADLTRSLSDNINLP |
| QGVRYIYTIDGSRKIGSMDELEEGESYVCSSDNFFKKVEYTKNVNPNWSVNVKTSANMKAPQSLASSNSA |
| QARENKDFVRPKLVTIIRSGVKPRKAVRVLLNKKTAHSFEQVLTDITEAIKLETGVVKKLYTLDGKQVTC |
| LHDFFGDDDVFIACGPEKFRYAQDD |
|  |
| >RP1pan XP_528138 |
| AKRISFYKSGDPQFGGVRVVVNPRSFKSFDALLDNLSRKVPLPFGVRNISTPRGR |
| HSITRLEELEDGESYLCSHGRKVQPVDLDKARRRPRPWLSSRAISAHSPPHP |
| VAVAAPGVPRPPRSLVVFRNGDPKTRRAVLLSRRVTQSFEAFLQHLTEVMQRPVVKLYA |
| TDGRRVPSLQAVILSSGAVVAAGREPFK |
|  |
| >DCDC2pan XP_527599 |
| KSVLVYRNGDPFYAGRRVVIHEKKVSSFEVFLKEVTGGVQAPFGAVRNIYTPRT |
| GHRIRKLDQIQSGGNYVAGGQEAFKKLNYLDIGEIKKRPMEVVNTEVKPVIHSRINVSA |
| RFRKPLQEPCTIFLIANGDLINPASRLLIPRKTLNQWDHVLQMVTEKITLRSGAVHRLY |
| TLEGKLVESGAELENGQFYVAVGRDKFK |
|  |
| >DCLK2pan XP_517476 |
| SEKKAKKARFYRNGDRYFKGLVFAISSDRFRSFDALLIELTRSLSDNVNLPQGVRTIYT |
| IDGSRKVTSLDELLEGESYVCASNEPFRKVDYTKNINPNWSVNIKGGTSRALAAASSVK |
| SEVKESKDFIKPKLVTVIRSGVKPRKAVRILLNKKTAHSFEQVLTDITEAIKLDSGVVK |
| RLCTLDGKQVRSHFGVSSCFRKVEVALPLGIGEALSLLSVTCLQDFFGDDDVFIACGPE |
| KFRYAQDD |
|  |
| >DCXdog XP_853182 |
| NEKKAKKVRFYRNGDRYFKGIVYAVSSDRFRSFDALLADLTRSLSDNINLPQGVRYIYTIDGSRKIGSMD |
| ELEEGESYVCSSDNFFKKVEYTKNVNPNWSVNVKTSANMKAPQSLASSNSAQARENKDFVRPKLVTIIRS |
| GVKPRKAVRVLLNKKTAHSFEQVLTDITEAIKLETGVVKKLYTLDGKQVTCLHDFFGDDDVFIACGPEKF |
| RYAQDD |
|  |
| >DCLKdog XP_858159 |
| SEKKAKKVRFYRNGDRYFKGIVYAISPDRFRSFEALLADLTRTLSDNVNLPQGVRTIYTIDGLKKISSLD |
| QLVEGESYVCGSIEPFKKLEYTKNVNPNWSVNVKTTSASRAVSSLATAKGSPSEVRENKDFIRPKLVTII |
| RSGVKPRKAVRILLNKKTAHSFEQVLTDITDAIKLDSGVVKRLYTLDGKQVMCLQDFFGDDDIFIACGPE |
| KFRYQDD |
|  |
| >RP1dog AAK58443 NP_001003040 |
| AKKISFYKSGDPQFGGVKVVVNPRSFKTFDALLDNLSRKVPLPFGVRNISTPRGRHSITRLEELEDGASY |
| LCSHRRKVQPVDLDKARRRPRPWLSSRAISAHAQRSPPTSIGAAGAPGMLRAPRRLLVFRNGDPKIRRVV |
| IVNRRVTQSFQAFLQHLTEVMRFPVTKLYATDGRKVPSLQAVILSSGAVVAAGREPFK |
|  |
| >RP1L1dog XP_543211.2 |
| AKKITFLKRGDPRFAGVRLAVDQRAFKSFGALMDELSQRVPLSFGVRSVTTPRGLH |
| GLSALEQLEDGGYYLCSDKKPPKTPSRPGWPQGRSSSAQQSRDFESRCEAPGTSS |
| SCKGPKAPRRIVLVKNGDPRFQQTVVLSHRNTRNLMAFLSKASDLLHFPVKQVYTT |
| SGKKVDSLKGLLHSPSVLVCAGYESFK |
|  |
| >DCLK2dog XP_539760 |
| SEKKAKKARFYRNGDRYFKGLLFAISGDRFRSFDALLMELTRSLSDNVNLPQGVRTIYTIDGSRKVTSLDELLE |
| GESYVCASNEPFRKVDYTKNINPNWSVNIKGGTTRALAAPSSVKSEVKESKDFIKPKLVTVIRSGVKPRK |
| AVRILLNKKTAHSFEQVLTDITEAIKLDSGVVKRLCTLDGKQVTCLQDFFGDDDVFIACGPEKFRYAQDD |
|  |
| >DCDC2dog XP_853515 |
| KSVLVYRNGDPFFAGRRVVIHEKKVSSFDVFLKEVTGGVQAPFGAVRNIYTPRTGHRIRKLDQLQ |
| SGGNYVAGGQEAFKKLNYLDIGEMKKRPMEVINTEVKPVMHSRINVSARFRKPLQEPCTIFLIANGDLIS |
| PASRLLIPRKTLNQWDHVLQMVTEKITLRTGAVHRLYTLEGKPVESGAELENGQFYVAVGRDKFK |
|  |
| >DCLKadog XP_858032 |
| SEKKAKKVRFYRNGDRYFKGIVYAISPDRFRSFEALLADLTRTLSDNVNLPQGVRTIYTIDGLKKISSLD |
| QLVEGESYVCGSIEPFKKLEYTKNVNPNWSVNVKTTSASRAVSSLATAKGSPSEVRENKDFIRPKLVTII |
| RSGVKPRKAVRILLNKKTAHSFEQVLTDITDAIKLDSGVVKRLYTLDGKQVMCLQDFFGDDDIFIACGPE |
| KFRYQDD |
|  |
| >RP1_BOVIN q8mj05 |
| AKRISFYKSGDPQFGGVRVVLNPRSFKTFDALLDNLSGKVPLPFGVRNISTPRGRHSITRLEELEDGQSY |
| LCSHGRKVQPVDLDKARRRPRPWLSSRALSTHVQRGPAPAAPGMLRAPRRLVVFRNGDPKTRRAIVLNRR |
| VTQSFEVFLQYLTQVMQRPVTKLYATDGRKVPSLQAVILSSGAVVAAGREPFK |
|  |
| >DCXcow XP_594176 |
| NEKKAKKVRFYRNGDRYFKGIVYAVSSDRFRSFDALLADLTRSLSDNINLPQGVRYIYTIDGSRKIGSMD |
| ELEEGESYVCSSDNFFKKVEYTKNVNPNWSVNVKTSANMKAPQSLASSNSAQARENKDFVRPKLVTIIRS |
| GVKPRKAVRVLLNKKTAHSFEQVLTDITEAIKLETGVVKKLYTLDGKQVTCLHDFFGDDDVFIACGPEKF |
| RYAQDD |
|  |
| >DCLK2cow XP_616231 |
| SEKKAKKARFYRNGDRYFKGLVFAISSDRFRSFDALLMELTRSLSDNVNLPQGVRTIYTIDGSRKVTSLDELLE |
| GESYVCASNEPFRKVDYTKNVNPNWSVNIKGGATRSAPPSSVKSEVKESKDFIKPKLVTVIRSGVKPRKA |
| VRILLNKKTAHSFEQVLTDITEAIKLDSGVVKRLCTLDGKQVTCLQDFFGDDDVFIACGPEKFRYAQDD |
|  |
| >DCLKcow XP_608869 |
| SEKKAKKVRFYRNGDRYFKGIVYAISPDRFRSFEALLADLTRTLSDNVNLPQGVRTIYTIDGLKKISSLD |
| QLLEGESYVCGSIEPFKKLEYTKNVNPNWSVNVKTTSASRAVSSLATAKGSPSEVRENKDFIRPKLVTII |
| RSGVKPRKAVRILLNKKTAHSFEQVLTDITDAIKLDSGVVKRLYTLDGKQ |
|  |
| >DCDC2cow XP_596512 |
| SQPVVKSVLVYRNGDPFFTGRRVVFHEKKVSSFDVFLKEVTGGVQAPFGAVRNIYTPRT |
| GHRIWRLDQIQSGGNYVAGGQESFKKLNYLDIGEIKKRPMEAVRTEVKPVTHSRINVSSRFRKPLLEPCT |
| IFLIANGDLINPASRLLIPRKALNQWDHVLQMITEKITLRSGAVHRLYTLEGKLVESGAELENGQFYVAV |
| GRDRFKKLPYSE |
|  |
| >RP1L1cow XP_872674 |
| QVTPAKKITFLKRGDPQFAGVRVAVHQRAFRSFGALMDELSQRVPLSFGVRSVTTPRGLHGLST |
| LEQLQDGGCYLCSDKKPPRTPGGLGQPQGRSPSAQQLREFEAPGTASTCKGLKASRRITLVKNGDPQLQQ |
| TVVLSHRNTRNLTAFLSKASDLLRFPVKHVYTTGGKRVDSLKALLRSPSVLVCAGLEPFRPLVTED |
|  |
| >DCDC2Bcow XP_592329 |
| GSPAAKRVVVYQNGDPFSPGRQLVVTQRRFPTLETFLCEVTSAVRAPVAVRALYTPYGHPVTDLADL |
| QNGGLYVAAGFEHFHKLPYVMGPLDGSYLLVPPAISSKCPCWGGGGIDFCFSEAKENVFRNGDLLSPPFS |
| LKLSQAASEDWEAVLKLLKEKVKLQSGAVRKGRGPSGPALGARVEPGGLALVPGELLPSRARHLGRAV |
|  |
| >ZYG-8 NP_499571 |
| KAKRLRFYRNGDQYFKGIQYALQSDRVKSMQPLMEDLMKTVICDSTALPHGIRHIFTIDGAQRITSVDQFED |
| GGGYVCSSTDAFKPVDYSRAAEPSWRLTLANRYNRHLETKKLALSVVEPCHENTDFVFPRIIKVIRNGVK |
| PRRISRHLLNKKTARSFDQVLRDLTFVVKLDSGAIRKLFTLSGRPVLSLQDFFRDDDVFVAYGGNEKMAA |
| DD |
|  |
| >cbZYG-8 CAE71285 |
| KAKRLRFYRNGDQYFKGIPYALQCDRVKSMQPLMEELMKTVICDSTALPHGIRHIFTMDGTQRITSVDQFE |
| DGGGYVCSSTDVFKPVDYSRAAEPSWRLTLANRYNRHLETKKLALNVVEPANENTDFVIPKIIKVIRNGV |
| KPRRISRHLLNKKTARSFDQVLRDLTLIVKLDAGAIRKLFTLSGRPVLTLQDFFREDDVFVAYGGNDKMA |
| ADD |
|  |
| >DdDCX AAO52397 |
| EKAKVIMCFRNGDRYHSGERVTVHSTKFKTYDQLKEQLSKQVKLPTGPVRKLYLASSGKLVKTM |
| EEIIDGEYYVCAGGETLNPLDFSPTLSEHVKQKKLQEQQQQASEQQKPQEQEIF |
|  |
| >cio-Sca_55 |
| AKNVHVYLNGDRFFPGRKFVVNRRHISDFDGFLNQVTVGMKAPFGAVRNIYTPNLGHRVRDLTQLQNGMDLVA |
| GGVERFRKIQY |
| KEPCQIYVYGNGDINAPAIRLLLIPRAMKSWDLVLSEITEKICLRTGKAVRKLYDMDYHLLGDPSELENGKYY |
| IAVGTERIKKIAY |
|  |
| >cio-Sca_14 |
| DEKRAKKVRFYRNGDNFHTGLVYAVSTTRFRTFEAMLIDLTNRLADKVHLPHGVRVVFSLDGARKIETIQELEHGE |
| SYVCASSDMFKRLEY |
| TIIRSGVKPRKAVRILLNKKTAHSLEQVLNDVTKAIKLDTGAVRKVYTLQGKQVQSLQDFFGNDDIFIAYGHEKL |
| SQDD |
|  |
| >cio-Sca_10 |
| RRPRIVMFYKNGDRYFKGKSLHITPHRYLHYEELLSDLSKSMS----LPYGVRRIYTPIGGTLIEDIEELKDGESY |
| VCASFEKFQRIKY |
| MKPKVVTVVRAGQIRPHKKITILLNRRAVQTYEQLVSDISEALGQPKWKNDHIRRLYTLKGREIRSVSDFFREDDV |
| FIAVGREQ |
|  |
| >DCDC2opp 19222 ++ 1043260 1115642 72383 |
| KSVLVYRNGDPFFAGRRVVIHEKKVSSFDVFLKEV |
| TGGVQAPFGAVRNIYTPRAGHRIRKLDQIQSGGNYVAGGL |
| EAFKKLVKPVIHSRINVSARFKKPLQEPCTILIANGDLIS |
| PASRLFIPRKALNQWDLVLEMVTEKITLRSGAVHRLYTLE |
| GKLVQSGSELENGQFYVAVGRDKFK |
|  |
| >DCLK2opp 13659 +- 4761486 4898250 |
| SEKKAKKARFYRNGDRYFKGLVYAISSDRFRSFDALLIEL |
| TRSLSDNVNLPQGVRTIYTIDGSRKLTSLEELMEGKSEG |
| KESXDFIKPKLVTVIRSGVKPRKAVRILLNKKTAHSFEQV |
| LTDITEAIKLDSGVVKRLCTLDGKQVTCLQDFFGDDDVFI |
| ACGPEKFRYAQDD |
|  |
| >DCLKopp 15015 ++ 10236870 10445067 |
| SEKKAKKVRFYRNGDRYFKGIVYAISPDRFRSFEALLADL |
| TRTLSDNVNLPQGVRTIYTIDGLKKISTLDQLVEGESYVC |
| GSIEPFKKLEYTKNVNPNWSVNVKTTSASRTVSSLATAKG |
| SPSDVRENKDFIRPKLVTIIRSGVKPRKAVRILLNKKTAH |
| SFEQVLTDITDAIKLDSGVVKRLYTLDGKQVMCLQDFFGD |
| DDIFIACGPEKFRYQDD |
|  |
| >DCXopp 15106 +- 349935 374589 24655 |
| NEKKAKKVRFYRNGDRYFKGIVYAVSTDRFRSFDALLADL |
| TRSLSDNINLPQGVRYIYTIDGGRKIGSMDELEEGDSYVC |
| SSDTFFKKVEYAKNANPNWSVNVKTSANLKAPQSLASSHS |
| AQARENKDFVRPKLVTIIRSGVKPRKAVRVLLNKKTAHSF |
| EQVLTDITEAIKLETGVVKKLYTLDGKQVTCLHDFFGDDD |
| VFIACGPEKFRYAQDD |
|  |
| >DCXrat X ++ 34649872 34701987 52116 |
| NEKKAKKVRFYRNGDRYFKGIVYAVSSDRFRSFDALLADL |
| TRSLSDNINLPQGVRYIYTIDGSRKIGSMDELEEGESYVC |
| SSDNFFKKVEYTKNVNPNWSVNVKTSANMKAPQSLASSNS |
| AQARENKDFVRPKLVTIIRSGVKPRKAVRVLLNKKTAHSF |
| EQVLTDITEAIKLETGVVKKLYTLDGKQVTCLHDFFGDDD |
| VFIACGPEKFRYAQDD |
|  |
| >DCDC2rat 17 +- 47031367 47093051 61685 |
| KSVLVYRNGDPFFAGRRVVIHEKKVSSFDIFLKEV |
| TGGVQAPFGAVRNIYTPRTGHRIRKLDQIESGGNYVAGGQ |
| EAFKKLYLDIGEIKKRPMEAVNTEVKPVIHSKINVSARF |
| RKALHEPCTILIANGDLISPASRLLIPRKALNQWDHVLQ |
| MVTEKITLRSGAVHRLYTLEGKLVESGAELENGQFYVAVG |
| RDKFK |
|  |
| >DCLK2rat 2 +- 178792593 178873428 80836 |
| SEKKAKKARFYRNGDRYFKGLVFAISSDRFRSFDALLIEL |
| TRSLSDNVNLPQGVRTIYTVDGSRKVTSLDELLEGESYVC |
| ASNEPFRKVDYTKNVNPNWSVNIKGGTTRTLAVASAKSEV |
| KESKDFIKPKLVTVIRSGVKPRKAVRILLNKKTAHSFEQV |
| LTDITEAIKLDSGVVKRLCTLDGKQVTCLQDFFGDDDVFI |
| ACGPEKYRYAQDD |
|  |
| >DCLKrat 2 ++ 144416788 144549734 132947 |
| SEKKAKKVRFYRNGDRYFKGIVYAISPDRFRSFEALLADL |
| TRTLSDNVNLPQGVRTIYTIDGLKKISSLDQLVEGESYVC |
| GSIEPFKKLEYTKNVNPNWSVNVKTTSASRAVSSLATAKG |
| GPSEVRENKDFIRPKLVTIIRSGVKPRKAVRILLNKKTAH |
| SFEQVLTDITDAIKLDSGVVKRLYTLDGKQVMCLQDFFGD |
| DDIFIACGPEKFRY |
|  |
| >RP1rat 5 ++ 15393188 15394548 1361 |
| AKRISFYKSGDPQFGGVRVVVNPRSFKTFDALLDNL |
| SRKVPLPFGVRNISTPRGRHSITKLEELEDGESYVCSHNK |
| KVLPVDLDKARRRPRPWLSSRSISTHVKLRSATATMPTTA |
| PGLFRAPRRLVVFRNGDPKTRRVVLLSRRITQSFEAFLQY |
| LTQVMQYPVAKLYATDGRKVPSLQAVILSSGAVVAAGREPFK |
|  |
|  |
|  |

N-terminal DCX domains

| >DCX_MOU-N NP_034155 |
| --- |
| NEKKAKKVRFYRNGDRYFKGIVYAVSSDRFRSFDALLADLTRSLSDNINLPQGVRYIYTIDGSRKIGSMD |
| ELEEGESYVCSSDNFFKKVEY |
|  |
| >DCLK_MOU-N NP_064362 |
| SEKKAKKVRFYRNGDRYFKGIVYAISPDRFRSFEALLADLTRTLSDNVNLPQGVRTIYTIDGLKKISSLD |
| QLVEGESYVCGSIEPFKKLEY |
|  |
| >RP1_MOU-N NP_035413.1 |
| HPVVAKRISFYKSGDPQFGGVRVVVNPRSFKTFDALLDSLS |
| RKVPLPFGVRNISTPRGRHSITRLEELEDGKSYVCSHNKKVLPVDL |
|  |
| >DCDC2_MO-N AAH45136.1 |
| SQPVVKSVLVYRNGDPFFAGRRVVIHEKKVSSFDVFLKEVTGGVQAPFGAVRNIYTPRT |
| GHRIRKLDQIESGGNYVAGGPEAFKKLNY |
|  |
| >RP1L1_MO-N Q8CGM2 |
| AKKITFLKRGDPQFAGVRLAVHQRTFKTFSSLMDELSQRMPLSFGVRSVTTPRGLHGLSALEQLQDGGCYLCS |
|  |
| >DCLK2_MO-N NP_081815.3 |
| SEKKAKKARFYRNGDRYFKGLVFAISNDRFRSFDALLIELTRSLSDNVNLPQGVRTIYTIDGSRKVTSLDELLE |
| GESYVCASNEPFRKVDY |
|  |
| >DCDC2A_MO XP_917846 |
| AKTILVYRNGDQFYVGRKFVFSRRRVANFEALLEQLTEQVEVPFGVRRLYTPTRGHPVLGLDA |
| LQTGGKYVAAGRERFKKLE |
|  |
| >DCDC2B_MOU XP_357395 |
| AKRILVYRNGDAFFPGHQLVVTQRRYPTMEALLYEVTSAVQAPLAVRVLYTLSDGHPVTNLADLQNGG |
| QYVAAGFERFHKIHV |
|  |
| >mBAC26042 |
| RVKRVWAYQNGGRHADGTYVQAGALSELLDGCTVRLKMSHPAKTLYTSNGELIQSWDDIERGMAVCVSAGHGF |
|  |
| >DCX_HUM-N O43602 |
| NEKKAKKVRFYRNGDRYFKGIVYAVSSDRFRSFDALLADLTRSLSDNINLP |
| QGVRYIYTIDGSRKIGSMDELEEGESYVCSSDNFFKKVEY |
|  |
| >DCLK_HUM-N NP_004725 |
| SEKKAKKVRFYRNGDRYFKGIVYAISPDRFRSFEALLADLTRTLSDNVNLPQGVRTIYTIDGLKKIS |
| SLDQLVEGESYVCGSIEPFKKLEY |
|  |
| >DCLK2_HU-N AAH32726 |
| SEKKAKKARFYRNGDRYFKGLVFAISSDRFRSFDALLIELTRSLSDNVNLPQGVRTIYTIDGSRKVTSLDELLE |
| GESYVCASNEPFRKVDY |
|  |
| >DCDC2A_HUM XP_371476 |
| AKTIVVYRNGDPFYVGKKFVLSRRRAATFEALLEQLTEQVDVPFGVRRLFTPTRGH |
| RVLGLDALQAGGKYVAAGRERFKELDY |
|  |
| >RP1_HUM-N AAD44198 |
| AKRISFYKSGDPQFGGVRVVVNPRSFKSFDALLDNL |
| SRKVPLPFGVRNISTPRGRHSITRLEELEDGESYLCSHGRKVQPVDL |
|  |
| >DCDC2_HU-N AAF23612 |
| KSVLVYRNGDPFYAGRRVVIHEKKVSSFEVFLKEVTGGVQAPFGAVRNIYTPRT |
| GHRIRKLDQIQSGGNYVAGGQEAFKKLNY |
|  |
| >RP1L1_HU-N NP_849188 |
| AKKITFLKRGDPRFAGVRLAVHQRAFKTFSALMDELSQ |
| RVPLSFGVRSVTTPRGLHSLSALEQLEDGGCYLCSDKKPPK |
|  |
| >hFLJ-N NP_940864 |
| QKAVKIIAYKNGDGYRNGKLIVAGTFPMLLTECTEQLGLARAASKVYTKDGT |
|  |
| >DCDC2B_HU-N XP_497666 |
| AKRVVVYRNGDPFFPGSQLVVTQRRFPTMEAFLCEVTSAVQAPLAVRALYTPCHGHPVTNLAD |
| LKNRGQYVAAGFERFHKL |
|  |
| >DCDC1_HUMAN P59894 |
| KVTAYKNGSRTVFARVTAPTITLLLEECTEKLNLNMAARRVFLADGKEALEPEDIPHEADVYVSTG |
|  |
| >DCDC2Ach-N XP_417793 |
| LAHPAKNVVVYRNGDPFFHGRKFVVNQRQFLTFEAFLNEVTKSIHAPLAVRNLYTPKHGHRVA |
| ELADLQDGCQYVAAGFEKFKRLDP |
|  |
| >DCLKchi-N XP_417099 |
| SEKKAKKVRFYRNGDRYFKGIVYAISPDRFRSFEALLADLTRTLSDNVNLPQGVRTIYTIDGSKKISSLDQ |
| LVEGESYVCGSIEPFKKLEY |
|  |
| >RP1L1chi-N XP_426222 |
| QVPPAKKITFFKSGDPQFGGVKMAINQRSFKSFNALMDDLSHRVPLPFGVRTITTPRGIHCISELDQLEDGGC |
| YLCSDKKYVKPIN |
|  |
| >RP1chick-N XP_426089 |
| EPVVAKRICFYKSGDPQFNGIKMVINNRSYKTFDALLDSLSKRVPLPFGVRNISTPKGRHSITCLDDLEDGKS |
| YICSHQRKMKPIN |
|  |
| >DCLK2chi-N XP_420439 |
| SEKKARKARFYRNGDKYFKGLVYAISSDRFRSFDALLAELTRSLSDNVNLPQGVRTIYTIDGSKKLSSL |
| DELLEGESYVCASNEPYRKVDY |
|  |
| >DCXchick-N gAAK15319-N AF330009_1 doublecortin [Gallus gallus] |
| NEKKAKKVRFYRNGDRYFKGIVY |
| AVSSDRFRSFDALLADLTRSLSDNINLPQGVRYIYTIDGSRKIGSMDELEEGESYVCSSDNFFKKVEY |
|  |
| >plaEAA2096 EAA20968 |
| KSIWLYRNGDKHHNGLLFFIKSHINNLKLLLFEITKVLNPIIGPIRKIYDQNFRLIKNIQQ |
| LNDGSKYLCTS |
|  |
| >H.roretz-N BAB40784 HrDoublecortin [Halocynthia roretzi] |
| AKNVHVYVNGDQYYPGRKFVVNRRYICDFDGFLNLVTSGLKPSFGAVRNIYT |
| PNAGHRVNELHEINNNMVIVAGGAERFRKLHYQ |
|  |
| >DCDC2fro-N AAH46665 MGC52970 protein [Xenopus laevis] |
| QQPAVKTVHVFRNGDPFYRGRRMLIHERRVGTFDVFLKDVTGGVQAPFGAVR |
| NIYTPRNGHRVTSLDDLQPGEFYVAGGRENFKKLDY |
|  |
| >RP1frog-N AAH78105 |
| EPASTKRVCFYKSGDPQFNGIKMVVSNRSFKTFDALLDT |
| LSKKVPLPFGVRNISTPRGIHHVTSINELEDGKSYICSHRKKIKPIN |
|  |
| >DdDCX-N AAO52397 |
| EKAKVIMCFRNGDRYHSGERVTVHSTKFKTYDQLKEQLSKQVKLPTGPVRKLYLASSGKL |
| VKTMEEIIDGEYYVCAGGETLNPLDFS |
|  |
| >DCXdog-N XP_853182 |
| NEKKAKKVRFYRNGDRYFKGIVYAVSSDRFRSFDALLADLTRSLSDNINLPQGVRYIYTIDGSRKIGSMD |
| ELEEGESYVCSSDNFFKKVEY |
|  |
| >DCLKdog-N XP_858159 |
| SEKKAKKVRFYRNGDRYFKGIVYAISPDRFRSFEALLADLTRTLSDNVNLPQGVRTIYTIDGLKKISSLDQ |
| LVEGESYVCGSIEPFKKLEY |
|  |
| >DCLKadog-N XP_858032 |
| SEKKAKKVRFYRNGDRYFKGIVYAISPDRFRSFEALLADLTRTLSDNVNLPQGVRTIYTIDGLKKISSLDQ |
| LVEGESYVCGSIEPFKKLEY |
|  |
| >DCLK2dog-N XP_539760 |
| SEKKAKKARFYRNGDRYFKGLLFAISGDRFRSFDALLMELTRSLSDNVNLPQGVRTIYTIDGSRKVTSLDELLE |
| GESYVCASNEPFRKVDY |
|  |
| >DCDC2dog-N XP_853515 |
| SQPVVKSVLVYRNGDPFFAGRRVVIHEKKVSSFDVFLKEVTGGVQAPFGAVRNIYTPRTGHRIRKLDQLQ |
| SGGNYVAGGQEAFKKLNY |
|  |
| >DCDC2Bdog N XP_854671 |
| AKRVVIYRNGDPYFPGHQLVVSQRRFPTLETFLQEVTSIVQAPVAVRALYTPCHGH |
| RVTDLADLQNGGQYVAAGFERFCKL |
|  |
| >RP1dog-N AAK58443 |
| AKKISFYKSGDPQFGGVKVVVNPRSFKTFDALLDNLSRKVPLPFGVRNISTPRGRHSITRLEELEDGASY |
| LCSHRRKVQPVDLD |
|  |
| >RP1L1dog XP_543211.2 |
| KKITFLKRGDPRFAGVRLAVDQRAFKSFGALMDELSQRVPLSFGVRSVTTPRGLH |
| GLSALEQLEDGGYYLCS |
|  |
| >DCDC2Adog XP_850203 |
| RPAPAKAIVVYRNGDAFLEGRRCVLSRRRAATFEALLDQLTEQVEVPFGVRRLFTPTRGHR |
| VLELQALQAGGKYVAAGREPFKKLDI |
|  |
| >DCDC2Bdo-N XP_854671 |
| GNPAAKRVVIYRNGDPYFPGHQLVVSQRRFPTLETFLQEVTSIVQAPVAVRALYTPCHGHRVTDLA |
| DLQNGGQYVAAGFERFCKLHV |
|  |
| >FLJdog-NC XP_533167 |
| QKAVKIIAYKNGDGYRNGKLIVAGTFPTLLTECTEQLGLTRAASKVYTKDGTTVLSLRD |
|  |
| >DCDC1dog XP_540561 |
| SEKTSVRILFFKNGMGQDGHEITVGKETIKKVLDTCTMKMKLNLPARYLYDLYGRKIKDISKGK |
|  |
| >DCXcow-N XP_594176 |
| NEKKAKKVRFYRNGDRYFKGIVYAVSSDRFRSFDALLADLTRSLSDNINLPQGVRYIYTIDGSRKIGSMD |
| ELEEGESYVCSSDNFFKKVEY |
|  |
| >DCLK2cow-N XP_616231 |
| SEKKAKKARFYRNGDRYFKGLVFAISSDRFRSFDALLMELTRSLSDNVNLPQGVRTIYTIDGSRKVTSLDELLE |
| GESYVCASNEPFRKVDY |
|  |
| >DCLKcow-N XP_608869 |
| SEKKAKKVRFYRNGDRYFKGIVYAISPDRFRSFEALLADLTRTLSDNVNLPQGVRTIYTIDGLKKISSLD |
| QLLEGESYVCGSIEPFKKLEY |
|  |
| >RP1_BOVIN-N |
| AKRISFYKSGDPQFGGVRVVLNPRSFKTFDALLDNLSGKVPLPFGVRNISTPRGRHSITRLEELEDGQSY |
| LCSHGRKVQPVDL |
|  |
| >DCDC2cow-N XP_596512 |
| SQPVVKSVLVYRNGDPFFTGRRVVFHEKKVSSFDVFLKEVTGGVQAPFGAVRNIYTPRT |
|  |
| >RP1L1cow-N XP_872674 |
| QVTPAKKITFLKRGDPQFAGVRVAVHQRAFRSFGALMDELSQRVPLSFGVRSVTTPRGLHGLST |
| LEQLQDGGCYLCSDKKPPRTPGGLG |
|  |
| >DCDC2Bco-N XP_592329 |
| GSPAAKRVVVYQNGDPFSPGRQLVVTQRRFPTLETFLCEVTSAVRAPVAVRALYTPYGHPVTDLADL |
| QNGGLYVAAGFEHFHKLPY |
|  |
| >DCDC2Ac-NC scaffold550 |
| KTIVVYRNGDQFYVGRKFVLSRRRVATFEALLEQLTEQVEVPFGVRRLFTPTRGR |
| PVLELDSLQAGGKYVAAGRERFKKLE |
|  |
|  |
| >FLJcow-N XP_608048 |
| QKAVKIIAYKNGDGYRNGKLIVAGTFPMLLTECTEQLGLARAASKIYTKDGT |
|  |
| >bovXP_8740 XP_874039 |
| LLEECTEKLNLNTAARRVFLADGTEALEPEDIPREADVYVSTGESFLDPFKKIK |
| ~ |
| >anoEAL4118 EAL41186 |
| KARRVLFYRNGDPFFPGVEFRFKPGRDICTLEALLDKISARMDLP |
| RGARYIFSMDGDRKYSLDELEDGSSYVVSSFKVFKKSK |
|  |
| >drNP_64872 NP_648728 |
| KARRVVFYRNGDPFFPGVELRYRPGRDVTSLDNLLDKISPKMDLPRGARYVFSMDGDRKYHLDELEDGAFY |
| VVSSFKAFKLVCRLCV |
|  |
| >A.mellif XP_393742 |
| RARRVTFYKNGDPYFPGIEFRFKPGRD |
| IGSLEALLDRLSLRMDLPRGARHIFSMDGDRKLTLDELEDGASSTIESASSSS |
|  |
| >drNP_65115 NP_651150 |
| KKALRVCFLRNGDRHFKGVNLVISRAHFKDFPALLQGVTESLKRHVLLRSAI |
| AHFRRTDGSHLTSLSCFRETDIVICCCKNEEIICVKY |
|  |
| >anoXP_3197 XP_319785 |
| KKAKRIRFFRNGDKFYPGSTIPVSVERYRSFDSLTEDLTRLLEDSVTLTGAIRAIYTL |
| EGKKIEKVDDLEDGKCYVCSCNNEGFKRIDY |
|  |
| >A.mellif-N XP_394386 |
| KKAKRVRFFRNGDKFYTGIVMAVTPERYRSFDSLATDLTRALISSVTLPNGVRAIYTMDGKKVQSIND |
| LEDGKCYVVSGQGEIFKKVEY |
|  |
| >D.melano-N AAM11416 |
| RKALRIKFYRNGDRFYPGITIPVSNERYRSFERLFEDLTRLLEENVKIPGAVRTIY |
| NLCGKKITSLDELEDGQSYVCSCNNENFKKVEY |
|  |
| >DCXfish-N CAG00594 |
| SEKKAKKVRFYRNGDRYFKGIVYAVANDRFRTFDSLLADLTRSLSDHINLPQGVRFI |
| FTIDGMNKITSLDELEEGESYVCASENFYKKVDY |
|  |
| >DCLKafish-N CAG00429 |
| SEEKRAKKVRFYRNGDRYFNGIVYAISTDRFRTFDALLADLTRSLSDNVNLPQGVRTIYTLDGTKKIGA |
| IDQLVEGDSYVCSSNEAYKKLDY |
|  |
| >DCLKfish-N CAG09017 |
| SEKKAKKVRFYRNGDRYFKGIVYAISQERFGSLEALLADLTRSLSDNVNLPQGVRTIYSVDGQTKITSI |
| EQLVEGESYVCASIEPYKKVDY |
|  |
| >DCLK2fis-N CAG02200 |
| SEKKAKKVRFYRNGDRYFKGLVYAVSSDRFRSYDALLMELTRSLSDNLYLPQGVRTIYSVDGSKKIGSMDE |
| LVEGESYVCASNEPYKKLEY |
|  |
| >DCLK2zeb-N XP_690913 |
| SEKKAKKVRFYRNGDKYFKGLVYAVSGDRFRSFDALLMELTRSLSDNVNLPQGVRSIYTADGGKKITSLDDL |
| VEGESYVCASNEPFRKVDY |
|  |
| >DCLKzebfi-N XP_688131 |
| SEKKAKKVRFYRNGDRYFNGIVYAISSDRIRTFDALLADLTRTLSDNVNLPQGVRIIYSIDGNKKITNID |
| QLVEGESYVCGSTEAFKRVDY |
|  |
| >DCDC2Bze-N AAI08063 |
| KSVMVYRNGDPFFSGRRFVVNQRQIATMDALLNDITLNIGAPLAVRTLYTPRYGHRV |
| ADLGDLQQGAQYVAAGSERFKKLDY |
|  |
| >RP1zebfi-N XP_686596 |
| KRVCFYKSGDPQFTGHRMVINSRTFKTFDALLDALSKKVPLPFGVRTITTPRGTHAVCSLDDVQ |
| DGGSYLCSDQKKVKPFNLDE |
|  |
| >RP1fish-N CAG10227 |
| SKRVCFYKSGDAQFSGLRMVINNRTFNTFDALLDS |
| LSRKVPLPFGVRNITTPHGVHAVHTLDELEDGKSYICSDMRKVKPINL |
|  |
| >DCLK2azebf danXP_6892 XP_689225 |
| SEKRAKKVRFYRNGDRYFKGLVYAVSSDRFRSMDALLAELTRALADNLHLPQGVRNIYTAD |
| GAKKISSLEELAEGESYVCASNEPYRKVDY |
|  |
| >tetCAG1045 CAG10453 |
| KSVVVYKNGDPFYTGRRFVVNQRQVATMEAFLNEVTQSIGAPLAIRTLYTPRQGHRVPDLQH |
| LQTGAQYVAAGFEKFKKMDYL |
|  |
| >DCDC2fis-N CAF96719 |
| KNIFMFRNGDPYYEARRIVINQKRVSNFETLLREVTGGIQAPFGAVRTIYTPRGGHK |
|  |
| >ZYG-8-N NP_499571 |
| HLLKAKRLRFYRNGDQYFKGIQYALQSDRVKSMQPLMEDLMKTVICDSTALPHGIRHIFTIDGAQRITSVDQFED |
| GGGYVCSSTDAFKPVDY |
|  |
| >cbZYG-8-N CAE71285 |
| HLVKAKRLRFYRNGDQYFKGIPYALQCDRVKSMQPLMEELMKTVICDSTALPHGIRHIFTMDGTQRITSVDQFE |
| DGGGYVCSSTDVFKPVDY |
|  |
| >ceNP_00102 NP_001022398 |
| RKISIYKNGDRYHRGVKFVINPRVIKDMEPLLNQVN |
| DRIELSHGAKKLYTTDGKIVNSIKELEDGKIYVAASAQF |
|  |
| >ceCAB04937 |
| RKISIYKNGDRYHRGVKFVINPRVIKDMEPLLNQVN |
| DRIELSHGAKKLYTTDGKIVNSIKELEDGKIYVAASAQF |
|  |
| >cbCAE73319 |
| NAAGVKRIHVWRNADVFFPGIQVVVNTHRVPSIDVLLDVVSERIGLINGAKKLYTTSGTLIKDINK |
| IKDGENYVASSSHF |
|  |
| >cbCAE73316 |
| RKISIYKNGDRFHRGVKFVINPRVVKDMEPLLNQINDRIELSHGAKKLYTTDGKVVGSIKELEDGKI |
| YVAASAQF |
|  |
| >cio-Sca_14_N |
| DEKRAKKVRFYRNGDNFHTGLVYAVSTTRFRTFEAMLIDLTNRLADKVHLPHGVRVVFSLDGARKIETIQELEHGE |
| SYVCASSDMFKRLEY |
|  |
| >cio-Sca_10_N |
| RRPRIVMFYKNGDRYFKGKSLHITPHRYLHYEELLSDLSKSMSLPYGVRRIYTPIGGTLIEDIEELKDGESY |
| VCASFEKFQRIKY |
|  |
| >cio-Sca_55_N |
| AKNVHVYLNGDRFFPGRKFVVNRRHISDFDGFLNQVTVGMKAPFGAVRNIYTPNLGHRVRDLTQLQNGMDLVA |
| GGVERFRKIQY |
|  |
| >urcXP_7883 XP_788383 |
| RRVMVFLNGDASESHEVVANLDQFNQFLDSCTSKLNLNCPARYIYTWDGQKLEDLNELPRLDGCLQSS |
|  |
| >DCDC2Aopp NC 18691 +- 165667 165912 246 |
| ARTILVYRNGDEFYVGTKFVINRKRVPNIEALMTQLNDKLAVPFGVRRLYTPCQGH |
| RILELEQLQQGGKYVAAGRERF |
|  |
| >DCDC2opp-N 19222 ++ 1043260 1115642 72383 |
| KSVLVYRNGDPFFAGRRVVIHEKKVSSFDVFLKEVTGGVQAPFGAVRNIYTPRAGH |
| RIRKLDQIQSGGNYVAGGLEAFKKL |
|  |
| >DCLK2opp-N 13659 +- 4761486 4898250 |
| SEKKAKKARFYRNGDRYFKGLVYAISSDRFRSFDALLiEL |
| TRSLSDNVNLPQGVRTIYTIDGSRKLTSLEELMEGKSEGKESDFIK |
|  |
| >DCLKopp-N 15015 ++ 10236870 10445067 |
| SEKKAKKVRFYRNGDRYFKGIVYAISPDRFRSFEALLADL |
| TRTLSDNVNLPQGVRTIYTIDGLKKISTLDQLVEGESYVC |
| GSIEPFKKLEY |
|  |
| >DCXopp-N 15106 +- 349935 374589 24655 |
| NEKKAKKVRFYRNGDRYFKGIVYAVSTDRFRSFDALLADL |
| TRSLSDNINLPQGVRYIYTIDGGRKIGSMDELEEGDSYVC |
| SSDTFFKKVEY |
|  |
| >RP1opp-NC 18689 ++ 5473251 5477669 4419 |
| AKRISFYKSGDPQFNGIQMVVNPRSFKSFDALLDNLSKKVPLPFGVRNISTPRGIH |
| GITKLEDLEDGRSYICSHKKKIKPID |
|  |
| >RP1L1opp-N 14808 ++ 1510891 1511148 258 |
| AKKITFLKRGDPRFAGVRLAVHQRAFKTFSALMDELSQRVPLSFGVRSVTTPRGLH |
| SLSALEQLEDGGCYLCS |
|  |
| >BAC26042ra 3 ++ 91789851 91789988 138 |
| LLDDCTARLKMSHPAKTLYTSNGELIQSWDEIEKGMAVCVSAGHGF |
|  |
| >DCXrat-N X ++ 34649872 34701987 52116 |
| NEKKAKKVRFYRNGDRYFKGIVYAVSSDRFRSFDALLADL |
| TRSLSDNINLPQGVRYIYTIDGSRKIGSMDELEEGESYVC |
| SSDNFFKKVEY |
|  |
| >DCDC2Arat NC 6 +- 46422760 46423005 246 |
| AKTILVYRNGDQFYVGRKFVFSRRRVANFEALLEQLTEQV |
| EVPFGVRRLYTPTYGHRVLELESLQTGGKYVAAGRERFKKLE |
|  |
| >DCDC2rat-N 17 +- 47031367 47093051 61685 |
| KSVLVYRNGDPFFAGRRVVIHEKKVSSFDIFLKEV |
| TGGVQAPFGAVRNIYTPRTGHRIRKLDQIESGGNYVAGGQ |
| EAFKKLY |
|  |
| >DCLK2rat-N 2 +- 178792593 178873428 80836 |
| SEKKAKKARFYRNGDRYFKGLVFAISSDRFRSFDALLIEL |
| TRSLSDNVNLPQGVRTIYTVDGSRKVTSLDELLEGESYVC |
| ASNEPFRKVDY |
|  |
| >DCLKrat-N 2 ++ 144416788 144549734 132947 |
| SEKKAKKVRFYRNGDRYFKGIVYAISPDRFRSFEALLADL |
| TRTLSDNVNLPQGVRTIYTIDGLKKISSLDQLVEGESYVC |
| GSIEPFKKLEY |
|  |
| >RP1rat-N 5 ++ 15393188 15394548 1361 |
| AKRISFYKSGDPQFGGVRVVVNPRSFKTFDALLDNL |
| SRKVPLPFGVRNISTPRGRHSITKLEELEDGESYVCSHNK |
| KVLPVDLD |
|  |
| >FLJrat-NC FLJ46154 |
| QKAVKIIAYKNGNGYRNGKLIVAGTFHGLLAECTERLQLTRSASKIYTRDGT |
|  |
| >DCDC2Apan NC XP_525677 |
| AKTIVVYRNGDPFYVGKKFVLSRSRAATFEALLEQLTEQVDVPFGVRRLFTPTRGH |
| RVLGLDALQAGGKYVAAGRERFKELE |
|  |
| >DCDC2Bpa-N XP_513279 |
| AKRVVVYRNGDPFFPGSQLVVTQRRFPTMEAFLCEVTSAVQAPLAVRALYTPCHGH |
| PVTNLADLKNRGQYVAAGFERFHKLHY |
|  |
| >DCDC2pan-N XP_527599 |
| KSVLVYRNGDPFYAGRRVVIHEKKVSSFEVFLKEVTGGVQAPFGAVRNIYTPRT |
| GHRIRKLDQIQSGGNYVAGGQEAFKKLNY |
|  |
| >DCLK2pan-N XP_517476 |
| SEKKAKKARFYRNGDRYFKGLVFAISSDRFRSFDALLIELTRSLSDNVNLPQGVRTIYT |
| IDGSRKVTSLDELLEGESYVCASNEPFRKVDY |
|  |
| >DCLK3pa-NC BAB21856 XP_526170 |
| KPRVVTVVKLGGQRPRKITLLLNRRSVQTFEQLLADISEALGSPRWKNDRVRKLFNLKGR |
| EIRSVSDFFREGDAFI |
|  |
| >FLJpan-NC FLJ46154 |
| QKAVKIIAYKNGDGYRNGKLIVAGTFPMLLTECTEQLGLTRAASKVYTKDGTCTE |
| ILNLPSAARRLYNEKGKEIFALKDLQRDE |
|  |
| >RP1pan-N XP_528138 |
| AKRISFYKSGDPQFGGVRVVVNPRSFKSFDALLDNLSRKVPLPFGVRNISTPRGR |
| HSITRLEELEDGESYLCSHGRKVQPVDL |
|  |
| >DCDC1pan XP_508349 |
| KVTAFKNGSRTVFARVTVPTITLLLEKCTEKLNLNMAARRVFLADGKEALEPEVIPHEADVYVSTGEPFLNPFKKIK |
|  |
| >DCLKpan-N XP_509627 |
| SEKKAKKVRFYRNGDRYFKGIVYAISPDRFRSFEALLADLTRTLSDNVNLPQGVRTIYTIDGLKKISSLDQLV |
| EGESYVCGSIEPFKKLEY |
|  |
| >DCXpan-N XP_529107 |
| NEKKAKKVRFYRNGDRYFKGIVYAVSSDRFRSFDALLADLTRSLSDNINLP |
| QGVRYIYTIDGSRKIGSMDELEEGESYVCSSDNFFKKVEY |
|  |
|  |

C-terminal DCX proteins

| >DCX_MOU-C NP_034155 |
| --- |
| DFVRPKLVTIIRSGVKPRKAVRVLLNKKTAHSFEQVLTDITEAIKLETGVVKKLYTLDGKQVTCLHDFF |
| GDDDVFIACGPEKFRYAQDD |
|  |
| >DCLK_MOU-C NP_064362 |
| DFIRPKLVTIIRSGVKPRKAVRILLNKKTAHSFEQVLTDITDAIKLDSGVVKRLYTLDGKQVMCLQDFF |
| GDDDIFIACGPEKFRYQDD |
|  |
| >RP1_MOU-C NP_035413.1 |
| MLRAPRRLVVFRNGDPKNKHVVLLSRRITQSFEAFLQYLTQVMQCPVAKLYATDGRKVP |
| SLQAVILSSGAVVAAGREPFKPGNYD |
|  |
| >DCDC2_MO-C AAH45136.1 |
| PCTIFLIANGDLISPASRLLIPKKALNQWDHVLQMVTEKITLRSGAVHRLYTLEGKLVESGAELENGQFYVAV |
| GRDKFKRLPYSE |
|  |
| >RP1L1_MO-C Q8CGM2 |
| VAPRRLTLVKNGDPRRQQTVVLSHKNTRSLAAFLGKASELLRFPVKQVYTTRGKKVDSLQTLLDGPSVLVCAGNEAFR |
|  |
| >DCLK2_MO-C NP_081815.3 |
| DFIKPKLVTVIRSGVKPRKAVRILLNKKTAHSFEQVLTDITEAIKLDSGVVKRLCTLDGKQVTCLQDFFGDDDV |
| FIACGPEKYRYAQDD |
|  |
| >DCLK3_MOU NP_766516 |
| HSPLKPRVVTVVKLGGQPLRKATLLLNRRSVQTFEQLLSDISEALGFPRW |
| KNDRVRKLFTLKGREVKSVSDFFREGDAFIAMGKE |
|  |
| >DCDC2A_MOU XP_917846 |
| DTTPAKTILVYRNGDQFYVGRKFVFSRRRVANFEALLEQLTEQVEVPFGVRRLYTPTRGHPVL |
| GLDALQTGGKYVAAGRERFK |
|  |
| >mFLJ-C XP_489892 |
| EGLLDTNSSPMKRMASKRPDLLVPMRLRVLRNGEKKNIRP |
| LRQEPRVKRTQCTDILNLPSAARRLFSEKGKELFSLKDLQRDELVYVSCG |
|  |
| >DCDC2B_MOU XP_357395 |
| KRILVYRNGDAFFPGHQLVVTQRRYPTMEALLYEVTSAVQAPLAVRVLYTLSDGHPVTNLADLQNGG |
| QYVAAGFERF |
|  |
| >DCX_HUM-C O43602 |
| DFVRPKLVTIIRSGVKPRKAVRVLLNKKTAHSFEQVLTDITEAIKLETGVVKKLYTLDGKQVTC |
| LHDFFGDDDVFIACGPEKFRYAQDD |
|  |
| >DCLK_HUM-C NP_004725 |
| DFIRPKLVTIIRSGVKPRKAVRILLNKKTA |
| HSFEQVLTDITDAIKLDSGVVKRLYTLDGKQVMCLQDFFGDDDIFIACGPEKFRYQDD |
|  |
| >DCLK2_HU-C AAH32726 |
| DFIKPKLVTVIRSGVKPRKAVRILLNKKTAHSFEQVLTDITEAIKLDSGVVKRLCTLDGKQVTCLQ |
| DFFGDDDVFIACGPEKFRYAQDD |
|  |
| >RP1_HUM-C AAD44198 |
| PRSLVVFRNGDPKTRRAVLLSRRVTQSFEAFLQHLTEVMQRPVVKLYATDGRRVPSLQ |
| AVILSSGAVVAAGREPFK |
|  |
| >DCDC2_HU-C AAF23612 |
| PLQEPCTIFLIANGDLINPASRLLIPRKTLNQWDHVLQMVTEKITLRSGAVHRLYTLEGKL |
| VESGAELENGQFYVAVGRDKFK |
|  |
| >RP1L1_HU-C NP_849188 |
| PRRILLIKNMDPRLQQTVVLSHRNTRNLAAFLGKASDLLRFPVKQLYTTSGKKVDSLQAL |
| LHSPSVLVCAGHEAFR |
|  |
| >DCLK3_HUM BAB21856 |
| LKPRVVTVVKLGGQRPRKITLLLNRRSVQTFEQLLADISEALGSPRWKNDRVRKLFNLKGRE |
| IRSVSDFFREGDAFIAMGKEP |
|  |
| >hFLJ-C NP_940864 |
| TQCTEILNLPSAARRLYNEKGKEIFALKDLQRDELVYVSCG |
|  |
| >DCDC2A_HUM XP_371476 |
| DTTPAKTIVVYRNGDPFYVGKKFVLSRRRAATFEALLEQLTEQVDVPFGVRRLFTPTRGH |
| RVLGLDALQAGGKYVAAGRERFK |
|  |
| >DCDC2B_HUM C XP_497666 |
| FERFHKLHVFRNGDLVSPPFSLKLSQAASQDWETVLKLLTEKVKLQSGAVCKLCTLEGLP |
| LSAGKELVTGHYYVAVGEDEFK |
|  |
| >DCDC2Ach-C XP_417793 |
| ALAHPAKNVVVYRNGDPFFHGRKFVVNQRQFLTFEAFLNEVTKSIHAPLAVRNLYTPKHGHRVA |
| ELADLQDGCQYVAAGFEKFKRLDPVP |
|  |
| >RP1L1chi-C XP_426222 |
| GPRIPKKITLVKNGETSFRRSIILNRRNARSFKTLLDEISEILQFPVKKLFTVD |
| GKKIDSMQALLHCPNVLVCVGREPFKPVSME |
|  |
| >RP1chick-C XP_426089 |
| KITTPKKMLVFKNGDVRLRRTIVLGKKNTQTFEAFLDYMSELMQYPVAKLYTTD |
| GRKVPNLQALILCSGAIVAAGREPFKPSNYE |
|  |
| >DCLKchi-C XP_417099 |
| DFIRPKLVTIIRSGVKPRKAVRILLNKKTAHSFEQVLTDITDAIKLDSGVVKRLYTLDG |
| KQIFLAFRLIAAVDARAFWHTSGKHDMNFIAPPLPVAAGWE |
| VVMVMCLQDFFGDDDIFIACGPEKFRYQDD |
|  |
| >DCDC2chick XP_418912 galsDCD2A-C |
| PYTIFLIANGDLISPVVRLLIPRKTLNHWDHILEMVTAKVSLRSGAVHRLYTLDG |
| KHVQNGSDLEKGQFYVAVGREKFKK |
|  |
| >DCDC2Bchic XP_417802 galDCD2B-NC |
| IHVFRNGDLLSPPFQLMISKSTLWQWDTLLATLTEKADLCSGAVNRLCKLDGTLVSSREELVNGNYYV |
| AVGTEEYKK |
|  |
| >DCLK2chi-C XP_420439 |
| DFIKPKLVTVIRSGVKPRKAVRILLNKKTAHSFEQVL |
| TDITEAIKLDSGVVKRLCTLDRKQVTCLQDFFGDDDVFIACGPEKYRYAQDD |
|  |
| >DCXchick-C AAK15319 |
| DFVRPKLVTIIRSGVKPRKAVRVLLNKKTAHSFEQV |
| LTDITEAIKLETGVVKKLYTLDGKQVTCLHDFFGDDDVFIACGPEKFRYAQDD |
|  |
| >DCXcow-C XP_594176 |
| DFVRPKLVTIIRSGVKPRKAVRVLLNKKTAHSFEQVLTDITEAIKLETGVVKKLYTLDGKQVTCLHDFF |
| GDDDVFIACGPEKFRYAQDD |
|  |
| >DCLK2cow-C XP_616231 |
| DFIKPKLVTVIRSGVKPRKAVRILLNKKTAHSFEQVLTDITEAIKLDSGVVKRLCTLDGKQVTCLQDFFG |
| DDDVFIACGPEKFRYAQDD |
|  |
| >DCLKcow-C XP_608869 |
| DFIRPKLVTIIRSGVKPRKAVRILLNKKTAHSFEQVLTDITDAIKLDSGVVKRLYTLDGKQ |
|  |
| >FLJcow-C XP_608048 |
| FHQLLERCTEILNLPSAARRLFNEKGKEVFTLKELQRDELVYVSCG |
|  |
| >RP1_BOV-C |
| GMLRAPRRLVVFRNGDPKTRRAIVLNRRVTQSFEVFLQYLTQVMQRPVTKLYATDGRKVPSLQA |
| VILSSGAVVAAGREPFK |
|  |
| >DCDC2cow-C XP_596512 |
| PLLEPCTIFLIANGDLINPASRLLIPRKALNQWDHVLQMITEKITLRSGAVHRLYTLEGKLVESGAELENGQFYVAV |
| GRDRFKKLPYSE |
|  |
| >RP1L1cow-C XP_872674 |
| GLKASRRITLVKNGDPQLQQ |
| TVVLSHRNTRNLTAFLSKASDLLRFPVKHVYTTGGKRVDSLKALLRSPSVLVCAGLEPFRPLVTED |
|  |
| >DCDC2Bco-C XP_592329 |
| GSPAAKRVVVYQNGDPFSPGRQLVVTQRRFPTLETFLCEVTSAVRAPVAVRALYTPYGHPVTDLADL |
| QNGGLYVAAGFEHFHKLPYVM |
|  |
| >DCDC2Ac-NC scaffold550 |
| KTIVVYRNGDQFYVGRKFVLSRRRVATFEALLEQLTEQVEVPFGVRRLFTPTRGRPVLEL |
| DSLQAGGKYVAAGRERFK |
|  |
| >BAB21856co XP_583775 |
| SLLKPRVVTVVKVGSHPLRKITLLLNRRSVQTFEQLLADVSEALGFPRWKSDRVRKLFNLKGREIRSVSDFFRE |
| GDAFIAVGKEPLTLKNIQVAIEEL |
|  |
| >H.roretz-C BAB40784 |
| AIKMLLSARIMKNWDMVLEEITEKISIRTGQAVRRLYTLDGVLIQGSENLEN |
| GRYYVAVGYERFKRA |
|  |
| >DdDCX-C AAO52397 |
| EGVHCLVHSSKFKTFDQLKLEFSKKVGLFTGNVQKVYSMDKKRIQDIKDFVDGHHYICCGAE |
|  |
| >DCXdog-C XP_853182 |
| DFVRPKLVTIIRSGVKPRKAVRVLLNKKTAHSFEQVLTDITEAIKLETGVVKKLYTLDGKQVTCLHDFFGD |
| DDVFIACGPEKFRYAQDD |
|  |
| >DCLKadog-C XP_858032 |
| DFIRPKLVTIIRSGVKPRKAVRILLNKKTAHSFEQVLTDITDAIKLDSGVVKRLYTLDGKQVMCLQDFFGD |
| DDIFIACGPEKFRYQDD |
|  |
| >DCLK2dog-C XP_539760 |
| DFIKPKLVTVIRSGVKPRKAVRILLNKKTAHSFEQVLTDITEAIKLDSGVVKRLCTLDGKQVTCLQDFFGDD |
| DVFIACGPEKFRYAQDD |
|  |
| >DCDC2dog-C XP_853515 |
| PLQEPCTIFLIANGDLISPASRLLIPRKTLNQWDHVLQMVTEKITLRTGAVHRLYTLEGKPVESGAELENGQ |
| FYVAVGRDKFKKLPY |
|  |
| >DCDC2Bdo-C XP_854671 |
| VFRNGDLLSPPFSLKLSQAASEDWETVLKLLTEKAKLQAGAVCKLCTLEGLPLSAREAL |
| VNGHYYVAVGEEEFK |
|  |
| >DCLKdog-C XP_858159 |
| DFIRPKLVTIIRSGVKPRKAVRILLNKKTAHSFEQVLTDITDAIKLDSGVVKRLYTLDGKQVMCLQDFFGD |
| DDIFIACGPEKFRYQDD |
|  |
| >RP1dog-C CAAK58443 |
| MLRAPRRLLVFRNGDPKIRRVVIVNRRVTQSFQAFLQHLTEVMRFPVTKLYATDGRKVPSLQAVILSSGAV |
| VAAGREPFK |
|  |
| >RP1L1dog-C XP_543211.2 |
| PRRIVLVKNGDPRFQQTVVLSHRNTRNLMAFLSKASDLLHFPVKQVYTTSGKKVDS |
| LKGLLHSPSVLVCAGYESFK |
|  |
| >FLJdog-NC XP_533167 |
| QKAVKIIAYKNGDGYRNGKLIVAGTFPTLLTECTEQLGLTRAASKVYTKDGTTVLSLRDLVLWALDESF |
| IQRNTEK |
|  |
| >DCDC2fro-C AAH46665 |
| PCTVFLVANGDTLNPFIRLLIPRKTLEQWELVLALVTEKVKLRNGAVHRLYTLEGTPIQNGLELENG |
| QFYVAVGREKFKK |
|  |
| >RP1frog-C AAH78105 |
| LNKKTKQSFDSFLDQVAEALQYPVFKLYSSDGRRILSIRALLLSSGTVVAAGRESFIYA |
|  |
| >A.mellifer XP_393742 |
| RVLLNLRTSQPFEEVLEDLGQVLKMNGAKRMFTVSGQEVRSFSQLRNEFADVDTF |
|  |
| >EMAL_DROME |
| EVLEDLGQVLKINGAKKMYTGTGQEVRSFSQLRNEFADVDTFYLATGTALIAGSPIRR |
|  |
| >anoEAA0645 EAA06456 |
| GSLKPSAGRVIRIINSHDHSVQCRVLLNLRTSQPFE |
| EVLEDLGQVLKMIGAKKMYTSNGQEVRSFSQLRNEFAEVETFYLSNTPSLPVGALGP |
|  |
| >drNP_99582 NP_995824 |
| GNMRLLITNLLKDCLLDHKIVRVLVRCM |
| EQLITDTNDRIQYFIEIIYELCELNTKQNDLIHDRSLINKLLDDLDTPLKMKISSLKVKILELEELE |
|  |
| >A.gambia-C EAA14780 |
| SVVHPRIVTLIRNGVKPRKILRLLLNKRNSPTYEHVLTAITQCVKLDTGCVRKV |
| FTVAGVPVQRLAQFFEEDDVFFAYGNER |
|  |
| >A.mellif-C XP_394386 |
| LCVKAKIITLIRHGTKPRKVVRLLLNKRNAPSLEHALEA |
| ITEAVKLDSGAVRKVYTLSGQQVTSLEQFFENDDIFVAYGPEK |
|  |
| >D.melano-C AAM11416 |
| VHPRIVTLIRSGTKSRRIMRLLLNKRNSPSFDHVLTAITQV |
| VRLDTGYVRKVFTLSGIPVVRLSDFFGSDDVFFAYGTERINTAED |
|  |
| >DCXfish-C CAG00594 |
| DFVRPKLVTVMRSGVKPRKAVRVLLNKKTAHSFEQV |
| LTDITEAIKLESGVVKRIYTLDGKQVTCLQDFFGDDDVFIACGPEKFRYAQDD |
|  |
| >DCLKafish-C CAG00429 |
| DFIRPKLVTVVRSGVKPRKAVRILLNKK |
| TAHSYEQVLTDITDAIKLDSGVVKKIYTLEGKLVSCLQDFFGDEDVFVACGPEKFRYQDD |
|  |
| >DCLKfish-C CAG09017 |
| DFIKPKLVTIIRSGVKPRKAVRVLLNKKTAHSFDQ |
| VLTDITDAIKLDSGVVRRLYTVDGKMVTCLQDFFAEDDIFFACGPEKFRYQDD |
|  |
| >DCLK2fis-C CAG02200 |
| DFIKPKLVTVIRSGVKPRKAVRILLNKKTAHSFEQVLADITEAIKLDSGAVKRLYTLDGKQLTCLQ |
| DFFGDDDVFMACGPEKFRYAQDD |
|  |
| >DCLK2zeb-C XP_690913 |
| DYIKPKLVTVIRSGVKPRKAVRILLNKKTAHSFEQVLTDITDAIKLDSGAVKRLYTLEGKQ |
|  |
| >DCLKzebfi-C XP_688131 |
| DFIRPKLVTVIRSGVKPRKAVRILLNKKTAHSFEQVL |
| TDITDAIKLDSGIVKRIYTLEGKQTAFIKKEREELTI |
|  |
| >DCDC2Bze-C AAI08063 |
| PCIIHVFRNGDILSPAMRLIIPRHMLKNLEQILSLISEKAMLRTGAVRRICTLEGFTVTSAEELE |
| TGQCYVAVGSERFKKLPY |
|  |
| >RP1fish-C CAG10227 |
| VLRTPKRLVVFCNGVPAVHHTLVLDKRITPTFETILEYISEVVQFHVVKLHM |
| LDGRRVDGLPGLILCSGVVVAAGREPFRAANY |
|  |
| >DCDC2fis-C CAF96719 |
| PCAVFVVANGDVLNSAVRLLIHQRMLGQFDKILEMITEKMGLRVLGGVRSLYTYDGTQVNDGNQL |
| ESGQLYVAVGRERFKK |
|  |
| >tetCAG1227 CAG12276 |
| PKKLVVISNRDPTFKRTIVLHRRSAPTFDALLDYLSQILQFPVLK |
| LYSTDGRRIDGLAALILCSGVVVAAGNEPFK |
|  |
| >RP1zebfish-C XP_686596 |
| PKRLTVYKNRDPSMKRVIVLHRRIAPTFEALLDYLSQMMQ |
| FPVVKLYTEDGRRIEGLSALILCTGIVVAAGNEPFR |
|  |
| >ZYG-8-C NP_499571 |
| DFVFPRIIKVIRNGVKPRRISRHLLNKKTARSFDQVLRDLTFVVKLDSGAIRKLFTL |
| SGRPVLSLQDFFRDDDVFVAYG |
|  |
| >cbZYG-8-C AE71285 |
| DFVIPKIIKVIRNGVKPRRISRHLLNKKTARSFDQVLRDLTLIVKLDAGAIRKLFTLSGRPVLTLQDFFREDD |
| VFVAYGGNDKMAADD |
|  |
| >ceNP_00102 NP_001022398 |
| ISIYKNGDRYHRGVKFVINPRVIKDMEPLLNQVN |
| DRIELSHGAKKLYTTDGKIVNSIKELEDGKIYVAASAQ |
|  |
| >cio-Sca_14_C |
| TIIRSGVKPRKAVRILLNKKTAHSLEQVLNDVTKAIKLDTGAVRKVYTLQGKQVQSLQDFFGNDDIFIAYGHEKL |
| SQDD |
|  |
| >cio-Sca_10_C |
| MKPKVVTVVRAGQIRPHKKITILLNRRAVQTYEQLVSDISEALGQPKWKNDHIRRLYTLKGREIRSVSDFFREDDV |
| FIAVGREQ |
|  |
| >cio-Sca_55_C |
| KEPCQIYVYGNGDINAPAIRLLLIPRAMKSWDLVLSEITEKICLRTGKAVRKLYDMDYHLLGDPSELENGKYY |
| IAVGTERIKKIAY |
|  |
| >urcXP_7883 XP_788383 |
| RSAASSVSTYSQSSKGKFQNRLQPHVIRTMCYRNGSREKSVKITAPTMKIFLEYCTLKLDFEFAARRIFLED |
| GTEVKSAEEIPRDGEVYISGGEPFK |
|  |
| >DCDC2Aopp NC 18691 +- 165667 165912 246 |
| DITPARTILVYRNGDEFYVGTKFVINRKRVPNIEALMTQLNDKLAVPFGVRRLYTPCQG |
| HRILELEQLQQGGKYVAAGRERF |
|  |
| >DCDC2opp-C 19222 ++ 1043260 1115642 72383 |
| LIANGDLISPASRLFIPRKALNQWDLVLEMVTEKITLRSGAVHRLYTLEGKLVQSGSEL |
| ENGQFYVAVGRDKFK |
|  |
| >DCLK2opp-C 13659 +- 4761486 4898250 |
| DFIKPKLVTVIRSGVKPRKAVRILLNKKTAHSFEQVLTDITEAIKLDSGVVKRLCTLDGK |
| QVTCLQDFFGDDDVFIACGPEKFRYAQDD |
|  |
| >DCLK3opp BAB21856 18708 +- 521183 521383 201 |
| RKITLLLNRRSVLTFEQLVADISEALGFPRWKNDRVRKLYSLKGKEVKSVSDFFREGDAF |
| IAMGRE |
|  |
| >DCLKopp-C 15015 ++ 10236870 10445067 |
| DFIRPKLVTIIRSGVKPRKAVRILLNKKTAH |
| SFEQVLTDITDAIKLDSGVVKRLYTLDGKQVMCLQDFFGD |
| DDIFIACGPEKFRYQDD |
|  |
| >DCXopp-C 15106 +- 349935 374589 24655 |
| DFVRPKLVTIIRSGVKPRKAVRVLLNKKTAHSF |
| EQVLTDITEAIKLETGVVKKLYTLDGKQVTCLHDFFGDDD |
| VFIACGPEKFRYAQDD |
|  |
| >RP1opp-NC 18689 ++ 5473251 5477669 4419 |
| KRISFYKSGDPQFNGIQMVVNPRSFKSFDALLDNLSKKVPLPFGVRNISTPRGIHGITKL |
| EDLEDGRSYICSH |
|  |
| >DCXrat-C X ++ 34649872 34701987 52116 |
| DFVRPKLVTIIRSGVKPRKAVRVLLNKKTAHSF |
| EQVLTDITEAIKLETGVVKKLYTLDGKQVTCLHDFFGDDD |
| VFIACGPEKFRYAQDD |
|  |
| >DCDC2Arat NC 6 +- 46422760 46423005 246 |
| KTILVYRNGDQFYVGRKFVFSRRRVANFEALLEQLTEQV |
| EVPFGVRRLYTPTYGHRVLELESLQTGGKYVAAGRERFK |
|  |
| >DCDC2rat-C 17 +- 47031367 47093051 61685 |
| LIANGDLISPASRLLIPRKALNQWDHVLQMVTEKITLRSGAVHRLYTL |
| EGKLVESGAELENGQFYVAVGRDKFKRLPYSE |
|  |
| >DCLK2rat-C 2 +- 178792593 178873428 80836 |
| DFIKPKLVTVIRSGVKPRKAVRILLNKKTAHSFEQV |
| LTDITEAIKLDSGVVKRLCTLDGKQVTCLQDFFGDDDVFI |
| ACGPEKYRYAQDD |
|  |
| >DCLK3rat BAB21856 8 ++ 115879697 115879951 255 |
| LKPRVVTVVKLGVQPLRKATLLLNRRSVQTFEQLLSDISEALGFPRWKNDRVRKLFTLKG |
| REVKSVSDFFREGDAFIAMGKE |
|  |
| >DCLKrat-C 2 ++ 144416788 144549734 132947 |
| DFIRPKLVTIIRSGVKPRKAVRILLNKKTAH |
| SFEQVLTDITDAIKLDSGVVKRLYTLDGKQVMCLQDFFGD |
| DDIFIACGPEKFRYQD |
|  |
| >RP1rat 5 ++ 15393188 15394548 1361 |
| FRAPRRLVVFRNGDPKTRRVVLLSRRITQSFEAFLQY |
| LTQVMQYPVAKLYATDGRKVPSLQAVILSSGAVVAAGREPFKP |
|  |
| >FLJrat-NC FLJ46154 |
| QKAVKIIAYKNGNGYRNGKLIVAGTFHGLLAECTERLQLTRSASKIYTRDG |
|  |
| >DCDC2Apan NC XP_525677 |
| KTIVVYRNGDPFYVGKKFVLSRSRAATFEALLEQLTEQVDVPFGVRRLFTPTRGHRVLGL |
| DALQAGGKYVAAGRERFK |
|  |
| >DCDC2Bpa-C XP_513279 |
| PTYIHVFRNGDLVSPPFSLKLSQAASQDWETVLKLLTEKVKLQSGAVCKLCTLEGLPLSA |
| GEELV-TGHYYVAVGEDEFK |
|  |
| >DCDC2pan-C XP_527599 |
| PCTIFLIANGDLINPASRLLIPRKTLNQWDHVLQMVTEKITLRSGAVHRLY |
| TLEGKLVESGAELENGQFYVAVGRDKFK |
|  |
| >DCLK2pan-C XP_517476 |
| DFIKPKLVTVIRSGVKPRKAVRILLNKKTAHSFEQVLTDITEAIKLDSGVVK |
| RLCTLDGKQVRSHFGVSSCFRKVEVALPLGIGEALSLLSVTCLQDFFGDDDVFIACGPE |
| KFRYAQDD |
|  |
| >DCLK3pa-NC BAB21856 XP_526170 |
| VVTVVKLGGQRPRKITLLLNRRSVQTFEQLLADISEALGSPRWKNDRVRKLFNLKG |
| REIRSVSDFFREGDAFIAMGKE |
|  |
| >DCLKpan-C XP_509627 |
| DFIRPKLVTIIRSGVKPRKAVRILLNKKTAHSFEQVLTDITDAIKLDSGVVKRLYTLDGKQ |
|  |
| >DCXpan-C XP_529107 |
| DFVRPKLVTIIRSGVKPRKAVRVLLNKKTAHSFEQVLTDITEAIKLETGVVKKLYTLDGKQVTC |
| LHDFFGDDDVFIACGPEKFRYAQDD |
|  |
| >FLJpan-NC FLJ46154 |
| QKAVKIIAYKNGDGYRNGKLIVAGTFPMLLTECTEQLGLTRAASKVYTKDG |
|  |
| >RP1L1pan-C XP_528138 |
| VLSHRNTRNLAAFLSKASDLLRFPVKQLYTTSGKKVDSLQALLHSPSVLVCAGHEAFR |
|  |
| >RP1pan-C XP_528138 |
| PRSLVVFRNGDPKTRRAVLLSRRVTQSFEAFLQHLTEVMQRPVVKLYA |
| TDGRRVPSLQAVILSSGAVVAAGREPFK |
|  |
|  |
|  |
