# Supplementary material for: The evolving doublecortin (DCX) superfamily
Source: BMC Genomics. 2006 Jul 26;7:188. doi: 10.1186/1471-2164-7-188 (PMC1550402; doi:10.1186/1471-2164-7-188)
Supplement: Additional File 2 — Supplementary Fig. 2: Synthenic positions of mouse and human DCX domain proteins. [file 1471-2164-7-188-S2.doc]

mouse np_034155

Type: inv

Level: 2

Mouse position: chrX:90020770-142249359

Human position: chrX:51317131-114330337

mouse np_064362

Type: top

Level: 1

Mouse position: chr3:52388208-57319439

Human position: chr13:33360534-40146741

Strand: -

mouse np_081815

Type: top

Level: 1

Mouse position: chr3:76161566-87075932

Human position: chr4:151323988-163454117

Strand: -

mouse np_035413

Type: top

Level: 1

Mouse position: chr1:3091336-7232466

Human position: chr8:52856992-56686113

Strand: -

mouse xp_920003

Type: top

Level: 1

Mouse position: chr14:57898323-59483566

Human position: chr8:9744662-11880616

Strand: -

mouse aah45136

Type: top

Level: 1

Mouse position: chr13:20647742-29644161

Human position: chr6:20173202-28613177

Strand: -

mouse xp_917846

Type: top

Level: 1

Mouse position: chr12:3165573-28843840

Human position: chr2:164193-26272361

Strand: -

mouse XP_357395

Type: top

Level: 1

Mouse position: chr4:102689921-155175443

Human position: chr1:1009792-58724787

Strand: -

mouse xp_916728 replaced by NP_766516

Type: top

Level: 1

Mouse position: chr9:89942515-118168557

Human position: chr3:27728694-149570190

Strand: -

mouse xp_489892

Type: top

Level: 1

Mouse position: chr2:84326378-110843247

Human position: chr11:26252973-57510519

Strand: -

mouse bac26042

Type: top

Level: 1

Mouse position: chr2:84326378-110843247

Human position: chr11:26252973-57510519

Strand: -
