# Supplementary material for: The evolving doublecortin (DCX) superfamily
Source: BMC Genomics. 2006 Jul 26;7:188. doi: 10.1186/1471-2164-7-188 (PMC1550402; doi:10.1186/1471-2164-7-188)
Supplement: Additional File 3 — Supplementary Fig. 3: CLUSTAL W (1.83) multiple sequence alignment of the DCX proteins with tandem domains, N-terminal domains and C-terminal domains. [file 1471-2164-7-188-S3.doc]

Protein Alignments

Proteins with tandem DCX domains

CLUSTAL W (1.83) multiple sequence alignment

RP1_MOUSE HPVVAKRISFYKSGDPQFGGVRVVVNPRSFKTFDALLDSLSR-----KVPLPFG-VRNIS

RP1rat ----AKRISFYKSGDPQFGGVRVVVNPRSFKTFDALLDNLSR-----KVPLPFG-VRNIS

RP1_HUMAN ----AKRISFYKSGDPQFGGVRVVVNPRSFKSFDALLDNLSR-----KVPLPFG-VRNIS

RP1pan ----AKRISFYKSGDPQFGGVRVVVNPRSFKSFDALLDNLSR-----KVPLPFG-VRNIS

RP1dog ----AKKISFYKSGDPQFGGVKVVVNPRSFKTFDALLDNLSR-----KVPLPFG-VRNIS

RP1_BOVIN ----AKRISFYKSGDPQFGGVRVVLNPRSFKTFDALLDNLSG-----KVPLPFG-VRNIS

RP1chick ----AKRICFYKSGDPQFNGIKMVINNRSYKTFDALLDSLSK-----RVPLPFG-VRNIS

RP1frog EPASTKRVCFYKSGDPQFNGIKMVVSNRSFKTFDALLDTLSK-----KVPLPFG-VRNIS

RP1fish ---RSKRVCFYKSGDAQFSGLRMVINNRTFNTFDALLDSLSR-----KVPLPFG-VRNIT

RP1zebfish -----KRVCFYKSGDPQFTGHRMVINSRTFKTFDALLDALSK-----KVPLPFG-VRTIT

RP1L1_HUMA ----AKKITFLKRGDPRFAGVRLAVHQRAFKTFSALMDELSQ-----RVPLSFG-VRSVT

RP1L1dog ----AKKITFLKRGDPRFAGVRLAVDQRAFKSFGALMDELSQ-----RVPLSFG-VRSVT

RP1L1cow QVTPAKKITFLKRGDPQFAGVRVAVHQRAFRSFGALMDELSQ-----RVPLSFG-VRSVT

RP1L1_MOUS ----AKKITFLKRGDPQFAGVRLAVHQRTFKTFSSLMDELSQ-----RMPLSFG-VRSVT

RP1L1chick ----AKKITFFKSGDPQFGGVKMAINQRSFKSFNALMDDLSH-----RVPLPFG-VRTIT

DCX_MOUSE NEKKAKKVRFYRNGDRYFKGIVYAVSSDRFRSFDALLADLTR-SLSDNINLPQG-VRYIY

DCX_HUMAN NEKKAKKVRFYRNGDRYFKGIVYAVSSDRFRSFDALLADLTR-SLSDNINLPQG-VRYIY

DCXpan NEKKAKKVRFYRNGDRYFKGIVYAVSSDRFRSFDALLADLTR-SLSDNINLPQG-VRYIY

DCXdog NEKKAKKVRFYRNGDRYFKGIVYAVSSDRFRSFDALLADLTR-SLSDNINLPQG-VRYIY

DCXcow NEKKAKKVRFYRNGDRYFKGIVYAVSSDRFRSFDALLADLTR-SLSDNINLPQG-VRYIY

DCXrat NEKKAKKVRFYRNGDRYFKGIVYAVSSDRFRSFDALLADLTR-SLSDNINLPQG-VRYIY

DCXchick NEKKAKKVRFYRNGDRYFKGIVYAVSSDRFRSFDALLADLTR-SLSDNINLPQG-VRYIY

DCXopp NEKKAKKVRFYRNGDRYFKGIVYAVSTDRFRSFDALLADLTR-SLSDNINLPQG-VRYIY

DCXfish SEKKAKKVRFYRNGDRYFKGIVYAVANDRFRTFDSLLADLTR-SLSDHINLPQG-VRFIF

DCLKpan SEKKAKKVRFYRNGDRYFKGIVYAISPDRFRSFEALLADLTR-TLSDNVNLPQG-VRTIY

DCLKcow SEKKAKKVRFYRNGDRYFKGIVYAISPDRFRSFEALLADLTR-TLSDNVNLPQG-VRTIY

DCLK_HUMAN SEKKAKKVRFYRNGDRYFKGIVYAISPDRFRSFEALLADLTR-TLSDNVNLPQG-VRTIY

DCLKdog SEKKAKKVRFYRNGDRYFKGIVYAISPDRFRSFEALLADLTR-TLSDNVNLPQG-VRTIY

DCLKadog SEKKAKKVRFYRNGDRYFKGIVYAISPDRFRSFEALLADLTR-TLSDNVNLPQG-VRTIY

DCLK_MOUSE SEKKAKKVRFYRNGDRYFKGIVYAISPDRFRSFEALLADLTR-TLSDNVNLPQG-VRTIY

DCLKrat SEKKAKKVRFYRNGDRYFKGIVYAISPDRFRSFEALLADLTR-TLSDNVNLPQG-VRTIY

DCLKopp SEKKAKKVRFYRNGDRYFKGIVYAISPDRFRSFEALLADLTR-TLSDNVNLPQG-VRTIY

DCLKchick SEKKAKKVRFYRNGDRYFKGIVYAISPDRFRSFEALLADLTR-TLSDNVNLPQG-VRTIY

DCLK2zebfi SEKKAKKVRFYRNGDKYFKGLVYAVSGDRFRSFDALLMELTR-SLSDNVNLPQG-VRSIY

DCLKafish EEKRAKKVRFYRNGDRYFNGIVYAISTDRFRTFDALLADLTR-SLSDNVNLPQG-VRTIY

DCXzebfish SEKKAKKVRFYRNGDRYFNGIVYAISSDRIRTFDALLADLTR-TLSDNVNLPQG-VRIIY

DCLKfish SEKKAKKVRFYRNGDRYFKGIVYAISQERFGSLEALLADLTR-SLSDNVNLPQG-VRTIY

DCLK2_MOUS SEKKAKKARFYRNGDRYFKGLVFAISNDRFRSFDALLIELTR-SLSDNVNLPQG-VRTIY

DCLK2rat SEKKAKKARFYRNGDRYFKGLVFAISSDRFRSFDALLIELTR-SLSDNVNLPQG-VRTIY

DCLK2_HUMA SEKKAKKARFYRNGDRYFKGLVFAISSDRFRSFDALLIELTR-SLSDNVNLPQG-VRTIY

DCLK2pan SEKKAKKARFYRNGDRYFKGLVFAISSDRFRSFDALLIELTR-SLSDNVNLPQG-VRTIY

DCLK2dog SEKKAKKARFYRNGDRYFKGLLFAISGDRFRSFDALLMELTR-SLSDNVNLPQG-VRTIY

DCLK2cow SEKKAKKARFYRNGDRYFKGLVFAISSDRFRSFDALLMELTR-SLSDNVNLPQG-VRTIY

DCLK2opp SEKKAKKARFYRNGDRYFKGLVYAISSDRFRSFDALLIELTR-SLSDNVNLPQG-VRTIY

DCLK2chick SEKKARKARFYRNGDKYFKGLVYAISSDRFRSFDALLAELTR-SLSDNVNLPQG-VRTIY

DCLK2fish SEKKAKKVRFYRNGDRYFKGLVYAVSSDRFRSYDALLMELTR-SLSDNLYLPQG-VRTIY

cio-Sca_14 DEKRAKKVRFYRNGDNFHTGLVYAVSTTRFRTFEAMLIDLTN-RLADKVHLPHG-VRVVF

A.gambiae PAKKAKRIRFFRNGDKFYPGSTIPVSVERYRSFDSLTEDLTR-LLEDSVTLTGA-IRAIY

D.melanoga PTRKALRIKFYRNGDRFYPGITIPVSNERYRSFERLFEDLTR-LLEENVKIPGA-VRTIY

A.mellifer PTKKAKRVRFFRNGDKFYTGIVMAVTPERYRSFDSLATDLTR-ALISSVTLPNG-VRAIY

ZYG-8 ---KAKRLRFYRNGDQYFKGIQYALQSDRVKSMQPLMEDLMKTVICDSTALPHG-IRHIF

cbZYG-8 ---KAKRLRFYRNGDQYFKGIPYALQCDRVKSMQPLMEELMKTVICDSTALPHG-IRHIF

cio-Sca_10 --RRPRIVMFYKNGDRYFKGKSLHITPHRYLHYEELLSDLSK-SMS----LPYG-VRRIY

DdDCX --EKAKVIMCFRNGDRYHSGERVTVHSTKFKTYDQLKEQLSK-----QVKLPTGPVRKLY

DCDC2_MOUS SQPVVKSVLVYRNGDPFFAGRRVVIHEKKVSSFDVFLKEVTG-----GVQAPFGAVRNIY

DCDC2rat -----KSVLVYRNGDPFFAGRRVVIHEKKVSSFDIFLKEVTG-----GVQAPFGAVRNIY

DCDC2_HUMA -----KSVLVYRNGDPFYAGRRVVIHEKKVSSFEVFLKEVTG-----GVQAPFGAVRNIY

DCDC2pan -----KSVLVYRNGDPFYAGRRVVIHEKKVSSFEVFLKEVTG-----GVQAPFGAVRNIY

DCDC2dog -----KSVLVYRNGDPFFAGRRVVIHEKKVSSFDVFLKEVTG-----GVQAPFGAVRNIY

DCDC2opp -----KSVLVYRNGDPFFAGRRVVIHEKKVSSFDVFLKEVTG-----GVQAPFGAVRNIY

DCDC2cow SQPVVKSVLVYRNGDPFFTGRRVVFHEKKVSSFDVFLKEVTG-----GVQAPFGAVRNIY

DCDC2frog QQPAVKTVHVFRNGDPFYRGRRMLIHERRVGTFDVFLKDVTG-----GVQAPFGAVRNIY

DCDC2fish SQPVVKNIFMFRNGDPYYEARRIVINQKRVSNFETLLREVTG-----GIQAPFGAVRTIY

h.roretzi QPETAKNVHVYVNGDQYYPGRKFVVNRRYICDFDGFLNLVTS-----GLKPSFGAVRNIY

cio-Sca_55 ----AKNVHVYLNGDRFFPGRKFVVNRRHISDFDGFLNQVTV-----GMKAPFGAVRNIY

DCDC2Bzebf -----KSVMVYRNGDPFFSGRRFVVNQRQIATMDALLNDITL-----NIGAPL-AVRTLY

DCDC2Bcow GSPAAKRVVVYQNGDPFSPGRQLVVTQRRFPTLETFLCEVTS-----AVRAPV-AVRALY

** . . : : . :* :

RP1_MOUSE TPRGRHSITRLEELEDGKSYVCS-HNKKVLPVDLDKARRRPRPWLS-----------SRS

RP1rat TPRGRHSITKLEELEDGESYVCS-HNKKVLPVDLDKARRRPRPWLS-----------SRS

RP1_HUMAN TPRGRHSITRLEELEDGESYLCS-HGRKVQPVDLDKARRRPRPWLS-----------SRA

RP1pan TPRGRHSITRLEELEDGESYLCS-HGRKVQPVDLDKARRRPRPWLS-----------SRA

RP1dog TPRGRHSITRLEELEDGASYLCS-HRRKVQPVDLDKARRRPRPWLS-----------SRA

RP1_BOVIN TPRGRHSITRLEELEDGQSYLCS-HGRKVQPVDLDKARRRPRPWLS-----------SRA

RP1chick TPKGRHSITCLDDLEDGKSYICS-HQRKMKPINLERASRKPLPWQI-----------SRP

RP1frog TPRGIHHVTSINELEDGKSYICS-HRKKIKPINLDRARKKPLLWQS-----------SRP

RP1fish TPHGVHAVHTLDELEDGKSYICS-DMRKVKPINLALVRRKLPPWYH-----------ARP

RP1zebfish TPRGTHAVCSLDDVQDGGSYLCS-DQKKVKPFNLDEVHKRQVPWNT-----------TRP

RP1L1_HUMA TPRGLHSLSALEQLEDGGCYLCS-DKKPPK----TPS--GPGRPQ------------ERN

RP1L1dog TPRGLHGLSALEQLEDGGYYLCS-DKKPPK----TPS--RPGWPQ------------GRS

RP1L1cow TPRGLHGLSTLEQLQDGGCYLCS-DKKPPR----TPG--GLGQPQ------------GRS

RP1L1_MOUS TPRGLHGLSALEQLQDGGCYLCS-DRKPPK----TSR--EPGRLQ------------RKS

RP1L1chick TPRGIHCISELDQLEDGGCYLCS-DKKYVKPINITTAGHRPGPPRN-----------GRP

DCX_MOUSE TIDGSRKIGSMDELEEGESYVCS-SDNFFKKVEYTKNVNPNWS---------------VN

DCX_HUMAN TIDGSRKIGSMDELEEGESYVCS-SDNFFKKVEYTKNVNPNWS---------------VN

DCXpan TIDGSRKIGSMDELEEGESYVCS-SDNFFKKVEYTKNVNPNWS---------------VN

DCXdog TIDGSRKIGSMDELEEGESYVCS-SDNFFKKVEYTKNVNPNWS---------------VN

DCXcow TIDGSRKIGSMDELEEGESYVCS-SDNFFKKVEYTKNVNPNWS---------------VN

DCXrat TIDGSRKIGSMDELEEGESYVCS-SDNFFKKVEYTKNVNPNWS---------------VN

DCXchick TIDGSRKIGSMDELEEGESYVCS-SDNFFKKVEYTKNVNPNWS---------------VN

DCXopp TIDGGRKIGSMDELEEGDSYVCS-SDTFFKKVEYAKNANPNWS---------------VN

DCXfish TIDGMNKITSLDELEEGESYVCA-SENFYKKVDYTKNVNPNWS---------------VN

DCLKpan TIDGLKKISSLDQLVEGESYVCG-SIEPFKKLEYTKNVNPNWS---------------VN

DCLKcow TIDGLKKISSLDQLLEGESYVCG-SIEPFKKLEYTKNVNPNWS---------------VN

DCLK_HUMAN TIDGLKKISSLDQLVEGESYVCG-SIEPFKKLEYTKNVNPNWS---------------VN

DCLKdog TIDGLKKISSLDQLVEGESYVCG-SIEPFKKLEYTKNVNPNWS---------------VN

DCLKadog TIDGLKKISSLDQLVEGESYVCG-SIEPFKKLEYTKNVNPNWS---------------VN

DCLK_MOUSE TIDGLKKISSLDQLVEGESYVCG-SIEPFKKLEYTKNVNPNWS---------------VN

DCLKrat TIDGLKKISSLDQLVEGESYVCG-SIEPFKKLEYTKNVNPNWS---------------VN

DCLKopp TIDGLKKISTLDQLVEGESYVCG-SIEPFKKLEYTKNVNPNWS---------------VN

DCLKchick TIDGSKKISSLDQLVEGESYVCG-SIEPFKKLEYTKNVNPNWS---------------VN

DCLK2zebfi TADGGKKITSLDDLVEGESYVCA-SNEPFRKVDYTKNVNPNWS---------------VN

DCLKafish TLDGTKKIGAIDQLVEGDSYVCS-SNEAYKKLDYTKNVNPNWS---------------VN

DCXzebfish SIDGNKKITNIDQLVEGESYVCG-STEAFKRVDYSNNVNPNWS---------------VN

DCLKfish SVDGQTKITSIEQLVEGESYVCA-SIEPYKKVDYTKNVNPNWS---------------VS

DCLK2_MOUS TIDGSRKVTSLDELLEGESYVCA-SNEPFRKVDYTKNVNPNW-----------------S

DCLK2rat TVDGSRKVTSLDELLEGESYVCA-SNEPFRKVDYTKNVNPNW-----------------S

DCLK2_HUMA TIDGSRKVTSLDELLEGESYVCA-SNEPFRKVDYTKNINPNW-----------------S

DCLK2pan TIDGSRKVTSLDELLEGESYVCA-SNEPFRKVDYTKNINPNW-----------------S

DCLK2dog TIDGSRKVTSLDELLEGESYVCA-SNEPFRKVDYTKNINPNW-----------------S

DCLK2cow TIDGSRKVTSLDELLEGESYVCA-SNEPFRKVDYTKNVNPNW-----------------S

DCLK2opp TIDGSRKLTSLEELMEG-------------------------------------------

DCLK2chick TIDGSKKLSSLDELLEGESYVCA-SNEPYRKVDYTKNVNPNW-----------------C

DCLK2fish SVDGSKKIGSMDELVEGESYVCA-SNEPYKKLEYTKISIPSWKPGAAPGAAAASRPPTTS

cio-Sca_14 SLDGARKIETIQELEHGESYVCA-SSDMFKRLEY--------------------------

A.gambiae TLEG-KKIEKVDDLEDGKCYVCSCNNEGFKRIDYNVSN---TN-------------TKNP

D.melanoga NLCG-KKITSLDELEDGQSYVCSCNNENFKKVEYNTGSQPLSN-------------LPLS

A.mellifer TMDG-KKVQSINDLEDGKCYVVSGQGEIFKKVEYSSTK------------------VRRG

ZYG-8 TIDGAQRITSVDQFEDGGGYVCS-STDAFKPVDYSRAAEPSWR-------------LTLA

cbZYG-8 TMDGTQRITSVDQFEDGGGYVCS-STDVFKPVDYSRAAEPSWR-------------LTLA

cio-Sca_10 TPIGGTLIEDIEELKDGESYVCA-SFEKFQRIKY--------------------------

DdDCX LASSGKLVKTMEEIIDGEYYVCA-GGETLNPLDFS-------------------------

DCDC2_MOUS TPRTGHRIRKLDQIESGGNYVAG-GPEAFKKLNYLDIGEIKKRPMEAVNT-------EVK

DCDC2rat TPRTGHRIRKLDQIESGGNYVAG-GQEAFKKL-YLDIGEIKKRPMEAVNT-------EVK

DCDC2_HUMA TPRTGHRIRKLDQIQSGGNYVAG-GQEAFKKLNYLDIGEIKKRPMEVVNT-------EVK

DCDC2pan TPRTGHRIRKLDQIQSGGNYVAG-GQEAFKKLNYLDIGEIKKRPMEVVNT-------EVK

DCDC2dog TPRTGHRIRKLDQLQSGGNYVAG-GQEAFKKLNYLDIGEMKKRPMEVINT-------EVK

DCDC2opp TPRAGHRIRKLDQIQSGGNYVAG-GLEAFKKL--------------------------VK

DCDC2cow TPRTGHRIWRLDQIQSGGNYVAG-GQESFKKLNYLDIGEIKKRPMEAVRT-------EVK

DCDC2frog TPRNGHRVTSLDDLQPGEFYVAG-GRENFKKLDYLHIGEIKRKTVDPLS--------QVK

DCDC2fish TPRGGHKVNSMENLKSGEQYVAA-GREKFKKLDYLEIGSRRKRMLHP--A-------QVK

h.roretzi TPNAGHRVNELHEINNNMVIVAG-GAERFRKLHYQNITPLPRKPMPPKHSYS-----TIR

cio-Sca_55 TPNLGHRVRDLTQLQNGMDLVAG-GVERFRKIQYK-------------------------

DCDC2Bzebf TPRYGHRVADLGDLQQGAQYVAA-GSERFKKLDY--------------------------

DCDC2Bcow TP-YGHPVTDLADLQNGGLYVAA-GFEHFHKLPYVMGPLDGSYLLVP-------------

: : :. .

RP1_MOUSE ISTH-------------------------VQLCPA----TANMSTMAPGMLRA--PRRLV

RP1rat ISTH-------------------------VKLRSA----TATMPTTAPGLFRA--PRRLV

RP1_HUMAN ISAH-------------------------SPPHP--------VAVAAPGMPRP--PRSLV

RP1pan ISAH-------------------------SPPHP--------VAVAAPGVPRP--PRSLV

RP1dog ISAH-------------------------AQRSPP----TSIGAAGAPGMLRA--PRRLL

RP1_BOVIN LSTH-------------------------VQRGP---------APAAPGMLRA--PRRLV

RP1chick ISARR----------------------RAVQLAKE----NEDGFGHRESKITT--PKKML

RP1frog ISARR----------------------LAVQLAQQ----NEVVPLQRKNTIVLGSSKKII

RP1fish VSS----------------------RRRTVHQSR---SFPGQKIHRKE-RAVLRTPKRLV

RP1zebfish VSAGRQA------------------RRELVRQLAKRNQVSTRTIKMSENTVVVRTPKRLT

RP1L1_HUMA PTAQQ------------------------LRDVEG----QREAPGTSSSRKSLKTPRRIL

RP1L1dog SSAQQ------------------------SRDFES----RCEAPGTSSSCKGPKAPRRIV

RP1L1cow PSAQQ------------------------LREF--------EAPGTASTCKGLKASRRIT

RP1L1_MOUS PSAGQ------------------------AQVFQG----GHEAPETSYSWKGPVAPRRLT

RP1L1chick SSTLR------------------------RAAQEG----RLDDYSTPFTQHGPRIPKKIT

DCX_MOUSE VKTSA------------------------NMKAPQSLASS--NSAQARENKDFVRPKLVT

DCX_HUMAN VKTSA------------------------NMKAPQSLASS--NSAQARENKDFVRPKLVT

DCXpan VKTSA------------------------NMKAPQSLASS--NSAQARENKDFVRPKLVT

DCXdog VKTSA------------------------NMKAPQSLASS--NSAQARENKDFVRPKLVT

DCXcow VKTSA------------------------NMKAPQSLASS--NSAQARENKDFVRPKLVT

DCXrat VKTSA------------------------NMKAPQSLASS--NSAQARENKDFVRPKLVT

DCXchick VKTSA------------------------NQKAPQSLASS--NSAQAKENKDFVRPKLVT

DCXopp VKTSA------------------------NLKAPQSLASS--HSAQARENKDFVRPKLVT

DCXfish VKASA------------------------SQKNMQSLAAK--AASEAREGKDFVRPKLVT

DCLKpan VKTTS------------------------ASRAVSSLATAKGSPSEVRENKDFIRPKLVT

DCLKcow VKTTS------------------------ASRAVSSLATAKGSPSEVRENKDFIRPKLVT

DCLK_HUMAN VKTTS------------------------ASRAVSSLATAKGSPSEVRENKDFIRPKLVT

DCLKdog VKTTS------------------------ASRAVSSLATAKGSPSEVRENKDFIRPKLVT

DCLKadog VKTTS------------------------ASRAVSSLATAKGSPSEVRENKDFIRPKLVT

DCLK_MOUSE VKTTS------------------------ASRAVSSLATAKGGPSEVRENKDFIRPKLVT

DCLKrat VKTTS------------------------ASRAVSSLATAKGGPSEVRENKDFIRPKLVT

DCLKopp VKTTS------------------------ASRTVSSLATAKGSPSDVRENKDFIRPKLVT

DCLKchick VKTTS------------------------TSRSVPSLATAKGGTPDTKENKDFIRPKLVT

DCLK2zebfi VKTG-------------------------ASRSMPSLTATKNELRE-RESKDYIKPKLVT

DCLKafish VKASPT-----------------------SSRGPPSLGNTNAVVPENRETKDFIRPKLVT

DCXzebfish VRALG------------------------SSKCPTSLASS-KSGPQFRESKDFIRPKLVT

DCLKfish ARTAV------------------------PTRDPSSLGSAKSACGEGRDNRDFIKPKLVT

DCLK2_MOUS VNIKGG-----------------------TTRTLAVAS-AKSEVKES---KDFIKPKLVT

DCLK2rat VNIKGG-----------------------TTRTLAVAS-AKSEVKES---KDFIKPKLVT

DCLK2_HUMA VNIKGG-----------------------TSRALAAASSVKSEVKES---KDFIKPKLVT

DCLK2pan VNIKGG-----------------------TSRALAAASSVKSEVKES---KDFIKPKLVT

DCLK2dog VNIKGG-----------------------TTRALAAPSSVKSEVKES---KDFIKPKLVT

DCLK2cow VNIKGG-----------------------ATRS-APPSSVKSEVKES---KDFIKPKLVT

DCLK2opp ----------------------------------------KSEGKES---XDFIKPKLVT

DCLK2chick VNIRTG-----------------------STRSLTSLTSTKSEVKES---KDFIKPKLVT

DCLK2fish TGVSAG-----------------------ATVAPAAVSRDRPESRESRENKDFIKPKLVT

cio-Sca_14 -----------------------------------------------------------T

A.gambiae NRLSRTERNLMLFAACFFFCGRLIR--PLSPVKNGGSNGS----TPLKEIDSVVHPRIVT

D.melanoga NSRSNSHRLAKCRPSSPLKNGLLAGSSPFPACGGGTGNGSPLIASRLSDRVTVVHPRIVT

A.mellifer SSLSG---------------------LPQSPAGTG---------RQISAIPLCVKAKIIT

ZYG-8 NRYNR----------------------HLETKKLALS-----VVEPCHENTDFVFPRIIK

cbZYG-8 NRYNR----------------------HLETKKLALN-----VVEPANENTDFVIPKIIK

cio-Sca_10 -----------------------------------------------------MKPKVVT

DdDCX -------------------------------------------------------PTLSE

DCDC2_MOUS PVIH------------------------------------SRINVSARFRKSLHEPCTIF

DCDC2rat PVIH------------------------------------SKINVSARFRKALHEPCTI-

DCDC2_HUMA PVIH------------------------------------SRINVSARFRKPLQEPCTIF

DCDC2pan PVIH------------------------------------SRINVSARFRKPLQEPCTIF

DCDC2dog PVMH------------------------------------SRINVSARFRKPLQEPCTIF

DCDC2opp PVIH------------------------------------SRINVSARFKKPLQEPCTI-

DCDC2cow PVTH------------------------------------SRINVSSRFRKPLLEPCTIF

DCDC2frog PVSH------------------------------------SRINVSARFRKNVQEPCTVF

DCDC2fish PPPQ------------------------------------NRFIVSARFLKPIKEPCAVF

h.roretzi PVYHN-----------------------------------SKMNVPARWKQLVQGPCSIY

cio-Sca_55 ------------------------------------------------------EPCQIY

DCDC2Bzebf ------------------------------------------------------------

DCDC2Bcow PAISS-----------------------------------KCPCWGGGGIDFCFSEAKEN

RP1_MOUSE VFRNG--DPKNKHVVLLSRRITQSFEAFLQYLTQVM------QCPVAKLYATDGRK----

RP1rat VFRNG--DPKTRRVVLLSRRITQSFEAFLQYLTQVM------QYPVAKLYATDGRK----

RP1_HUMAN VFRNG--DPKTRRAVLLSRRVTQSFEAFLQHLTEVM------QRPVVKLYATDGRR----

RP1pan VFRNG--DPKTRRAVLLSRRVTQSFEAFLQHLTEVM------QRPVVKLYATDGRR----

RP1dog VFRNG--DPKIRRVVIVNRRVTQSFQAFLQHLTEVM------RFPVTKLYATDGRK----

RP1_BOVIN VFRNG--DPKTRRAIVLNRRVTQSFEVFLQYLTQVM------QRPVTKLYATDGRK----

RP1chick VFKNG--DVRLRRTIVLGKKNTQTFEAFLDYMSELM------QYPVAKLYTTDGRK----

RP1frog IFKNG--DLGFKHHFNLNKKTKQSFDSFLDQVAEAL------QYPVFKLYSSDGRR----

RP1fish VFCNG--VPAVHHTLVLDKRITPTFETILEYISEVV------QFHVVKLHMLDGRR----

RP1zebfish VYKNR--DPSMKRVIVLHRRIAPTFEALLDYLSQMM------QFPVVKLYTEDGRR----

RP1L1_HUMA LIKNM--DPRLQQTVVLSHRNTRNLAAFLGKASDLL------RFPVKQLYTTSGKK----

RP1L1dog LVKNG--DPRFQQTVVLSHRNTRNLMAFLSKASDLL------HFPVKQVYTTSGKK----

RP1L1cow LVKNG--DPQLQQTVVLSHRNTRNLTAFLSKASDLL------RFPVKHVYTTGGKR----

RP1L1_MOUS LVKNG--DPRRQQTVVLSHKNTRSLAAFLGKASELL------RFPVKQVYTTRGKK----

RP1L1chick LVKNG--ETSFRRSIILNRRNARSFKTLLDEISEIL------QFPVKKLFTVDGKK----

DCX_MOUSE IIRSG-VKPRKAVRVLLNKKTAHSFEQVLTDITEAI---KLETGVVKKLYTLDGKQ----

DCX_HUMAN IIRSG-VKPRKAVRVLLNKKTAHSFEQVLTDITEAI---KLETGVVKKLYTLDGKQ----

DCXpan IIRSG-VKPRKAVRVLLNKKTAHSFEQVLTDITEAI---KLETGVVKKLYTLDGKQ----

DCXdog IIRSG-VKPRKAVRVLLNKKTAHSFEQVLTDITEAI---KLETGVVKKLYTLDGKQ----

DCXcow IIRSG-VKPRKAVRVLLNKKTAHSFEQVLTDITEAI---KLETGVVKKLYTLDGKQ----

DCXrat IIRSG-VKPRKAVRVLLNKKTAHSFEQVLTDITEAI---KLETGVVKKLYTLDGKQ----

DCXchick IIRSG-VKPRKAVRVLLNKKTAHSFEQVLTDITEAI---KLETGVVKKLYTLDGKQ----

DCXopp IIRSG-VKPRKAVRVLLNKKTAHSFEQVLTDITEAI---KLETGVVKKLYTLDGKQ----

DCXfish VMRSG-VKPRKAVRVLLNKKTAHSFEQVLTDITEAI---KLESGVVKRIYTLDGKQ----

DCLKpan IIRSG-VKPRKAVRILLNKKTAHSFEQVLTDITDAI---KLDSGVVKRLYTLDGKQ----

DCLKcow IIRSG-VKPRKAVRILLNKKTAHSFEQVLTDITDAI---KLDSGVVKRLYTLDGKQ----

DCLK_HUMAN IIRSG-VKPRKAVRILLNKKTAHSFEQVLTDITDAI---KLDSGVVKRLYTLDGKQ----

DCLKdog IIRSG-VKPRKAVRILLNKKTAHSFEQVLTDITDAI---KLDSGVVKRLYTLDGKQ----

DCLKadog IIRSG-VKPRKAVRILLNKKTAHSFEQVLTDITDAI---KLDSGVVKRLYTLDGKQ----

DCLK_MOUSE IIRSG-VKPRKAVRILLNKKTAHSFEQVLTDITDAI---KLDSGVVKRLYTLDGKQ----

DCLKrat IIRSG-VKPRKAVRILLNKKTAHSFEQVLTDITDAI---KLDSGVVKRLYTLDGKQ----

DCLKopp IIRSG-VKPRKAVRILLNKKTAHSFEQVLTDITDAI---KLDSGVVKRLYTLDGKQ----

DCLKchick IIRSG-VKPRKAVRILLNKKTAHSFEQVLTDITDAI---KLDSGVVKRLYTLDGKQIFLA

DCLK2zebfi VIRSG-VKPRKAVRILLNKKTAHSFEQVLTDITDAI---KLDSGAVKRLYTLEGKQ----

DCLKafish VVRSG-VKPRKAVRILLNKKTAHSYEQVLTDITDAI---KLDSGVVKKIYTLEGKL----

DCXzebfish VIRSG-VKPRKAVRILLNKKTAHSFEQVLTDITDAI---KLDSGIVKRIY----------

DCLKfish IIRSG-VKPRKAVRVLLNKKTAHSFDQVLTDITDAI---KLDSGVVRRLYTVDGKM----

DCLK2_MOUS VIRSG-VKPRKAVRILLNKKTAHSFEQVLTDITEAI---KLDSGVVKRLCTLDGKQ----

DCLK2rat VIRSG-VKPRKAVRILLNKKTAHSFEQVLTDITEAI---KLDSGVVKRLCTLDGKQ----

DCLK2_HUMA VIRSG-VKPRKAVRILLNKKTAHSFEQVLTDITEAI---KLDSGVVKRLCTLDGKQ----

DCLK2pan VIRSG-VKPRKAVRILLNKKTAHSFEQVLTDITEAI---KLDSGVVKRLCTLDGKQVRSH

DCLK2dog VIRSG-VKPRKAVRILLNKKTAHSFEQVLTDITEAI---KLDSGVVKRLCTLDGKQ----

DCLK2cow VIRSG-VKPRKAVRILLNKKTAHSFEQVLTDITEAI---KLDSGVVKRLCTLDGKQ----

DCLK2opp VIRSG-VKPRKAVRILLNKKTAHSFEQVLTDITEAI---KLDSGVVKRLCTLDGKQ----

DCLK2chick VIRSG-VKPRKAVRILLNKKTAHSFEQVLTDITEAI---KLDSGVVKRLCTLDRKQ----

DCLK2fish VIRSG-VKPRKAVRILLNKKTAHSFEQVLADITEAI---KLDSGAVKRLYTLDGKQ----

cio-Sca_14 IIRSG-VKPRKAVRILLNKKTAHSLEQVLNDVTKAI---KLDTGAVRKVYTLQGKQ----

A.gambiae LIRNG-VKPRKILRLLLNKRNSPTYEHVLTAITQCV---KLDTGCVRKVFTVAGVP----

D.melanoga LIRSG-TKSRRIMRLLLNKRNSPSFDHVLTAITQVV---RLDTGYVRKVFTLSGIP----

A.mellifer LIRHG-TKPRKVVRLLLNKRNAPSLEHALEAITEAV---KLDSGAVRKVYTLSGQQ----

ZYG-8 VIRNG-VKPRRISRHLLNKKTARSFDQVLRDLTFVV---KLDSGAIRKLFTLSGRP----

cbZYG-8 VIRNG-VKPRRISRHLLNKKTARSFDQVLRDLTLIV---KLDAGAIRKLFTLSGRP----

cio-Sca_10 VVRAGQIRPHKKITILLNRRAVQTYEQLVSDISEALGQPKWKNDHIRRLYTLKGRE----

DdDCX HVKQK----------KLQEQQQQASEQQKPQEQEIF------------------------

DCDC2_MOUS LIANG-DLISPASRLLIPKKALNQWDHVLQMVTEKIT--LRS-GAVHRLYTLEGKL----

DCDC2rat LIANG-DLISPASRLLIPRKALNQWDHVLQMVTEKIT--LRS-GAVHRLYTLEGKL----

DCDC2_HUMA LIANG-DLINPASRLLIPRKTLNQWDHVLQMVTEKIT--LRS-GAVHRLYTLEGKL----

DCDC2pan LIANG-DLINPASRLLIPRKTLNQWDHVLQMVTEKIT--LRS-GAVHRLYTLEGKL----

DCDC2dog LIANG-DLISPASRLLIPRKTLNQWDHVLQMVTEKIT--LRT-GAVHRLYTLEGKP----

DCDC2opp LIANG-DLISPASRLFIPRKALNQWDLVLEMVTEKIT--LRS-GAVHRLYTLEGKL----

DCDC2cow LIANG-DLINPASRLLIPRKALNQWDHVLQMITEKIT--LRS-GAVHRLYTLEGKL----

DCDC2frog LVANG-DTLNPFIRLLIPRKTLEQWELVLALVTEKVK--LRN-GAVHRLYTLEGTP----

DCDC2fish VVANG-DVLNSAVRLLIHQRMLGQFDKILEMITEKMG--LRVLGGVRSLYTYDGTQ----

h.roretzi VYGNR-DINGVAIKMLLSARIMKNWDMVLEEITEKIS--IRTGQAVRRLYTLDGVL----

cio-Sca_55 VYGNG-DINAPAIRLLLIPRAMKSWDLVLSEITEKIC--LRTGKAVRKLYDMDYHL----

DCDC2Bzebf ------------------------------------------------------------

DCDC2Bcow VFRNG-DLLSPPFSLKLSQAASEDWEAVLKLLKEKVK--LQS-GAVRKGRGPSGPA----

RP1_MOUSE --------------------------------------VPSLQAVILSSGAVVAAG-REP

RP1rat --------------------------------------VPSLQAVILSSGAVVAAG-REP

RP1_HUMAN --------------------------------------VPSLQAVILSSGAVVAAG-REP

RP1pan --------------------------------------VPSLQAVILSSGAVVAAG-REP

RP1dog --------------------------------------VPSLQAVILSSGAVVAAG-REP

RP1_BOVIN --------------------------------------VPSLQAVILSSGAVVAAG-REP

RP1chick --------------------------------------VPNLQALILCSGAIVAAG-REP

RP1frog --------------------------------------ILSIRALLLSSGTVVAAG-RES

RP1fish --------------------------------------VDGLPGLILCSGVVVAAG-REP

RP1zebfish --------------------------------------IEGLSALILCTGIVVAAG-NEP

RP1L1_HUMA --------------------------------------VDSLQALLHSPSVLVCAG-HEA

RP1L1dog --------------------------------------VDSLKGLLHSPSVLVCAG-YES

RP1L1cow --------------------------------------VDSLKALLRSPSVLVCAG-LEP

RP1L1_MOUS --------------------------------------VDSLQTLLDGPSVLVCAG-NEA

RP1L1chick --------------------------------------IDSMQALLHCPNVLVCVG-REP

DCX_MOUSE --------------------------------------VTCLHDFFGDDDVFIACG-PEK

DCX_HUMAN --------------------------------------VTCLHDFFGDDDVFIACG-PEK

DCXpan --------------------------------------VTCLHDFFGDDDVFIACG-PEK

DCXdog --------------------------------------VTCLHDFFGDDDVFIACG-PEK

DCXcow --------------------------------------VTCLHDFFGDDDVFIACG-PEK

DCXrat --------------------------------------VTCLHDFFGDDDVFIACG-PEK

DCXchick --------------------------------------VTCLHDFFGDDDVFIACG-PEK

DCXopp --------------------------------------VTCLHDFFGDDDVFIACG-PEK

DCXfish --------------------------------------VTCLQDFFGDDDVFIACG-PEK

DCLKpan ------------------------------------------------------------

DCLKcow ------------------------------------------------------------

DCLK_HUMAN --------------------------------------VMCLQDFFGDDDIFIACG-PEK

DCLKdog --------------------------------------VMCLQDFFGDDDIFIACG-PEK

DCLKadog --------------------------------------VMCLQDFFGDDDIFIACG-PEK

DCLK_MOUSE --------------------------------------VMCLQDFFGDDDIFIACG-PEK

DCLKrat --------------------------------------VMCLQDFFGDDDIFIACG-PEK

DCLKopp --------------------------------------VMCLQDFFGDDDIFIACG-PEK

DCLKchick FRLIAAVDARAFWHTSGKHDMNFIAPPLPVAAGWEVVMVMCLQDFFGDDDIFIACG-PEK

DCLK2zebfi ------------------------------------------------------------

DCLKafish --------------------------------------VSCLQDFFGDEDVFVACG-PEK

DCXzebfish ------------------------------------------------------------

DCLKfish --------------------------------------VTCLQDFFAEDDIFFACG-PEK

DCLK2_MOUS --------------------------------------VTCLQDFFGDDDVFIACG-PEK

DCLK2rat --------------------------------------VTCLQDFFGDDDVFIACG-PEK

DCLK2_HUMA --------------------------------------VTCLQDFFGDDDVFIACG-PEK

DCLK2pan FGVSSCFRKVEVALPLGIGEA------------LSLLSVTCLQDFFGDDDVFIACG-PEK

DCLK2dog --------------------------------------VTCLQDFFGDDDVFIACG-PEK

DCLK2cow --------------------------------------VTCLQDFFGDDDVFIACG-PEK

DCLK2opp --------------------------------------VTCLQDFFGDDDVFIACG-PEK

DCLK2chick --------------------------------------VTCLQDFFGDDDVFIACG-PEK

DCLK2fish --------------------------------------LTCLQDFFGDDDVFMACG-PEK

cio-Sca_14 --------------------------------------VQSLQDFFGNDDIFIAYG-HEK

A.gambiae --------------------------------------VQRLAQFFEEDDVFFAYG-NER

D.melanoga --------------------------------------VVRLSDFFGSDDVFFAYG-TER

A.mellifer --------------------------------------VTSLEQFFENDDIFVAYG-PEK

ZYG-8 --------------------------------------VLSLQDFFRDDDVFVAYGGNEK

cbZYG-8 --------------------------------------VLTLQDFFREDDVFVAYGGNDK

cio-Sca_10 --------------------------------------IRSVSDFFREDDVFIAVG-REQ

DdDCX ------------------------------------------------------------

DCDC2_MOUS ---------------------------------------VESGAELENGQFYVAVG-RDK

DCDC2rat ---------------------------------------VESGAELENGQFYVAVG-RDK

DCDC2_HUMA ---------------------------------------VESGAELENGQFYVAVG-RDK

DCDC2pan ---------------------------------------VESGAELENGQFYVAVG-RDK

DCDC2dog ---------------------------------------VESGAELENGQFYVAVG-RDK

DCDC2opp ---------------------------------------VQSGSELENGQFYVAVG-RDK

DCDC2cow ---------------------------------------VESGAELENGQFYVAVG-RDR

DCDC2frog ---------------------------------------IQNGLELENGQFYVAVG-REK

DCDC2fish ---------------------------------------VNDGNQLESGQLYVAVG-RER

h.roretzi ---------------------------------------IQGSENLENGRYYVAVG-YER

cio-Sca_55 ---------------------------------------LGDPSELENGKYYIAVG-TER

DCDC2Bzebf ------------------------------------------------------------

DCDC2Bcow ---------------------------------------LG--ARVEPGGLALVPG-ELL

RP1_MOUSE FKPGNYD------

RP1rat FK-----------

RP1_HUMAN FK-----------

RP1pan FK-----------

RP1dog FK-----------

RP1_BOVIN FK-----------

RP1chick FK-----------

RP1frog FIYANYE------

RP1fish FRAA---------

RP1zebfish FR-----------

RP1L1_HUMA FR-----------

RP1L1dog FK-----------

RP1L1cow FRPLVTED-----

RP1L1_MOUS FR-----------

RP1L1chick FK-----------

DCX_MOUSE FR------YAQDD

DCX_HUMAN FR------YAQDD

DCXpan FR------YAQDD

DCXdog FR------YAQDD

DCXcow FR------YAQDD

DCXrat FR------YAQDD

DCXchick FR------YAQDD

DCXopp FR------YAQDD

DCXfish FR------YAQDD

DCLKpan -------------

DCLKcow -------------

DCLK_HUMAN FR------YQDD-

DCLKdog FR------YQDD-

DCLKadog FR------YQDD-

DCLK_MOUSE FR------YQDD-

DCLKrat FR------Y----

DCLKopp FR------YQDD-

DCLKchick FR------YQDD-

DCLK2zebfi -------------

DCLKafish FR------YQDDL

DCXzebfish -------------

DCLKfish FR------YQDD-

DCLK2_MOUS YR------YAQDD

DCLK2rat YR------YAQDD

DCLK2_HUMA FR------YAQDD

DCLK2pan FR------YAQDD

DCLK2dog FR------YAQDD

DCLK2cow FR------YAQDD

DCLK2opp FR------YAQDD

DCLK2chick YR------YAQDD

DCLK2fish FR------YAQDD

cio-Sca_14 LS------QDD--

A.gambiae VGNNDFELEAEE-

D.melanoga IN------TAED-

A.mellifer -------------

ZYG-8 MA-------ADD-

cbZYG-8 MA-------ADD-

cio-Sca_10 -------------

DdDCX -------------

DCDC2_MOUS FKRLPYSE-----

DCDC2rat FK-----------

DCDC2_HUMA FK-----------

DCDC2pan FK-----------

DCDC2dog FK-----------

DCDC2opp FK-----------

DCDC2cow FKKLPYSE-----

DCDC2frog FKKLPYS------

DCDC2fish FKKLPYI------

h.roretzi FKRAPYG------

cio-Sca_55 IKKIAY-------

DCDC2Bzebf -------------

DCDC2Bcow PSRARHLGRAV--

Proteins alignment N-terminal domains

CLUSTAL W (1.83) multiple sequence alignment

DCDC2_MO-N -SQPVVKSVLVYRNGDPFFAGRRVVIHE-KKVSSFDVFLKEVTG-----GVQAPFG---A

DCDC2cow-N -SQPVVKSVLVYRNGDPFFTGRRVVFHE-KKVSSFDVFLKEVTG-----GVQAPFG---A

DCDC2dog-N -SQPVVKSVLVYRNGDPFFAGRRVVIHE-KKVSSFDVFLKEVTG-----GVQAPFG---A

DCDC2_HU-N ------KSVLVYRNGDPFYAGRRVVIHE-KKVSSFEVFLKEVTG-----GVQAPFG---A

DCDC2pan-N ------KSVLVYRNGDPFYAGRRVVIHE-KKVSSFEVFLKEVTG-----GVQAPFG---A

DCDC2opp-N ------KSVLVYRNGDPFFAGRRVVIHE-KKVSSFDVFLKEVTG-----GVQAPFG---A

DCDC2rat-N ------KSVLVYRNGDPFFAGRRVVIHE-KKVSSFDIFLKEVTG-----GVQAPFG---A

DCDC2fro-N -QQPAVKTVHVFRNGDPFYRGRRMLIHE-RRVGTFDVFLKDVTG-----GVQAPFG---A

DCDC2fis-N ------KNIFMFRNGDPYYEARRIVINQ-KRVSNFETLLREVTG-----GIQAPFG---A

H.roretz-N -----AKNVHVYVNGDQYYPGRKFVVNR-RYICDFDGFLNLVTS-----GLKPSFG---A

cio-Sca_55_N -----AKNVHVYLNGDRFFPGRKFVVNR-RHISDFDGFLNQVTV-----GMKAPFG---A

DCDC2B_HU-N -----AKRVVVYRNGDPFFPGSQLVVTQ-RRFPTMEAFLCEVTS-----AVQAPL----A

DCDC2Bpa-N -----AKRVVVYRNGDPFFPGSQLVVTQ-RRFPTMEAFLCEVTS-----AVQAPL----A

DCDC2Bco-N -GSPAAKRVVVYQNGDPFSPGRQLVVTQ-RRFPTLETFLCEVTS-----AVRAPV----A

DCDC2Bdog -----AKRVVIYRNGDPYFPGHQLVVSQ-RRFPTLETFLQEVTS-----IVQAPV----A

DCDC2Bdo-N -GNPAAKRVVIYRNGDPYFPGHQLVVSQ-RRFPTLETFLQEVTS-----IVQAPV----A

DCDC2B_MOU -----AKRILVYRNGDAFFPGHQLVVTQ-RRYPTMEALLYEVTS-----AVQAPL----A

DCDC2Bze-N ------KSVMVYRNGDPFFSGRRFVVNQ-RQIATMDALLNDITL-----NIGAPL----A

tetCAG1045 ------KSVVVYKNGDPFYTGRRFVVNQ-RQVATMEAFLNEVTQ-----SIGAPL----A

DCDC2Ach-N -LAHPAKNVVVYRNGDPFFHGRKFVVNQ-RQFLTFEAFLNEVTK-----SIHAPL----A

DCDC2A_MO -----AKTILVYRNGDQFYVGRKFVFSR-RRVANFEALLEQLTE-----QVEVPF----G

DCDC2Arat -----AKTILVYRNGDQFYVGRKFVFSR-RRVANFEALLEQLTE-----QVEVPF----G

DCDC2Ac-NC ------KTIVVYRNGDQFYVGRKFVLSR-RRVATFEALLEQLTE-----QVEVPF----G

DCDC2A_HUM -----AKTIVVYRNGDPFYVGKKFVLSR-RRAATFEALLEQLTE-----QVDVPF----G

DCDC2Apan -----AKTIVVYRNGDPFYVGKKFVLSR-SRAATFEALLEQLTE-----QVDVPF----G

DCDC2Adog -RPAPAKAIVVYRNGDAFLEGRRCVLSR-RRAATFEALLDQLTE-----QVEVPF----G

DCDC2Aopp -----ARTILVYRNGDEFYVGTKFVINR-KRVPNIEALMTQLND-----KLAVPF----G

RP1_MOU-N -HPVVAKRISFYKSGDPQFGGVRVVVNP-RSFKTFDALLDSLSR-----KVPLPF----G

RP1rat-N -----AKRISFYKSGDPQFGGVRVVVNP-RSFKTFDALLDNLSR-----KVPLPF----G

RP1_HUM-N -----AKRISFYKSGDPQFGGVRVVVNP-RSFKSFDALLDNLSR-----KVPLPF----G

RP1pan-N -----AKRISFYKSGDPQFGGVRVVVNP-RSFKSFDALLDNLSR-----KVPLPF----G

RP1_BOVIN-N -----AKRISFYKSGDPQFGGVRVVLNP-RSFKTFDALLDNLSG-----KVPLPF----G

RP1dog-N -----AKKISFYKSGDPQFGGVKVVVNP-RSFKTFDALLDNLSR-----KVPLPF----G

RP1frog-N -EPASTKRVCFYKSGDPQFNGIKMVVSN-RSFKTFDALLDTLSK-----KVPLPF----G

RP1opp-NC -----AKRISFYKSGDPQFNGIQMVVNP-RSFKSFDALLDNLSK-----KVPLPF----G

RP1chick-N -EPVVAKRICFYKSGDPQFNGIKMVINN-RSYKTFDALLDSLSK-----RVPLPF----G

RP1zebfi-N ------KRVCFYKSGDPQFTGHRMVINS-RTFKTFDALLDALSK-----KVPLPF----G

RP1fish-N -----SKRVCFYKSGDAQFSGLRMVINN-RTFNTFDALLDSLSR-----KVPLPF----G

RP1L1_MO-N -----AKKITFLKRGDPQFAGVRLAVHQ-RTFKTFSSLMDELSQ-----RMPLSF----G

RP1L1cow-N -QVTPAKKITFLKRGDPQFAGVRVAVHQ-RAFRSFGALMDELSQ-----RVPLSF----G

RP1L1_HU-N -----AKKITFLKRGDPRFAGVRLAVHQ-RAFKTFSALMDELSQ-----RVPLSF----G

RP1L1opp-N -----AKKITFLKRGDPRFAGVRLAVHQ-RAFKTFSALMDELSQ-----RVPLSF----G

RP1L1dog ------KKITFLKRGDPRFAGVRLAVDQ-RAFKSFGALMDELSQ-----RVPLSF----G

RP1L1chi-N -QVPPAKKITFFKSGDPQFGGVKMAINQ-RSFKSFNALMDDLSH-----RVPLPF----G

anoEAL4118 ----KARRVLFYRNGDPFFPGVEFRFKPGRDICTLEALLDKISA-----RMDLPR----G

drNP_64872 ----KARRVVFYRNGDPFFPGVELRYRPGRDVTSLDNLLDKISP-----KMDLPR----G

A.mellif ----RARRVTFYKNGDPYFPGIEFRFKPGRDIGSLEALLDRLSL-----RMDLPR----G

DCX_MOU-N -NEKKAKKVRFYRNGDRYFKGIVYAVSS-DRFRSFDALLADLTR-SLSDNINLPQ----G

DCX_HUM-N -NEKKAKKVRFYRNGDRYFKGIVYAVSS-DRFRSFDALLADLTR-SLSDNINLPQ----G

DCXchick-N -NEKKAKKVRFYRNGDRYFKGIVYAVSS-DRFRSFDALLADLTR-SLSDNINLPQ----G

DCXdog-N -NEKKAKKVRFYRNGDRYFKGIVYAVSS-DRFRSFDALLADLTR-SLSDNINLPQ----G

DCXcow-N -NEKKAKKVRFYRNGDRYFKGIVYAVSS-DRFRSFDALLADLTR-SLSDNINLPQ----G

DCXrat-N -NEKKAKKVRFYRNGDRYFKGIVYAVSS-DRFRSFDALLADLTR-SLSDNINLPQ----G

DCXpan-N -NEKKAKKVRFYRNGDRYFKGIVYAVSS-DRFRSFDALLADLTR-SLSDNINLPQ----G

DCXopp-N -NEKKAKKVRFYRNGDRYFKGIVYAVST-DRFRSFDALLADLTR-SLSDNINLPQ----G

DCXfish-N -SEKKAKKVRFYRNGDRYFKGIVYAVAN-DRFRTFDSLLADLTR-SLSDHINLPQ----G

DCLKrat-N -SEKKAKKVRFYRNGDRYFKGIVYAISP-DRFRSFEALLADLTR-TLSDNVNLPQ----G

DCLKpan-N -SEKKAKKVRFYRNGDRYFKGIVYAISP-DRFRSFEALLADLTR-TLSDNVNLPQ----G

DCLKadog-N -SEKKAKKVRFYRNGDRYFKGIVYAISP-DRFRSFEALLADLTR-TLSDNVNLPQ----G

DCLKdog-N -SEKKAKKVRFYRNGDRYFKGIVYAISP-DRFRSFEALLADLTR-TLSDNVNLPQ----G

DCLK_HUM-N -SEKKAKKVRFYRNGDRYFKGIVYAISP-DRFRSFEALLADLTR-TLSDNVNLPQ----G

DCLK_MOU-N -SEKKAKKVRFYRNGDRYFKGIVYAISP-DRFRSFEALLADLTR-TLSDNVNLPQ----G

DCLKcow-N -SEKKAKKVRFYRNGDRYFKGIVYAISP-DRFRSFEALLADLTR-TLSDNVNLPQ----G

DCLKopp-N -SEKKAKKVRFYRNGDRYFKGIVYAISP-DRFRSFEALLADLTR-TLSDNVNLPQ----G

DCLKchi-N -SEKKAKKVRFYRNGDRYFKGIVYAISP-DRFRSFEALLADLTR-TLSDNVNLPQ----G

DCLKfish-N -SEKKAKKVRFYRNGDRYFKGIVYAISQ-ERFGSLEALLADLTR-SLSDNVNLPQ----G

DCLKafish-N SEEKRAKKVRFYRNGDRYFNGIVYAIST-DRFRTFDALLADLTR-SLSDNVNLPQ----G

DCLKzebfi-N -SEKKAKKVRFYRNGDRYFNGIVYAISS-DRIRTFDALLADLTR-TLSDNVNLPQ----G

DCLK2_HU-N -SEKKAKKARFYRNGDRYFKGLVFAISS-DRFRSFDALLIELTR-SLSDNVNLPQ----G

DCLK2pan-N -SEKKAKKARFYRNGDRYFKGLVFAISS-DRFRSFDALLIELTR-SLSDNVNLPQ----G

DCLK2rat-N -SEKKAKKARFYRNGDRYFKGLVFAISS-DRFRSFDALLIELTR-SLSDNVNLPQ----G

DCLK2_MO-N -SEKKAKKARFYRNGDRYFKGLVFAISN-DRFRSFDALLIELTR-SLSDNVNLPQ----G

DCLK2dog-N -SEKKAKKARFYRNGDRYFKGLLFAISG-DRFRSFDALLMELTR-SLSDNVNLPQ----G

DCLK2cow-N -SEKKAKKARFYRNGDRYFKGLVFAISS-DRFRSFDALLMELTR-SLSDNVNLPQ----G

DCLK2chi-N -SEKKARKARFYRNGDKYFKGLVYAISS-DRFRSFDALLAELTR-SLSDNVNLPQ----G

DCLK2opp-N -SEKKAKKARFYRNGDRYFKGLVYAISS-DRFRSFDALLIELTR-SLSDNVNLPQ----G

DCLK2zeb-N -SEKKAKKVRFYRNGDKYFKGLVYAVSG-DRFRSFDALLMELTR-SLSDNVNLPQ----G

DCLK2azebf -SEKRAKKVRFYRNGDRYFKGLVYAVSS-DRFRSMDALLAELTR-ALADNLHLPQ----G

DCLK2fis-N -SEKKAKKVRFYRNGDRYFKGLVYAVSS-DRFRSYDALLMELTR-SLSDNLYLPQ----G

cio-Sca_14_N -DEKRAKKVRFYRNGDNFHTGLVYAVST-TRFRTFEAMLIDLTN-RLADKVHLPH----G

anoXP_3197 ---KKAKRIRFFRNGDKFYPGSTIPVSV-ERYRSFDSLTEDLTR-LLEDSVTLTG----A

D.melano-N ---RKALRIKFYRNGDRFYPGITIPVSN-ERYRSFERLFEDLTR-LLEENVKIPG----A

A.mellif-N ---KKAKRVRFFRNGDKFYTGIVMAVTP-ERYRSFDSLATDLTR-ALISSVTLPN----G

drNP_65115 ---KKALRVCFLRNGDRHFKGVNLVISR-AHFKDFPALLQGVTE-SLKRHVLLRS----A

ZYG-8-N -HLLKAKRLRFYRNGDQYFKGIQYALQS-DRVKSMQPLMEDLMKTVICDSTALPH----G

cbZYG-8-N -HLVKAKRLRFYRNGDQYFKGIPYALQC-DRVKSMQPLMEELMKTVICDSTALPH----G

cio-Sca_10_N ---RRPRIVMFYKNGDRYFKGKSLHITP-HRYLHYEELLSDLSK-----SMSLPY----G

DdDCX-N ---EKAKVIMCFRNGDRYHSGERVTVHS-TKFKTYDQLKEQLSK-----QVKLPTG---P

ceNP_00102 ------RKISIYKNGDRYHRGVKFVINP-RVIKDMEPLLNQVND-----RIELSH----G

ceCAB04937 ------RKISIYKNGDRYHRGVKFVINP-RVIKDMEPLLNQVND-----RIELSH----G

cbCAE73316 ------RKISIYKNGDRFHRGVKFVINP-RVVKDMEPLLNQIND-----RIELSH----G

cbCAE73319 -NAAGVKRIHVWRNADVFFPGIQVVVNT-HRVPSIDVLLDVVSE-----RIGLIN----G

mBAC26042 ----RVKRVWAYQNG-GRHADGTYVQAG-----ALSELLDGCTV-----RLKMSH----P

BAC26042ra -------------------------------------LLDDCTA-----RLKMSH----P

DCDC1dog -SEKTSVRILFFKNGMGQDGHEITVGKE-----TIKKVLDTCTM-----KMKLNL----P

urcXP_7883 ------RRVMVFLNGDASESHEVVANLD-----QFNQFLDSCTS-----KLNLNC----P

DCDC1_HUMAN -------KVTAYKNGSRTVFARVTAP-------TITLLLEECTE-----KLNLNM----A

DCDC1pan -------KVTAFKNGSRTVFARVTVP-------TITLLLEKCTE-----KLNLNM----A

bovXP_8740 -------------------------------------LLEECTE-----KLNLNT----A

hFLJ-N ---QKAVKIIAYKNGDGYRNGKLIVAG------TFPMLLTECTE-----QLGLAR----A

FLJpan-NC ---QKAVKIIAYKNGDGYRNGKLIVAG------TFPMLLTECTE-----QLGLTR----A

FLJcow-N ---QKAVKIIAYKNGDGYRNGKLIVAG------TFPMLLTECTE-----QLGLAR----A

FLJdog-NC ---QKAVKIIAYKNGDGYRNGKLIVAG------TFPTLLTECTE-----QLGLTR----A

FLJrat-NC ---QKAVKIIAYKNGNGYRNGKLIVAG------TFHGLLAECTE-----RLQLTR----S

plaEAA2096 ------KSIWLYRNGDKHHNGLLFFIKS--HINNLKLLLFEITK-----VLNPIIG---P

DCLK3pa-NC ----KPRVVTVVKLGGQRPRKITLLLNR-RSVQTFEQLLADISE-----ALGSPRWKNDR

.

DCDC2_MO-N VRNIYTPRTGHRIRKLDQIESGGNYVA-GGPEAFKKLNY----

DCDC2cow-N VRNIYTPRT----------------------------------

DCDC2dog-N VRNIYTPRTGHRIRKLDQLQSGGNYVA-GGQEAFKKLNY----

DCDC2_HU-N VRNIYTPRTGHRIRKLDQIQSGGNYVA-GGQEAFKKLNY----

DCDC2pan-N VRNIYTPRTGHRIRKLDQIQSGGNYVA-GGQEAFKKLNY----

DCDC2opp-N VRNIYTPRAGHRIRKLDQIQSGGNYVA-GGLEAFKKL------

DCDC2rat-N VRNIYTPRTGHRIRKLDQIESGGNYVA-GGQEAFKKLY-----

DCDC2fro-N VRNIYTPRNGHRVTSLDDLQPGEFYVA-GGRENFKKLDY----

DCDC2fis-N VRTIYTPRGGHK-------------------------------

H.roretz-N VRNIYTPNAGHRVNELHEINNNMVIVA-GGAERFRKLHYQ---

cio-Sca_55_N VRNIYTPNLGHRVRDLTQLQNGMDLVA-GGVERFRKIQY----

DCDC2B_HU-N VRALYTPCHGHPVTNLADLKNRGQYVA-AGFERFHKL------

DCDC2Bpa-N VRALYTPCHGHPVTNLADLKNRGQYVA-AGFERFHKLHY----

DCDC2Bco-N VRALYTP-YGHPVTDLADLQNGGLYVA-AGFEHFHKLPY----

DCDC2Bdog VRALYTPCHGHRVTDLADLQNGGQYVA-AGFERFCKL------

DCDC2Bdo-N VRALYTPCHGHRVTDLADLQNGGQYVA-AGFERFCKLHV----

DCDC2B_MOU VRVLYTLSDGHPVTNLADLQNGGQYVA-AGFERFHKIHV----

DCDC2Bze-N VRTLYTPRYGHRVADLGDLQQGAQYVA-AGSERFKKLDY----

tetCAG1045 IRTLYTPRQGHRVPDLQHLQTGAQYVA-AGFEKFKKMDYL---

DCDC2Ach-N VRNLYTPKHGHRVAELADLQDGCQYVA-AGFEKFKRLDP----

DCDC2A_MO VRRLYTPTRGHPVLGLDALQTGGKYVA-AGRERFKKLE-----

DCDC2Arat VRRLYTPTYGHRVLELESLQTGGKYVA-AGRERFKKLE-----

DCDC2Ac-NC VRRLFTPTRGRPVLELDSLQAGGKYVA-AGRERFKKLE-----

DCDC2A_HUM VRRLFTPTRGHRVLGLDALQAGGKYVA-AGRERFKELDY----

DCDC2Apan VRRLFTPTRGHRVLGLDALQAGGKYVA-AGRERFKELE-----

DCDC2Adog VRRLFTPTRGHRVLELQALQAGGKYVA-AGREPFKKLDI----

DCDC2Aopp VRRLYTPCQGHRILELEQLQQGGKYVA-AGRERF---------

RP1_MOU-N VRNISTPRGRHSITRLEELEDGKSYVC-SHNKKVLPVDL----

RP1rat-N VRNISTPRGRHSITKLEELEDGESYVC-SHNKKVLPVDLD---

RP1_HUM-N VRNISTPRGRHSITRLEELEDGESYLC-SHGRKVQPVDL----

RP1pan-N VRNISTPRGRHSITRLEELEDGESYLC-SHGRKVQPVDL----

RP1_BOVIN-N VRNISTPRGRHSITRLEELEDGQSYLC-SHGRKVQPVDL----

RP1dog-N VRNISTPRGRHSITRLEELEDGASYLC-SHRRKVQPVDLD---

RP1frog-N VRNISTPRGIHHVTSINELEDGKSYIC-SHRKKIKPIN-----

RP1opp-NC VRNISTPRGIHGITKLEDLEDGRSYIC-SHKKKIKPID-----

RP1chick-N VRNISTPKGRHSITCLDDLEDGKSYIC-SHQRKMKPIN-----

RP1zebfi-N VRTITTPRGTHAVCSLDDVQDGGSYLC-SDQKKVKPFNLDE--

RP1fish-N VRNITTPHGVHAVHTLDELEDGKSYIC-SDMRKVKPINL----

RP1L1_MO-N VRSVTTPRGLHGLSALEQLQDGGCYLC-S--------------

RP1L1cow-N VRSVTTPRGLHGLSTLEQLQDGGCYLC-SDKKPPRTPGGLG--

RP1L1_HU-N VRSVTTPRGLHSLSALEQLEDGGCYLC-SDKKPPK--------

RP1L1opp-N VRSVTTPRGLHSLSALEQLEDGGCYLC-S--------------

RP1L1dog VRSVTTPRGLHGLSALEQLEDGGYYLC-S--------------

RP1L1chi-N VRTITTPRGIHCISELDQLEDGGCYLC-SDKKYVKPIN-----

anoEAL4118 ARYIFSMDG-DRKYSLDELEDGSSYVV-SSFKVFKKSK-----

drNP_64872 ARYVFSMDG-DRKYHLDELEDGAFYVV-SSFKAFKLVCRLCV-

A.mellif ARHIFSMDG-DRKLTLDELEDGASSTI-ESASSSS--------

DCX_MOU-N VRYIYTIDGSRKIGSMDELEEGESYVC-SSDNFFKKVEY----

DCX_HUM-N VRYIYTIDGSRKIGSMDELEEGESYVC-SSDNFFKKVEY----

DCXchick-N VRYIYTIDGSRKIGSMDELEEGESYVC-SSDNFFKKVEY----

DCXdog-N VRYIYTIDGSRKIGSMDELEEGESYVC-SSDNFFKKVEY----

DCXcow-N VRYIYTIDGSRKIGSMDELEEGESYVC-SSDNFFKKVEY----

DCXrat-N VRYIYTIDGSRKIGSMDELEEGESYVC-SSDNFFKKVEY----

DCXpan-N VRYIYTIDGSRKIGSMDELEEGESYVC-SSDNFFKKVEY----

DCXopp-N VRYIYTIDGGRKIGSMDELEEGDSYVC-SSDTFFKKVEY----

DCXfish-N VRFIFTIDGMNKITSLDELEEGESYVC-ASENFYKKVDY----

DCLKrat-N VRTIYTIDGLKKISSLDQLVEGESYVC-GSIEPFKKLEY----

DCLKpan-N VRTIYTIDGLKKISSLDQLVEGESYVC-GSIEPFKKLEY----

DCLKadog-N VRTIYTIDGLKKISSLDQLVEGESYVC-GSIEPFKKLEY----

DCLKdog-N VRTIYTIDGLKKISSLDQLVEGESYVC-GSIEPFKKLEY----

DCLK_HUM-N VRTIYTIDGLKKISSLDQLVEGESYVC-GSIEPFKKLEY----

DCLK_MOU-N VRTIYTIDGLKKISSLDQLVEGESYVC-GSIEPFKKLEY----

DCLKcow-N VRTIYTIDGLKKISSLDQLLEGESYVC-GSIEPFKKLEY----

DCLKopp-N VRTIYTIDGLKKISTLDQLVEGESYVC-GSIEPFKKLEY----

DCLKchi-N VRTIYTIDGSKKISSLDQLVEGESYVC-GSIEPFKKLEY----

DCLKfish-N VRTIYSVDGQTKITSIEQLVEGESYVC-ASIEPYKKVDY----

DCLKafish-N VRTIYTLDGTKKIGAIDQLVEGDSYVC-SSNEAYKKLDY----

DCLKzebfi-N VRIIYSIDGNKKITNIDQLVEGESYVC-GSTEAFKRVDY----

DCLK2_HU-N VRTIYTIDGSRKVTSLDELLEGESYVC-ASNEPFRKVDY----

DCLK2pan-N VRTIYTIDGSRKVTSLDELLEGESYVC-ASNEPFRKVDY----

DCLK2rat-N VRTIYTVDGSRKVTSLDELLEGESYVC-ASNEPFRKVDY----

DCLK2_MO-N VRTIYTIDGSRKVTSLDELLEGESYVC-ASNEPFRKVDY----

DCLK2dog-N VRTIYTIDGSRKVTSLDELLEGESYVC-ASNEPFRKVDY----

DCLK2cow-N VRTIYTIDGSRKVTSLDELLEGESYVC-ASNEPFRKVDY----

DCLK2chi-N VRTIYTIDGSKKLSSLDELLEGESYVC-ASNEPYRKVDY----

DCLK2opp-N VRTIYTIDGSRKLTSLEELMEGKSEGK-ESD--FIK-------

DCLK2zeb-N VRSIYTADGGKKITSLDDLVEGESYVC-ASNEPFRKVDY----

DCLK2azebf VRNIYTADGAKKISSLEELAEGESYVC-ASNEPYRKVDY----

DCLK2fis-N VRTIYSVDGSKKIGSMDELVEGESYVC-ASNEPYKKLEY----

cio-Sca_14_N VRVVFSLDGARKIETIQELEHGESYVC-ASSDMFKRLEY----

anoXP_3197 IRAIYTLEG-KKIEKVDDLEDGKCYVCSCNNEGFKRIDY----

D.melano-N VRTIYNLCG-KKITSLDELEDGQSYVCSCNNENFKKVEY----

A.mellif-N VRAIYTMDG-KKVQSINDLEDGKCYVVSGQGEIFKKVEY----

drNP_65115 IAHFRRTDG-SHLTSLSCFRETDIVICCCKNEEIICVKY----

ZYG-8-N IRHIFTIDGAQRITSVDQFEDGGGYVC-SSTDAFKPVDY----

cbZYG-8-N IRHIFTMDGTQRITSVDQFEDGGGYVC-SSTDVFKPVDY----

cio-Sca_10_N VRRIYTPIGGTLIEDIEELKDGESYVC-ASFEKFQRIKY----

DdDCX-N VRKLYLASSGKLVKTMEEIIDGEYYVC-AGGETLNPLDFS---

ceNP_00102 AKKLYTTDG-KIVNSIKELEDGKIYVA--ASAQF---------

ceCAB04937 AKKLYTTDG-KIVNSIKELEDGKIYVA--ASAQF---------

cbCAE73316 AKKLYTTDG-KVVGSIKELEDGKIYVA--ASAQF---------

cbCAE73319 AKKLYTTSG-TLIKDINKIKDGENYVA--SSSHF---------

mBAC26042 AKTLYTSNG-ELIQSWDDIERGMAVCVSAGHGF----------

BAC26042ra AKTLYTSNG-ELIQSWDEIEKGMAVCVSAGHGF----------

DCDC1dog ARYLYDLYG-RKIKDISKGK-----------------------

urcXP_7883 ARYIYTWDG-QKLEDLNELPR-LDGCLQSS-------------

DCDC1_HUMAN ARRVFLADG-KEALEPEDIPHEADVYVSTG-------------

DCDC1pan ARRVFLADG-KEALEPEVIPHEADVYVSTGEPFLNPFKKIK--

bovXP_8740 ARRVFLADG-TEALEPEDIPREADVYVSTGESFLDPFKKIK--

hFLJ-N ASKVYTKDG-T--------------------------------

FLJpan-NC ASKVYTKDG-TCTEILNLPSAARRLYNEKGKEIFALKDLQRDE

FLJcow-N ASKIYTKDG-T--------------------------------

FLJdog-NC ASKVYTKDG-TT--------------------VLSLRD-----

FLJrat-NC ASKIYTRDG-T--------------------------------

plaEAA2096 IRKIYDQNF-RLIKNIQQLNDGSKYLC-TS-------------

DCLK3pa-NC VRKLFNLKGREIRSVSDFFREGDAFI-----------------

.

Proteins with C-terminal domains

CLUSTAL W (1.83) multiple sequence alignment

DCDC2_MO-C -------------------PCTIFLIANGDL-ISPASRLLIPKKALNQWDHVLQMVTEKI

DCDC2rat-C ------------------------LIANGDL-ISPASRLLIPRKALNQWDHVLQMVTEKI

DCDC2cow-C ---------------PLLEPCTIFLIANGDL-INPASRLLIPRKALNQWDHVLQMITEKI

DCDC2_HU-C ---------------PLQEPCTIFLIANGDL-INPASRLLIPRKTLNQWDHVLQMVTEKI

DCDC2pan-C -------------------PCTIFLIANGDL-INPASRLLIPRKTLNQWDHVLQMVTEKI

DCDC2dog-C ---------------PLQEPCTIFLIANGDL-ISPASRLLIPRKTLNQWDHVLQMVTEKI

DCDC2opp-C ------------------------LIANGDL-ISPASRLFIPRKALNQWDLVLEMVTEKI

DCDC2chick -------------------PYTIFLIANGDL-ISPVVRLLIPRKTLNHWDHILEMVTAKV

DCDC2fro-C -------------------PCTVFLVANGDT-LNPFIRLLIPRKTLEQWELVLALVTEKV

DCDC2fis-C -------------------PCAVFVVANGDV-LNSAVRLLIHQRMLGQFDKILEMITEKM

DCDC2B_HUM ----------------FERFHKLHVFRNGDL-VSPPFSLKLSQAASQDWETVLKLLTEKV

DCDC2Bpa-C -------------------PTYIHVFRNGDL-VSPPFSLKLSQAASQDWETVLKLLTEKV

DCDC2Bdo-C ------------------------VFRNGDL-LSPPFSLKLSQAASEDWETVLKLLTEKA

DCDC2Bchic ----------------------IHVFRNGDL-LSPPFQLMISKSTLWQWDTLLATLTEKA

DCDC2Bze-C -------------------PCIIHVFRNGDI-LSPAMRLIIPRHMLKNLEQILSLISEKA

H.roretz-C -----------------------------------AIKMLLSARIMKNWDMVLEEITEKI

cio-Sca_55_C -----------------KEPCQIYVYGNGDI-NAPAIRLLLIPRAMKSWDLVLSEITEKI

RP1_MOU-C ---------------MLRAPRRLVVFRNGDP--KNKHVVLLSRRITQSFEAFLQYLTQVM

RP1rat ----------------FRAPRRLVVFRNGDP--KTRRVVLLSRRITQSFEAFLQYLTQVM

RP1_HUM-C -------------------PRSLVVFRNGDP--KTRRAVLLSRRVTQSFEAFLQHLTEVM

RP1pan-C -------------------PRSLVVFRNGDP--KTRRAVLLSRRVTQSFEAFLQHLTEVM

RP1_BOV-C --------------GMLRAPRRLVVFRNGDP--KTRRAIVLNRRVTQSFEVFLQYLTQVM

RP1dog-C ---------------MLRAPRRLLVFRNGDP--KIRRVVIVNRRVTQSFQAFLQHLTEVM

RP1chick-C ---------------KITTPKKMLVFKNGDV--RLRRTIVLGKKNTQTFEAFLDYMSELM

tetCAG1227 -------------------PKKLVVISNRDP--TFKRTIVLHRRSAPTFDALLDYLSQIL

RP1zebfish-C -------------------PKRLTVYKNRDP--SMKRVIVLHRRIAPTFEALLDYLSQMM

RP1fish-C ---------------VLRTPKRLVVFCNGVP--AVHHTLVLDKRITPTFETILEYISEVV

RP1frog-C ----------------------------------------LNKKTKQSFDSFLDQVAEAL

RP1L1_HU-C -------------------PRRILLIKNMDP--RLQQTVVLSHRNTRNLAAFLGKASDLL

RP1L1pan-C ---------------------------------------VLSHRNTRNLAAFLSKASDLL

RP1L1cow-C ---------------GLKASRRITLVKNGDP--QLQQTVVLSHRNTRNLTAFLSKASDLL

RP1L1dog-C -------------------PRRIVLVKNGDP--RFQQTVVLSHRNTRNLMAFLSKASDLL

RP1L1_MO-C -----------------VAPRRLTLVKNGDP--RRQQTVVLSHKNTRSLAAFLGKASELL

RP1L1chi-C ---------------GPRIPKKITLVKNGET--SFRRSIILNRRNARSFKTLLDEISEIL

DCX_MOU-C ---------------DFVRPKLVTIIRSGVK-PRKAVRVLLNKKTAHSFEQVLTDITEAI

DCXpan-C ---------------DFVRPKLVTIIRSGVK-PRKAVRVLLNKKTAHSFEQVLTDITEAI

DCXopp-C ---------------DFVRPKLVTIIRSGVK-PRKAVRVLLNKKTAHSFEQVLTDITEAI

DCXrat-C ---------------DFVRPKLVTIIRSGVK-PRKAVRVLLNKKTAHSFEQVLTDITEAI

DCXchick-C ---------------DFVRPKLVTIIRSGVK-PRKAVRVLLNKKTAHSFEQVLTDITEAI

DCXcow-C ---------------DFVRPKLVTIIRSGVK-PRKAVRVLLNKKTAHSFEQVLTDITEAI

DCXdog-C ---------------DFVRPKLVTIIRSGVK-PRKAVRVLLNKKTAHSFEQVLTDITEAI

DCX_HUM-C ---------------DFVRPKLVTIIRSGVK-PRKAVRVLLNKKTAHSFEQVLTDITEAI

DCXfish-C ---------------DFVRPKLVTVMRSGVK-PRKAVRVLLNKKTAHSFEQVLTDITEAI

DCLK2chi-C ---------------DFIKPKLVTVIRSGVK-PRKAVRILLNKKTAHSFEQVLTDITEAI

DCLK2rat-C ---------------DFIKPKLVTVIRSGVK-PRKAVRILLNKKTAHSFEQVLTDITEAI

DCLK2_MO-C ---------------DFIKPKLVTVIRSGVK-PRKAVRILLNKKTAHSFEQVLTDITEAI

DCLK2dog-C ---------------DFIKPKLVTVIRSGVK-PRKAVRILLNKKTAHSFEQVLTDITEAI

DCLK2pan-C ---------------DFIKPKLVTVIRSGVK-PRKAVRILLNKKTAHSFEQVLTDITEAI

DCLK2opp-C ---------------DFIKPKLVTVIRSGVK-PRKAVRILLNKKTAHSFEQVLTDITEAI

DCLK2_HU-C ---------------DFIKPKLVTVIRSGVK-PRKAVRILLNKKTAHSFEQVLTDITEAI

DCLK2cow-C ---------------DFIKPKLVTVIRSGVK-PRKAVRILLNKKTAHSFEQVLTDITEAI

DCLK2fis-C ---------------DFIKPKLVTVIRSGVK-PRKAVRILLNKKTAHSFEQVLADITEAI

DCLKopp-C ---------------DFIRPKLVTIIRSGVK-PRKAVRILLNKKTAHSFEQVLTDITDAI

DCLKrat-C ---------------DFIRPKLVTIIRSGVK-PRKAVRILLNKKTAHSFEQVLTDITDAI

DCLKdog-C ---------------DFIRPKLVTIIRSGVK-PRKAVRILLNKKTAHSFEQVLTDITDAI

DCLK_MOU-C ---------------DFIRPKLVTIIRSGVK-PRKAVRILLNKKTAHSFEQVLTDITDAI

DCLKadog-C ---------------DFIRPKLVTIIRSGVK-PRKAVRILLNKKTAHSFEQVLTDITDAI

DCLK_HUM-C ---------------DFIRPKLVTIIRSGVK-PRKAVRILLNKKTAHSFEQVLTDITDAI

DCLKchi-C ---------------DFIRPKLVTIIRSGVK-PRKAVRILLNKKTAHSFEQVLTDITDAI

DCLK2zeb-C ---------------DYIKPKLVTVIRSGVK-PRKAVRILLNKKTAHSFEQVLTDITDAI

DCLKzebfi-C ---------------DFIRPKLVTVIRSGVK-PRKAVRILLNKKTAHSFEQVLTDITDAI

DCLKpan-C ---------------DFIRPKLVTIIRSGVK-PRKAVRILLNKKTAHSFEQVLTDITDAI

DCLKcow-C ---------------DFIRPKLVTIIRSGVK-PRKAVRILLNKKTAHSFEQVLTDITDAI

DCLKfish-C ---------------DFIKPKLVTIIRSGVK-PRKAVRVLLNKKTAHSFDQVLTDITDAI

DCLKafish-C ---------------DFIRPKLVTVVRSGVK-PRKAVRILLNKKTAHSYEQVLTDITDAI

cio-Sca_14_C -----------------------TIIRSGVK-PRKAVRILLNKKTAHSLEQVLNDVTKAI

A.gambia-C ---------------SVVHPRIVTLIRNGVK-PRKILRLLLNKRNSPTYEHVLTAITQCV

D.melano-C -----------------VHPRIVTLIRSGTK-SRRIMRLLLNKRNSPSFDHVLTAITQVV

A.mellif-C ---------------LCVKAKIITLIRHGTK-PRKVVRLLLNKRNAPSLEHALEAITEAV

ZYG-8-C ---------------DFVFPRIIKVIRNGVK-PRRISRHLLNKKTARSFDQVLRDLTFVV

cbZYG-8-C ---------------DFVIPKIIKVIRNGVK-PRRISRHLLNKKTARSFDQVLRDLTLIV

DCLK3_MOU --------------HSPLKPRVVTVVKLGGQ-PLRKATLLLNRRSVQTFEQLLSDISEAL

DCLK3rat -----------------LKPRVVTVVKLGVQ-PLRKATLLLNRRSVQTFEQLLSDISEAL

DCLK3_HUM -----------------LKPRVVTVVKLGGQ-RPRKITLLLNRRSVQTFEQLLADISEAL

DCLK3pa-NC ---------------------VVTVVKLGGQ-RPRKITLLLNRRSVQTFEQLLADISEAL

BAB21856co ---------------SLLKPRVVTVVKVGSH-PLRKITLLLNRRSVQTFEQLLADVSEAL

DCLK3opp ----------------------------------RKITLLLNRRSVLTFEQLVADISEAL

cio-Sca_10_C -----------------MKPKVVTVVRAGQIRPHKKITILLNRRAVQTYEQLVSDISEAL

A.mellifer -------------------------------------RVLLNLRTSQPFEEVLEDLGQVL

anoEAA0645 ------------GSLKPSAGRVIRIINSHDH--SVQCRVLLNLRTSQPFEEVLEDLGQVL

EMAL_DROME --------------------------------------------------EVLEDLGQVL

DCDC2A_MOU ---------------DTTPAKTILVYRNGDQ-FYVGRKFVFSRRRVANFEALLEQLTEQV

DCDC2Arat --------------------KTILVYRNGDQ-FYVGRKFVFSRRRVANFEALLEQLTEQV

DCDC2Ac-NC --------------------KTIVVYRNGDQ-FYVGRKFVLSRRRVATFEALLEQLTEQV

DCDC2A_HUM ---------------DTTPAKTIVVYRNGDP-FYVGKKFVLSRRRAATFEALLEQLTEQV

DCDC2Apan --------------------KTIVVYRNGDP-FYVGKKFVLSRSRAATFEALLEQLTEQV

DCDC2Aopp ---------------DITPARTILVYRNGDE-FYVGTKFVINRKRVPNIEALMTQLNDKL

DCDC2B_MOU --------------------KRILVYRNGDA-FFPGHQLVVTQRRYPTMEALLYEVTSAV

DCDC2Bco-C ---------------GSPAAKRVVVYQNGDP-FSPGRQLVVTQRRFPTLETFLCEVTSAV

DCDC2Ach-C --------------ALAHPAKNVVVYRNGDP-FFHGRKFVVNQRQFLTFEAFLNEVTKSI

RP1opp-NC --------------------KRISFYKSGDP-QFNGIQMVVNPRSFKSFDALLDNLSKKV

FLJdog-NC -----------------QKAVKIIAYKNGDG-YRNG-KLIVAG----TFPTLLTECTEQL

FLJpan-NC -----------------QKAVKIIAYKNGDG-YRNG-KLIVAG----TFPMLLTECTEQL

FLJrat-NC -----------------QKAVKIIAYKNGNG-YRNG-KLIVAG----TFHGLLAECTERL

ceNP_00102 ----------------------ISIYKNGDR-YHRGVKFVINPRVIKDMEPLLNQVNDRI

mFLJ-C ---EGLLDTNSSPMKRMASKRPDLLVPMRLR-VLRNGEKKNIRPLRQEPRVKRTQCTDIL

hFLJ-C -----------------------------------------------------TQCTEIL

FLJcow-C ------------------------------------------------FHQLLERCTEIL

urcXP_7883 RSAASSVSTYSQSSKGKFQNRLQPHVIRTMC-YRNGSREKSVKITAPTMKIFLEYCTLKL

DdDCX-C ----------------------------------EGVHCLVHSSKFKTFDQLKLEFSKKV

drNP_99582 -------------------GNMRLLITNLLK--DCLLDHKIVRVLVRCMEQLITDTNDRI

DCDC2_MO-C T--LRS-GAVHRLYTL-EGKL---------------------------------------

DCDC2rat-C T--LRS-GAVHRLYTL-EGKL---------------------------------------

DCDC2cow-C T--LRS-GAVHRLYTL-EGKL---------------------------------------

DCDC2_HU-C T--LRS-GAVHRLYTL-EGKL---------------------------------------

DCDC2pan-C T--LRS-GAVHRLYTL-EGKL---------------------------------------

DCDC2dog-C T--LRT-GAVHRLYTL-EGKP---------------------------------------

DCDC2opp-C T--LRS-GAVHRLYTL-EGKL---------------------------------------

DCDC2chick S--LRS-GAVHRLYTL-DGKH---------------------------------------

DCDC2fro-C K--LRN-GAVHRLYTL-EGTP---------------------------------------

DCDC2fis-C G--LRVLGGVRSLYTY-DGTQ---------------------------------------

DCDC2B_HUM K--LQS-GAVCKLCTL-EGLP---------------------------------------

DCDC2Bpa-C K--LQS-GAVCKLCTL-EGLP---------------------------------------

DCDC2Bdo-C K--LQA-GAVCKLCTL-EGLP---------------------------------------

DCDC2Bchic D--LCS-GAVNRLCKL-DGTL---------------------------------------

DCDC2Bze-C M--LRT-GAVRRICTL-EGFT---------------------------------------

H.roretz-C S--IRTGQAVRRLYTL-DGVL---------------------------------------

cio-Sca_55_C C--LRTGKAVRKLYDM-DYHL---------------------------------------

RP1_MOU-C ------QCPVAKLYAT-DGRK---------------------------------------

RP1rat ------QYPVAKLYAT-DGRK---------------------------------------

RP1_HUM-C ------QRPVVKLYAT-DGRR---------------------------------------

RP1pan-C ------QRPVVKLYAT-DGRR---------------------------------------

RP1_BOV-C ------QRPVTKLYAT-DGRK---------------------------------------

RP1dog-C ------RFPVTKLYAT-DGRK---------------------------------------

RP1chick-C ------QYPVAKLYTT-DGRK---------------------------------------

tetCAG1227 ------QFPVLKLYST-DGRR---------------------------------------

RP1zebfish-C ------QFPVVKLYTE-DGRR---------------------------------------

RP1fish-C ------QFHVVKLHML-DGRR---------------------------------------

RP1frog-C ------QYPVFKLYSS-DGRR---------------------------------------

RP1L1_HU-C ------RFPVKQLYTT-SGKK---------------------------------------

RP1L1pan-C ------RFPVKQLYTT-SGKK---------------------------------------

RP1L1cow-C ------RFPVKHVYTT-GGKR---------------------------------------

RP1L1dog-C ------HFPVKQVYTT-SGKK---------------------------------------

RP1L1_MO-C ------RFPVKQVYTT-RGKK---------------------------------------

RP1L1chi-C ------QFPVKKLFTV-DGKK---------------------------------------

DCX_MOU-C ---KLETGVVKKLYTL-DGKQ---------------------------------------

DCXpan-C ---KLETGVVKKLYTL-DGKQ---------------------------------------

DCXopp-C ---KLETGVVKKLYTL-DGKQ---------------------------------------

DCXrat-C ---KLETGVVKKLYTL-DGKQ---------------------------------------

DCXchick-C ---KLETGVVKKLYTL-DGKQ---------------------------------------

DCXcow-C ---KLETGVVKKLYTL-DGKQ---------------------------------------

DCXdog-C ---KLETGVVKKLYTL-DGKQ---------------------------------------

DCX_HUM-C ---KLETGVVKKLYTL-DGKQ---------------------------------------

DCXfish-C ---KLESGVVKRIYTL-DGKQ---------------------------------------

DCLK2chi-C ---KLDSGVVKRLCTL-DRKQ---------------------------------------

DCLK2rat-C ---KLDSGVVKRLCTL-DGKQ---------------------------------------

DCLK2_MO-C ---KLDSGVVKRLCTL-DGKQ---------------------------------------

DCLK2dog-C ---KLDSGVVKRLCTL-DGKQ---------------------------------------

DCLK2pan-C ---KLDSGVVKRLCTL-DGKQVRSHFGVSSCFRKVEVALPLGIGEALSLLS---------

DCLK2opp-C ---KLDSGVVKRLCTL-DGKQ---------------------------------------

DCLK2_HU-C ---KLDSGVVKRLCTL-DGKQ---------------------------------------

DCLK2cow-C ---KLDSGVVKRLCTL-DGKQ---------------------------------------

DCLK2fis-C ---KLDSGAVKRLYTL-DGKQ---------------------------------------

DCLKopp-C ---KLDSGVVKRLYTL-DGKQ---------------------------------------

DCLKrat-C ---KLDSGVVKRLYTL-DGKQ---------------------------------------

DCLKdog-C ---KLDSGVVKRLYTL-DGKQ---------------------------------------

DCLK_MOU-C ---KLDSGVVKRLYTL-DGKQ---------------------------------------

DCLKadog-C ---KLDSGVVKRLYTL-DGKQ---------------------------------------

DCLK_HUM-C ---KLDSGVVKRLYTL-DGKQ---------------------------------------

DCLKchi-C ---KLDSGVVKRLYTL-DGKQIFLAFRLIAAVDARAFWHTSGKHDMNFIAPPLPVAAGWE

DCLK2zeb-C ---KLDSGAVKRLYTL-EGKQ---------------------------------------

DCLKzebfi-C ---KLDSGIVKRIYTL-EGKQ---------------------------------------

DCLKpan-C ---KLDSGVVKRLYTL-DGKQ---------------------------------------

DCLKcow-C ---KLDSGVVKRLYTL-DGKQ---------------------------------------

DCLKfish-C ---KLDSGVVRRLYTV-DGKM---------------------------------------

DCLKafish-C ---KLDSGVVKKIYTL-EGKL---------------------------------------

cio-Sca_14_C ---KLDTGAVRKVYTL-QGKQ---------------------------------------

A.gambia-C ---KLDTGCVRKVFTV-AGVP---------------------------------------

D.melano-C ---RLDTGYVRKVFTL-SGIP---------------------------------------

A.mellif-C ---KLDSGAVRKVYTL-SGQQ---------------------------------------

ZYG-8-C ---KLDSGAIRKLFTL-SGRP---------------------------------------

cbZYG-8-C ---KLDAGAIRKLFTL-SGRP---------------------------------------

DCLK3_MOU GFPRWKNDRVRKLFTL-KGRE---------------------------------------

DCLK3rat GFPRWKNDRVRKLFTL-KGRE---------------------------------------

DCLK3_HUM GSPRWKNDRVRKLFNL-KGRE---------------------------------------

DCLK3pa-NC GSPRWKNDRVRKLFNL-KGRE---------------------------------------

BAB21856co GFPRWKSDRVRKLFNL-KGRE---------------------------------------

DCLK3opp GFPRWKNDRVRKLYSL-KGKE---------------------------------------

cio-Sca_10_C GQPKWKNDHIRRLYTL-KGRE---------------------------------------

A.mellifer -----KMNGAKRMFTV-SGQE---------------------------------------

anoEAA0645 -----KMIGAKKMYTS-NGQE---------------------------------------

EMAL_DROME -----KINGAKKMYTG-TGQE---------------------------------------

DCDC2A_MOU E----VPFGVRRLYTPTRGHP---------------------------------------

DCDC2Arat E----VPFGVRRLYTPTYGHR---------------------------------------

DCDC2Ac-NC E----VPFGVRRLFTPTRGRP---------------------------------------

DCDC2A_HUM D----VPFGVRRLFTPTRGHR---------------------------------------

DCDC2Apan D----VPFGVRRLFTPTRGHR---------------------------------------

DCDC2Aopp A----VPFGVRRLYTPCQGHR---------------------------------------

DCDC2B_MOU Q----APLAVRVLYTLSDGHP---------------------------------------

DCDC2Bco-C R----APVAVRALYTP-YGHP---------------------------------------

DCDC2Ach-C H----APLAVRNLYTPKHGHR---------------------------------------

RP1opp-NC P----LPFGVRNISTPRGIHG---------------------------------------

FLJdog-NC G----LTRAASKVYTK-DGTT---------------------------------------

FLJpan-NC G----LTRAASKVYTK-DG-----------------------------------------

FLJrat-NC Q----LTRSASKIYTR-DG-----------------------------------------

ceNP_00102 E----LSHGAKKLYTT-DGKI---------------------------------------

mFLJ-C N----LPSAARRLFSE-KGKE---------------------------------------

hFLJ-C N----LPSAARRLYNE-KGKE---------------------------------------

FLJcow-C N----LPSAARRLFNE-KGKE---------------------------------------

urcXP_7883 D----FEFAARRIFLE-DGTE---------------------------------------

DdDCX-C G---LFTGNVQKVYSM-DKKR---------------------------------------

drNP_99582 Q---YFIEIIYELCELNTKQND-------------------------------------L

:

DCDC2_MO-C ---VESGAELE---NGQFYVAVGRDKFKRLPYSE-----

DCDC2rat-C ---VESGAELE---NGQFYVAVGRDKFKRLPYSE-----

DCDC2cow-C ---VESGAELE---NGQFYVAVGRDRFKKLPYSE-----

DCDC2_HU-C ---VESGAELE---NGQFYVAVGRDKFK-----------

DCDC2pan-C ---VESGAELE---NGQFYVAVGRDKFK-----------

DCDC2dog-C ---VESGAELE---NGQFYVAVGRDKFKKLPY-------

DCDC2opp-C ---VQSGSELE---NGQFYVAVGRDKFK-----------

DCDC2chick ---VQNGSDLE---KGQFYVAVGREKFKK----------

DCDC2fro-C ---IQNGLELE---NGQFYVAVGREKFKK----------

DCDC2fis-C ---VNDGNQLE---SGQLYVAVGRERFKK----------

DCDC2B_HUM ---LSAGKELV---TGHYYVAVGEDEFK-----------

DCDC2Bpa-C ---LSAGEELV---TGHYYVAVGEDEFK-----------

DCDC2Bdo-C ---LSAREALV---NGHYYVAVGEEEFK-----------

DCDC2Bchic ---VSSREELV---NGNYYVAVGTEEYKK----------

DCDC2Bze-C ---VTSAEELE---TGQCYVAVGSERFKKLPY-------

H.roretz-C ---IQGSENLE---NGRYYVAVGYERFKRA---------

cio-Sca_55_C ---LGDPSELE---NGKYYIAVGTERIKKIAY-------

RP1_MOU-C ---VPSLQAVIL--SSGAVVAAGREPFKPGNYD------

RP1rat ---VPSLQAVIL--SSGAVVAAGREPFKP----------

RP1_HUM-C ---VPSLQAVIL--SSGAVVAAGREPFK-----------

RP1pan-C ---VPSLQAVIL--SSGAVVAAGREPFK-----------

RP1_BOV-C ---VPSLQAVIL--SSGAVVAAGREPFK-----------

RP1dog-C ---VPSLQAVIL--SSGAVVAAGREPFK-----------

RP1chick-C ---VPNLQALIL--CSGAIVAAGREPFKPSNYE------

tetCAG1227 ---IDGLAALIL--CSGVVVAAGNEPFK-----------

RP1zebfish-C ---IEGLSALIL--CTGIVVAAGNEPFR-----------

RP1fish-C ---VDGLPGLIL--CSGVVVAAGREPFRAANY-------

RP1frog-C ---ILSIRALLL--SSGTVVAAGRESFIYA---------

RP1L1_HU-C ---VDSLQALLH--SPSVLVCAGHEAFR-----------

RP1L1pan-C ---VDSLQALLH--SPSVLVCAGHEAFR-----------

RP1L1cow-C ---VDSLKALLR--SPSVLVCAGLEPFRPLVTED-----

RP1L1dog-C ---VDSLKGLLH--SPSVLVCAGYESFK-----------

RP1L1_MO-C ---VDSLQTLLD--GPSVLVCAGNEAFR-----------

RP1L1chi-C ---IDSMQALLH--CPNVLVCVGREPFKPVSME------

DCX_MOU-C ---VTCLHDFFG--DDDVFIACGPEKFRYAQDD------

DCXpan-C ---VTCLHDFFG--DDDVFIACGPEKFRYAQDD------

DCXopp-C ---VTCLHDFFG--DDDVFIACGPEKFRYAQDD------

DCXrat-C ---VTCLHDFFG--DDDVFIACGPEKFRYAQDD------

DCXchick-C ---VTCLHDFFG--DDDVFIACGPEKFRYAQDD------

DCXcow-C ---VTCLHDFFG--DDDVFIACGPEKFRYAQDD------

DCXdog-C ---VTCLHDFFG--DDDVFIACGPEKFRYAQDD------

DCX_HUM-C ---VTCLHDFFG--DDDVFIACGPEKFRYAQDD------

DCXfish-C ---VTCLQDFFG--DDDVFIACGPEKFRYAQDD------

DCLK2chi-C ---VTCLQDFFG--DDDVFIACGPEKYRYAQDD------

DCLK2rat-C ---VTCLQDFFG--DDDVFIACGPEKYRYAQDD------

DCLK2_MO-C ---VTCLQDFFG--DDDVFIACGPEKYRYAQDD------

DCLK2dog-C ---VTCLQDFFG--DDDVFIACGPEKFRYAQDD------

DCLK2pan-C ---VTCLQDFFG--DDDVFIACGPEKFRYAQDD------

DCLK2opp-C ---VTCLQDFFG--DDDVFIACGPEKFRYAQDD------

DCLK2_HU-C ---VTCLQDFFG--DDDVFIACGPEKFRYAQDD------

DCLK2cow-C ---VTCLQDFFG--DDDVFIACGPEKFRYAQDD------

DCLK2fis-C ---LTCLQDFFG--DDDVFMACGPEKFRYAQDD------

DCLKopp-C ---VMCLQDFFG--DDDIFIACGPEKFRYQDD-------

DCLKrat-C ---VMCLQDFFG--DDDIFIACGPEKFRYQD--------

DCLKdog-C ---VMCLQDFFG--DDDIFIACGPEKFRYQDD-------

DCLK_MOU-C ---VMCLQDFFG--DDDIFIACGPEKFRYQDD-------

DCLKadog-C ---VMCLQDFFG--DDDIFIACGPEKFRYQDD-------

DCLK_HUM-C ---VMCLQDFFG--DDDIFIACGPEKFRYQDD-------

DCLKchi-C VVMVMCLQDFFG--DDDIFIACGPEKFRYQDD-------

DCLK2zeb-C ---------------------------------------

DCLKzebfi-C ----------------TAFIKKEREELTI----------

DCLKpan-C ---------------------------------------

DCLKcow-C ---------------------------------------

DCLKfish-C ---VTCLQDFFA--EDDIFFACGPEKFRYQDD-------

DCLKafish-C ---VSCLQDFFG--DEDVFVACGPEKFRYQDD-------

cio-Sca_14_C ---VQSLQDFFG--NDDIFIAYGHEKLSQDD--------

A.gambia-C ---VQRLAQFFE--EDDVFFAYGNER-------------

D.melano-C ---VVRLSDFFG--SDDVFFAYGTERINTAED-------

A.mellif-C ---VTSLEQFFE--NDDIFVAYGPEK-------------

ZYG-8-C ---VLSLQDFFR--DDDVFVAYG----------------

cbZYG-8-C ---VLTLQDFFR--EDDVFVAYGGNDKMAADD-------

DCLK3_MOU ---VKSVSDFFR--EGDAFIAMGKE--------------

DCLK3rat ---VKSVSDFFR--EGDAFIAMGKE--------------

DCLK3_HUM ---IRSVSDFFR--EGDAFIAMGKEP-------------

DCLK3pa-NC ---IRSVSDFFR--EGDAFIAMGKE--------------

BAB21856co ---IRSVSDFFR--EGDAFIAVGKEPLTLKNIQVAIEEL

DCLK3opp ---VKSVSDFFR--EGDAFIAMGRE--------------

cio-Sca_10_C ---IRSVSDFFR--EDDVFIAVGREQ-------------

A.mellifer VRSFSQLRNEFA--DVDTF--------------------

anoEAA0645 VRSFSQLRNEFA--EVETFYLSNTPSLPVGALGP-----

EMAL_DROME VRSFSQLRNEFA--DVDTFYLATGTALIAGSPIRR----

DCDC2A_MOU ---VLGLDALQ---TGGKYVAAGRERFK-----------

DCDC2Arat ---VLELESLQ---TGGKYVAAGRERFK-----------

DCDC2Ac-NC ---VLELDSLQ---AGGKYVAAGRERFK-----------

DCDC2A_HUM ---VLGLDALQ---AGGKYVAAGRERFK-----------

DCDC2Apan ---VLGLDALQ---AGGKYVAAGRERFK-----------

DCDC2Aopp ---ILELEQLQ---QGGKYVAAGRERF------------

DCDC2B_MOU ---VTNLADLQ---NGGQYVAAGFERF------------

DCDC2Bco-C ---VTDLADLQ---NGGLYVAAGFEHFHKLPYVM-----

DCDC2Ach-C ---VAELADLQ---DGCQYVAAGFEKFKRLDPVP-----

RP1opp-NC ---ITKLEDLE---DGRSYICSH----------------

FLJdog-NC ---VLSLRDLVLWALDESFIQRNTEK-------------

FLJpan-NC ---------------------------------------

FLJrat-NC ---------------------------------------

ceNP_00102 ---VNSIKELE---DGKIYVAASAQ--------------

mFLJ-C ---LFSLKDLQ--RDELVYVSCG----------------

hFLJ-C ---IFALKDLQ--RDELVYVSCG----------------

FLJcow-C ---VFTLKELQ--RDELVYVSCG----------------

urcXP_7883 ---VKSAEEIP--RDGEVYISGGEPFK------------

DdDCX-C ---IQDIKDFV---DGHHYICCGAE--------------

drNP_99582 IHDRSLINKLLDDLDTPLKMKISSLKVKILELEELE---
