# Supplementary figures and images for: The evolving doublecortin (DCX) superfamily
Source: BMC Genomics. 2006 Jul 26;7:188. doi: 10.1186/1471-2164-7-188 (PMC1550402; doi:10.1186/1471-2164-7-188)

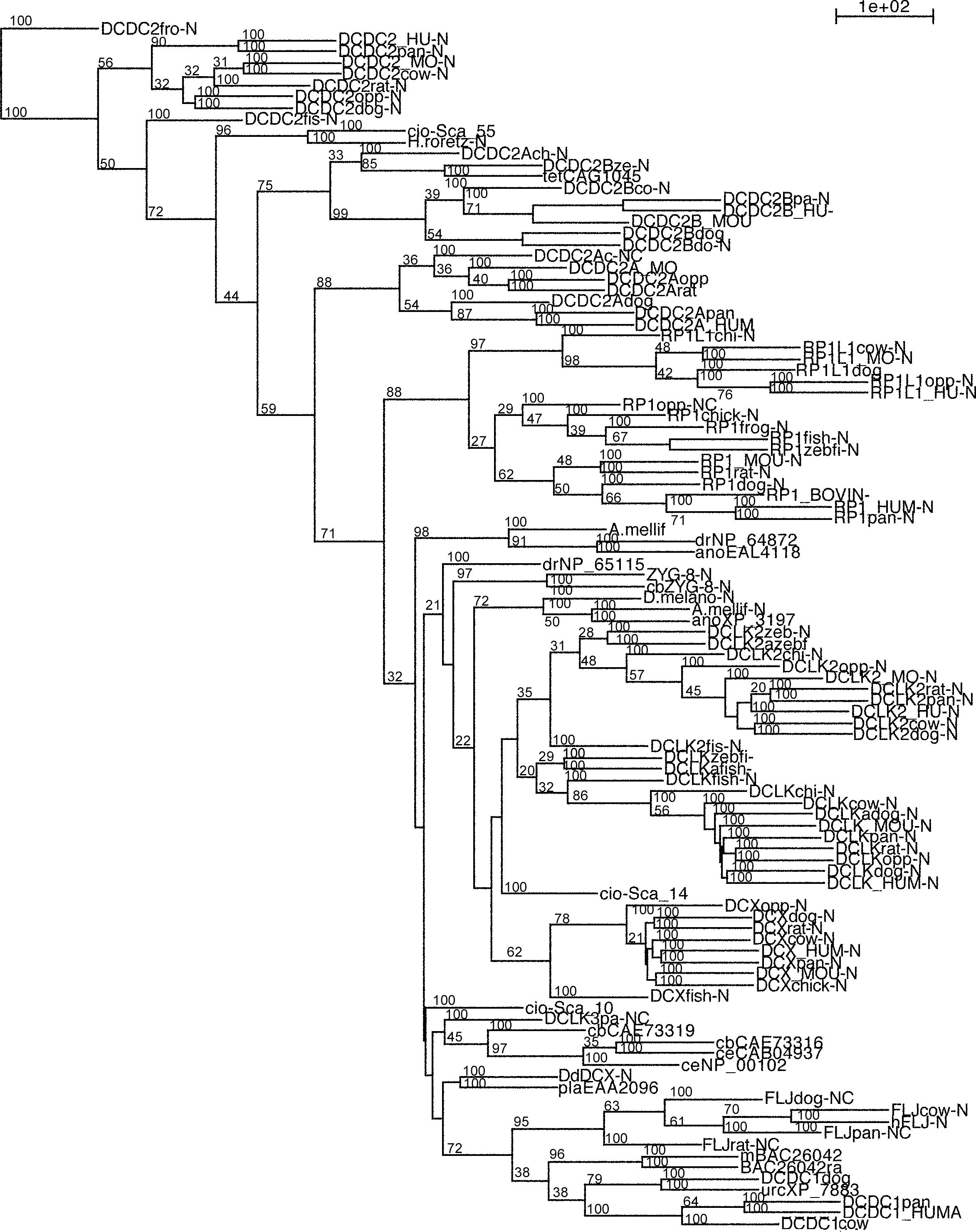

Supplement: Additional File 4 — Supplementary Fig. 4: ML tree of the N-terminal DCX domain proteins from different species. Bootstrap values are indicated. [file 1471-2164-7-188-S4.jpeg]

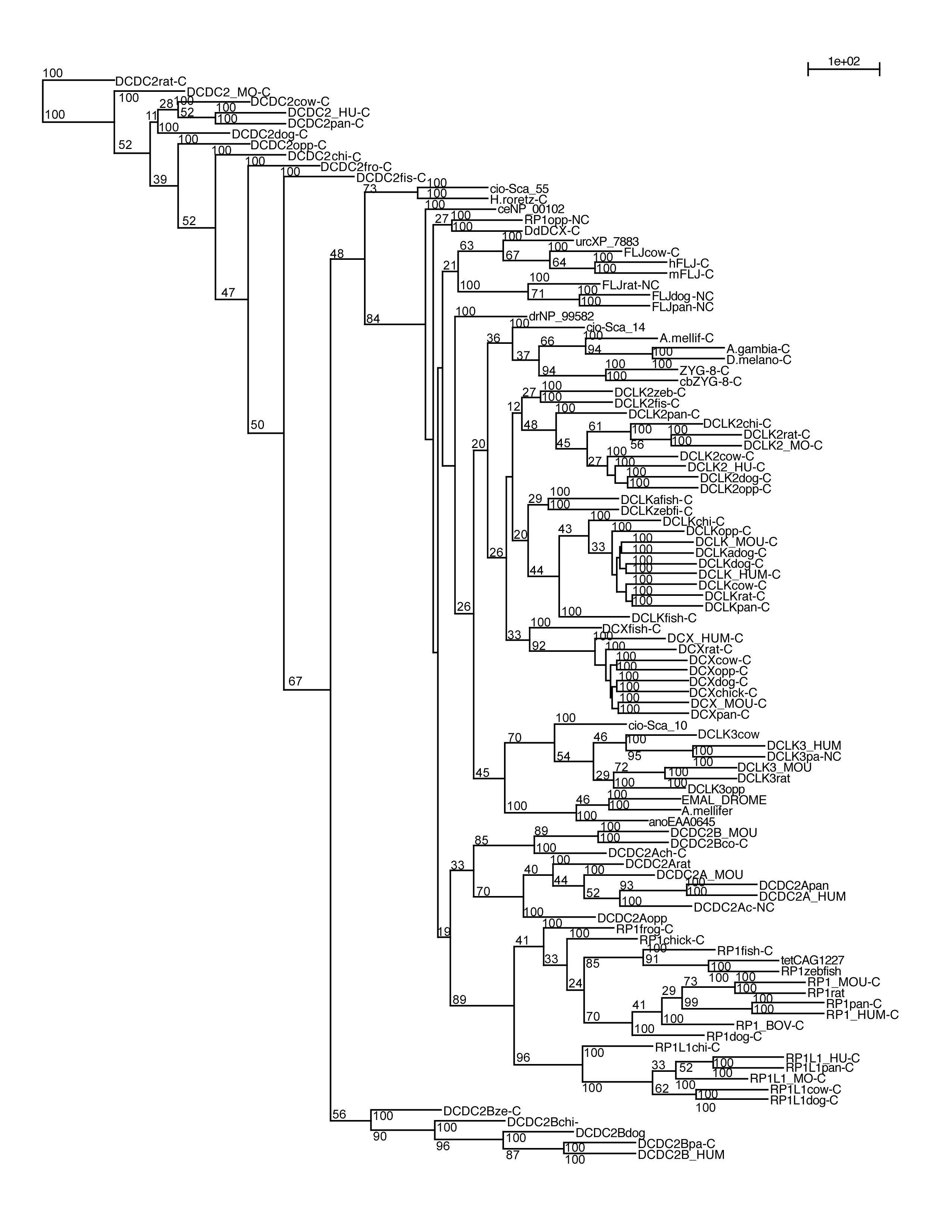

Supplement: Additional File 5 — Supplementary Fig. 5: ML tree of the C-terminal DCX domain proteins from different species. Bootstrap values are indicated. [file 1471-2164-7-188-S5.jpeg]

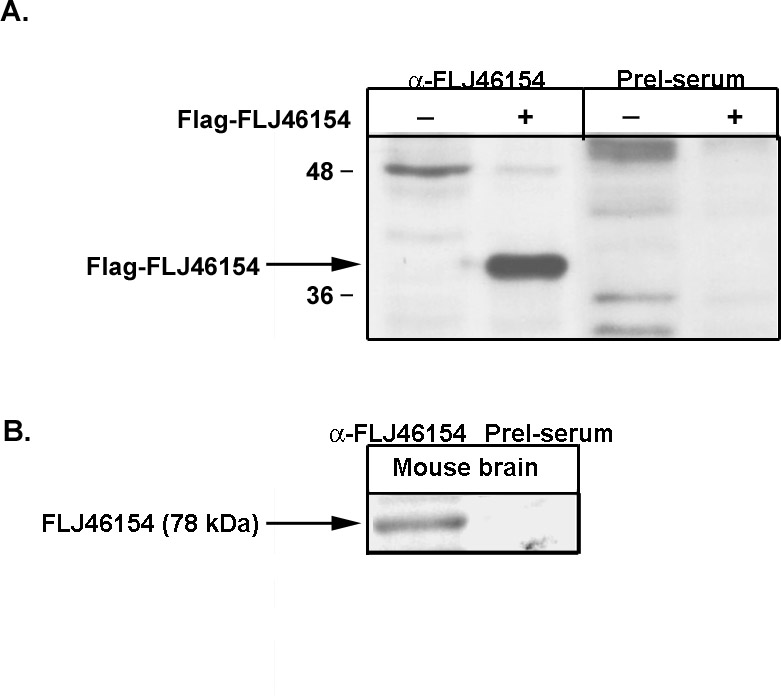

Supplement: Additional File 6 — Supplementary Fig. 6: Western blot analysis using anti-FLJ46154 antibodies. The specificity of the antibodies was verified using cells transfected with Flag-FLJ46154 (A) compared with the preimmune serum. (B) Anti-FLJ46154 antibodies recognize a protein of approximate size 78 kDa in mouse brain extract. [file 1471-2164-7-188-S6.jpeg]
