# Supplementary material for: The evolving doublecortin (DCX) superfamily
Source: BMC Genomics. 2006 Jul 26;7:188. doi: 10.1186/1471-2164-7-188 (PMC1550402; doi:10.1186/1471-2164-7-188)
Supplement: Additional File 7 — Supplementary Fig. 7: Proposed nomenclature for the doublecortin superfamily. [file 1471-2164-7-188-S7.doc]

Proposed nomenclature for the doublecortin superfamily:

RP1 = Dcdc3

RP1L1 = Dcdc4

BAC26042 or 4732421G10Rik (AK028639) = Dcdc5

FLJ46154 (XP_46154) = Dcdc6
